# Design and Synthesis of Dimethylaminomethyl-Substituted Curcumin Derivatives: Potent Anti-Inflammatory, Anti-Oxidant, and Radioprotection Activity, Improved Aqueous Solubility Compared with Curcumin

**DOI:** 10.3390/molecules29091985

**Published:** 2024-04-26

**Authors:** Huiling Gu, Sifan Liu, Kai Liang, Ziming Xia, Guangjie Zhang, Bin Li, Shuchen Liu

**Affiliations:** 1 School of Pharmacy, Henan University, Kaifeng 475001, China; ghl1112@126.com; 2 Department of Pharmaceutical Science, Beijing Institute of Radiation Medicine, Beijing 100850, China; 18146539471@163.com (S.L.); a2724963261@163.com (K.L.); zmxia22@163.com (Z.X.); zhanggj410@sina.com (G.Z.); 3 School of Pharmacy, Guangdong Pharmaceutical University, Guangzhou 510006, China

**Keywords:** curcumin derivatives, anti-inflammatory activity, anti-oxidant activity, radioprotection activity, aqueous solubility

## Abstract

Although the wide variety of bioactivities of curcumin has been reported by researchers, the clinical application of curcumin is still limited due to its poor aqueous solubility. In view of this, a series of dimethylaminomethyl-substituted curcumin derivatives were designed and synthesized (compounds **1**–**15**). Acetate of these derivatives were prepared (compounds **1a**–**15a**). The Mannich reaction and aldol condensation reaction are the main reactions involved in this study. Compounds **6**, **10**, **12**, **3a**, **5a**, **6a**, **7a**, **8a**, **10a**, **11a**, **12a**, **13a**, **14a**, and **15a** exhibited better in vitro anti-inflammatory activity compared to curcumin in the RAW264.7 cell line. Compounds **5**, **1a**, **5a**, **8a**, and **12a** exhibited better in vitro antioxidant activity compared to curcumin in the PC 12 cell line. Compounds **11**, **13**, **5a**, **7a**, and **13a** exhibited better in vitro radiation protection compared to curcumin in the PC 12 cell line. The aqueous solubilities of all the curcumin derivative acetates were greatly improved compared to curcumin.

## 1. Introduction

Curcumin is a natural product isolated from the plant Curcuma Longa L. in the ginger family. Although a wide variety of bioactivities of curcumin have been reported by researchers, including anti-inflammation [1], anti-oxidation [2], anti-radiation [3,4,5], etc., the clinical application of curcumin is still limited due to its poor aqueous solubility (<0.1 mg/mL) [6]. Poor aqueous solubility of drugs can lead to many problems, such as making it difficult for drugs to be made into oral formulations, affecting the metabolism of drugs in the body, reducing the exposure of compounds, and affecting the efficacy of drugs [7,8]. Common methods to improve drug aqueous solubility include salification [9] and the development of prodrugs [10] or drug delivery systems [9,11,12]. Compared to other methods, salt formation is a direct and effective strategy to improve the aqueous solubility of drugs. As for curcumin, the weak acidity of curcumin and its instability in alkaline environments make it difficult for curcumin to form a salt [13]. Dimethylaminomethyl is a common group for structural modification of curcumin. The function of curcumin derivatives containing quaternary amine groups in inducing DNA strand cross-linking and late apoptosis of tumor cells has been the concern of many researchers [14,15,16,17]. Moreover, the basic nitrogen atom of dimethylaminomethyl endows the target compounds with the potential for salt formation, and researchers have designed and synthesized many dimethylaminomethyl-substituted curcumin derivatives to facilitate salt formation. At present, dimethylaminomethyl-substituted curcumin derivatives can be mainly divided into two categories. One is the derivatives in which the methoxy group of curcumin are substituted by dimethylaminomethyl, such as the curcumin derivatives designed and synthesized by Fang X. et al. [14] and Lei F. et al. [18] The aqueous solubility of curcumin derivative salts synthesized by Fang X. et al. was much improved, reaching 367.88 mg/mL. The dimethylaminomethyl-substituted curcumin derivatives synthesized by Lei F. et al. also belong to this category, and showed better in vitro free-radical scavenging ability (DPPH, IC_50_ 1.5–29.9 μM) and aqueous solubility (16.7 mg/mL) compared to curcumin. Another class of dimethylaminomethyl-substituted curcumin derivatives have their hydroxyl ortho hydrogen substituted by dimethylaminomethyl. The curcumin derivatives synthesized by Kurnia A. et al. [15], Liu B. et al. [16], and Dong J. et al. [17] belong to this category. The curcumin derivatives synthesized by Kurnia A. et al. exhibited a potent cytotoxic agent (IC_50_ = 5.70, 5.55, and 2.97 μM) against WiDr (colorectal carcinoma) cells lines. Among the series of curcumin derivatives synthesized by Liu B. et al., curcumin derivatives containing dimethylaminomethyl showed certain anti-tumor activity (IC_50_ = 27.07 μM and 27.96 μM against Hela and MCF-7 cell lines, respectively). The dimethylaminomethyl-substituted curcumin derivatives synthesized by Dong J. et al. exhibited a potent cytotoxic agent (IC_50_ = 1.70 μM) against human chronic myeloid leukemia cells.

**Figure 1:**
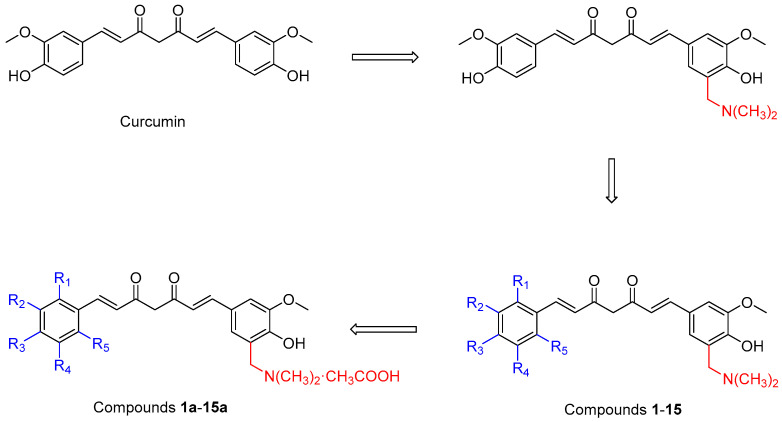
Design strategy of curcumin derivatives.

These studies indicated that the aqueous solubility of dimethylaminomethyl-substituted curcumin derivatives could be greatly increased through the formation of salt. Meanwhile, these derivatives exhibited excellent anti-oxidant and anti-tumor activities. However, the anti-inflammatory and anti-radiation activities of dimethylaminomethyl-substituted curcumin derivatives were not involved in previous studies, and the number of derivatives synthesized was relatively small. In this study, a series of unsymmetric curcumin derivatives, which belongs to the second category of dimethylaminomethyl substituted curcumin derivatives were designed and synthesized. As shown in Figure 1, firstly, the hydrogen atom adjacent to the phenolic hydroxyl group on one aromatic ring of curcumin was replaced by the dimethylaminomethyl group. Then, a series of dimethylaminomethyl-substituted curcumin derivatives (compounds **1**–**15**) were designed by diversifying the substituents on the other aromatic ring. The acetates (compounds **1a**–**15a**) of these derivatives were prepared. Subsequently, various in vitro physiological activities of curcumin derivatives and their acetates were detected and compared with curcumin, including anti-inflammatory activity, anti-oxidant activity, anti-radiation activity, and cytotoxic activity. Finally, aqueous solubilities of these curcumin derivative acetates were measured.

## 2. Results

### 2.1. Synthesis of Dimethylaminomethyl Substituted Curcumin Derivatives and Their Acetates

**Figure 2:**
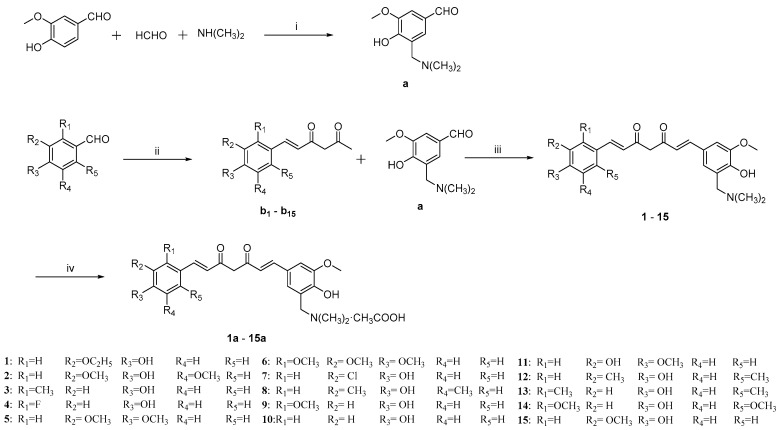
Synthesis of curcumin derivatives **1**–**15** and their acetates **1a**–**15a**. Reagents and conditions: (i) CH_3_OH, 50 °C, overnight; (ii) Acetylacetone, Butylamine, B(OCH_3_)_3_, B_2_O_3_, EtOAc, 80 °C, 4 h; (iii) compound **a**, piperidine, B(OCH_3_)_3_, B_2_O_3_, EtOAc, 80 °C, 4 h; and (iv) CH_3_COOH, rt, overnight.

The synthesis route of the target compounds is shown in Figure 2. Intermediate **a** was synthesized using the method in reference [19]. Vanillin is a commonly used raw material for the synthesis of curcumin and curcumin derivatives. Owing to *p*-π conjugation effect, the hydrogen atom on ortho-position to hydroxyl group in vanillin is very active, so that it is suitable for performing a Mannich reaction to give compound **a**. Meanwhile, according to the method in reference [20], various substituted benzaldehydes were subjected to condensation reaction with 2, 4-pentanedione, respectively, to obtain a series of intermediate **b_1_**–**b_15_**. The synthesis of intermediates **b_1_**–**b_15_** has been reported in the previous literature [21,22,23,24,25,26]. Subsequently, Mannich base **a** underwent condensation reaction with compounds **b_1_**–**b_15_** to synthesize a series of unsymmetric curcumin derivatives, Mannich base **1**–**15**. Acetates of curcumin derivatives were easily prepared (**1a**–**15a**). Compounds **1**–**15** and **1a**–**15a** are new compounds that have not been reported in the literature. It is worth noting that the structural difference between compound **15** and curcumin is only that there is a dimethylaminomethyl substitution at the ortho position of the hydroxyl group of compound **15**. The substituent status of curcumin derivatives **1**–**15** and their acetates **1a**–**15a** is shown in Table 1. Structure data, NMR spectra, and mass spectra of curcumin derivatives **1**–**15** and their acetates **1a**–**15a** are provided in the Supplementary Data.

### 2.2. Cytotoxic Activities

**Figure 3:**
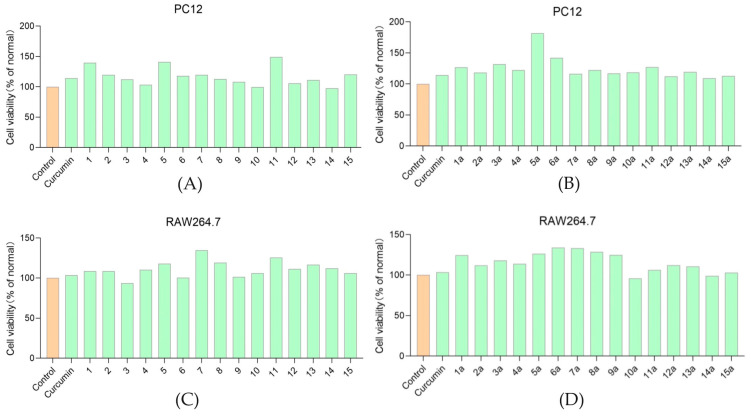
Cytotoxic activities of curcumin derivatives **1**–**15** and their acetates **1a**–**15a** to RAW264.7 cell line and PC 12 cell line at a concentration of 20 μM. Cytotoxic activities of compounds **1**–**15** to RAW264.7 cell line (**A**). Cytotoxic activities of compounds **1a**–**15a** to RAW264.7 cell line (**B**). Cytotoxic activities of compounds **1**–**15** to PC 12 cell line (**C**). Cytotoxic activities of compounds **1a**–**15a** to PC 12 cell line (**D**).

The results show that all the curcumin derivatives **1**–**15** and their acetates **1a**–**15a** failed to exhibit toxicity to the RAW264.7 cell line and PC 12 cell line at a concentration of 20 μM compared to the control (Figure 3), indicating that these compounds were suitable for the subsequent activity screening study. Compounds **1**, **5**, **11**, **5a**, and **6a** exhibit a significant promoting effect on PC 12 cell proliferation at a concentration of 20 μM.

### 2.3. Anti–Inflammatory Activity against NO Release

**Figure 4:**
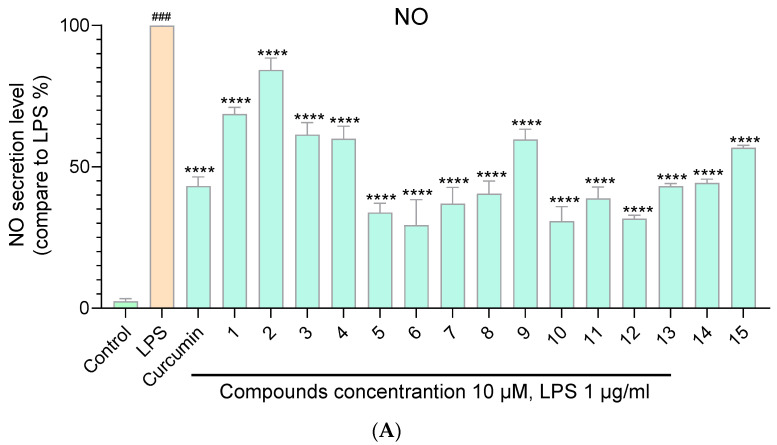

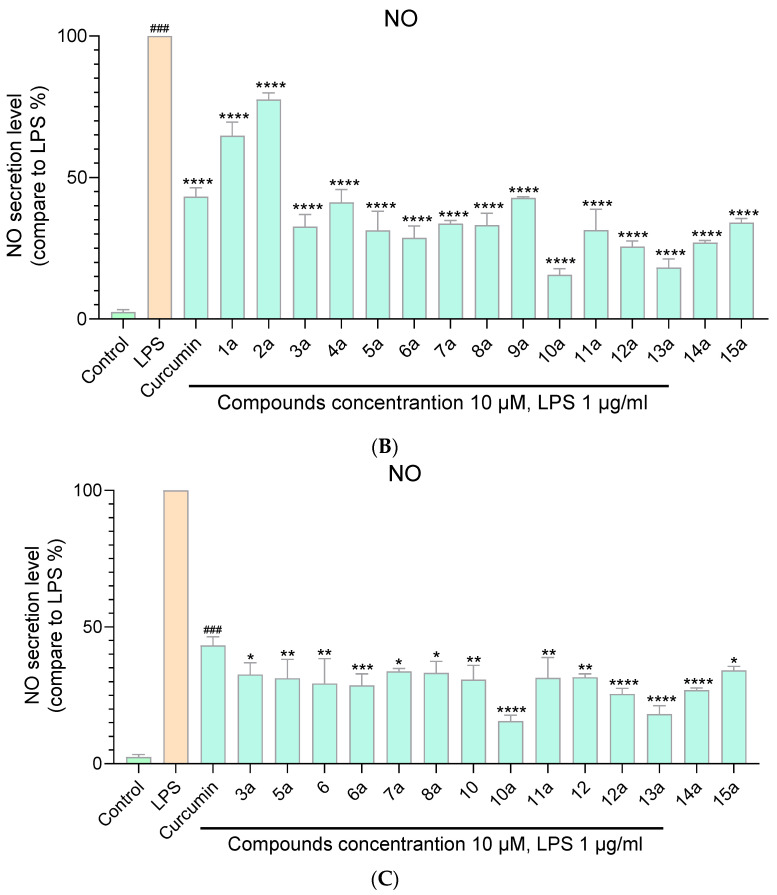
Inhibitory effects of the curcumin derivatives **1**–**15** (**A**) and their acetates **1a**–**15a** (**B**) on LPS-induced NO production. ^###^ The comparison object in the one-way analysis of ANOVA. **** *p* < 0.0001 compared with the LPS-treated group. Comparation of inhibitory effects on LPS-induced NO production between curcumin derivatives (acetates) and curcumin (**C**). ^###^ The comparison object in the one-way analysis of ANOVA. * *p* < 0.1, ** *p* < 0.01, *** *p* < 0.001, **** *p* < 0.0001 compared with curcumin.

**Figure 5:**
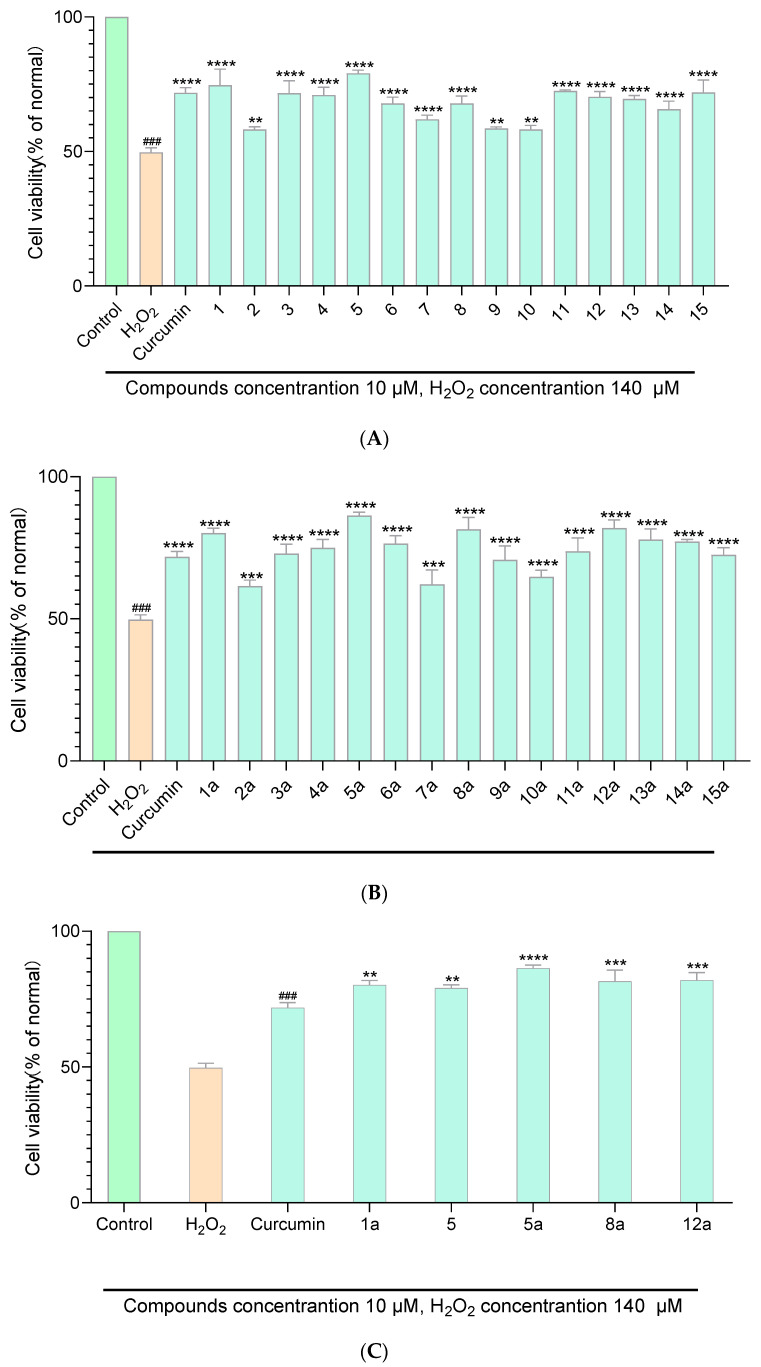
Protection effects of the curcumin derivatives **1**–**15** (**A**) and their acetates **1a**–**15a** (**B**) on H_2_O_2_-induced oxidative damage. ^###^ The comparison object in the one-way analysis of ANOVA. ** *p* < 0.01, *** *p* < 0.001, **** *p* < 0.0001 compared with the LPS-treated group. Comparation of protection effects on H_2_O_2_-induced oxidative damage between curcumin derivatives (acetates) and curcumin (**C**). ^###^ The comparison object in the one-way analysis of ANOVA. ** *p* < 0.01, *** *p* < 0.001, **** *p* < 0.0001 compared with curcumin.

The anti-inflammatory assays of curcumin derivatives **1**–**15** and their acetates **1a**–**15a** were evaluated in the LPS–stimulated RAW264.7 cell lines with curcumin as the positive control. Raw data of anti-inflammatory assays is shown in Table S1 in the Supplementary Material. Data were represented as mean ± SD of three independent experiments. The statistical analyses were performed using the one-way analysis of ANOVA in GraphPad Prism 8. As shown in Figure 4A,B, all the compounds exhibited a certain degree of inhibition of NO release at a concentration of 10 μM. Among the curcumin derivatives **1**–**15** and their acetates **1a**–**15a**, compound **10a** exhibited the most significant inhibition effect (inhibition rate 92% at 15 μM) on NO release (Table S1). Compounds **6**, **10**, **12**, **3a**, **5a**, **6a**, **7a**, **8a**, **10a**, **11a**, **12a**, **13a**, **14a**, and **15a** exhibited better inhibitory activity against NO production compared with curcumin at a concentration of 10 μM (Figure 5C). Most of the target compounds inhibit the release of NO in a dose-dependent manner (Table S1).

### 2.4. Antioxidant Capacity Evaluation

The anti-oxidant assays of curcumin derivatives **1**–**15** and their acetates **1a**–**15a** were evaluated in H_2_O_2_–stimulated PC 12 cell lines with curcumin as the positive control. Raw data of anti-oxidant assays is shown in Table S2 in the Supplementary Material. Data were represented as mean ± SD of three independent experiments. The statistical analyses were performed using the one-way analysis of ANOVA in GraphPad Prism 8. As shown in Figure 5A,B, curcumin derivatives **1**–**15** and their acetates **1a**–**15a** exhibited a certain degree of antioxidant capacity at a concentration of 10 μM. Compounds **5**, **1a**, **5a**, **8a**, and **12a** exhibited better anti-oxidant capacity compared with curcumin at a concentration of 10 μM (Figure 5C). Among the synthesized curcumin derivatives and their acetates**,** compound **5a** showed the best anti-oxidant effect, with a cell survival rate of 106% at 20 μM (Table S2). Most of the target compounds shown an anti-oxidant capacity in a dose-dependent manner. Compounds **2a**, **7a**, and **10a** had a relatively poor anti-oxidant capacity (with cell survival rate of 61%, 62%, and 64% at 10μM, respectively).

### 2.5. Radiation Protection Capability

**Figure 6:**
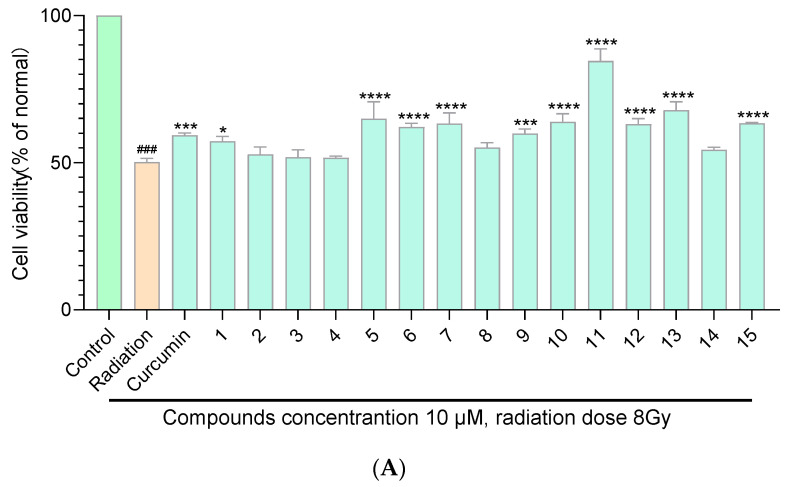

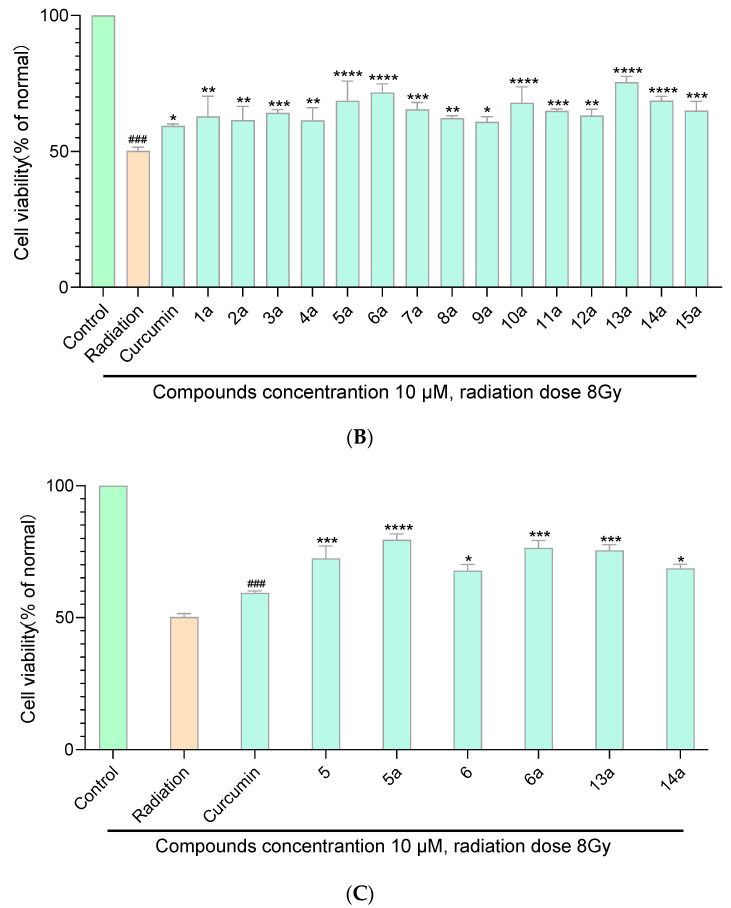
Protection effects of the curcumin derivatives **1**–**15** (**A**) and their acetates **1a**–**15a** (**B**) on radiation. ^###^ The comparison object in the one-way analysis of ANOVA. * *p* < 0.1, ** *p* < 0.01, *** *p* < 0.001, **** *p* < 0.0001 compared with the LPS-treated group. Comparation of protection effects on H_2_O_2_-induced oxidative damage between curcumin derivatives (acetates) and curcumin (**C**). ^###^ The comparison object in the one-way analysis of ANOVA. * *p* < 0.1, *** *p* < 0.001, **** *p* < 0.0001 compared with curcumin.

The anti-radiation assays of curcumin derivatives **1**–**15** and their acetates **1a**–**15a** were evaluated in PC 12 cell lines with curcumin as the positive control. Raw data of anti-radiation assays is shown in Table S3 in the Supplementary Material. Data were represented as mean ± SD of three independent experiments. The statistical analyses were performed using the one-way analysis of ANOVA in GraphPad Prism 8. As shown in Figure 6A,B, compounds **1**, **5**, **6**, **8**, **11**, **12**, **1a**, **5a**, **6a**, **8a**, **11a**, **12a**, **13a**, and **14a** exhibited a certain degree of radiation protection capacity at a concentration of 10 μM. Compounds **6**, **5a**, **6a**, **13a**, and **14a** exhibited better radiation protection capability compared with curcumin at a concentration of 10 μM (Figure 6C). Among the synthesized curcumin derivatives and their acetates, compound **11a** has the best radiation protection ability, with a cell survival rate of 106% at 20 μM (Table S3 in the Supplementary Material). Most of the target compounds showed a radiation protection capacity in a dose-dependent manner.

### 2.6. Aqueous Solubility of Curcumin Derivative Acetates

Aqueous solubility of curcumin-derivative acetates **1a**–**15a** were measured using an UV spectrophotometer. The optical density–concentration curves of compounds **1a**–**15a** are shown in Figures S76–S90, and the saturated solution concentrations of curcumin derivative acetates **1a**–**15a** are shown in Table 2. Compared with the solubility of curcumin (<0.1 mg/mL), those of the curcumin-derivative acetates **1a**–**15a** were significantly improved. Among the test compounds, compound **10a** has the best aqueous solubility, 335.16 mg/mL, which is similar to the solubility (367.88 mg/mL, 305.67 mg/mL) of dimethylaminomethyl substituted curcumin derivative salts in previous study [14].

## 3. Discussion

### 3.1. The Effect of Introducing Dimethylaminomethyl Group on the Bioactivity of Curcumin

Compound **15** is a derivative of curcumin where the hydroxyl ortho position is substituted by a dimethylaminomethyl group. Thus, the impact of introducing dimethylaminomethyl can be demonstrated by comparing the activity data of compound **15** and curcumin. It is found that the introduction of dimethylaminomethyl group did not increase the toxicity of curcumin derivatives on RAW264.7 and PC12 cell lines (Figure 3). The anti-oxidant (Table S2) and anti-radiation abilities (Table S3) of compound **15** and curcumin had no statistical difference. Fang X. et al. replaced the methoxy group in curcumin with dimethylaminomethyl group, and concluded that the compound’s in vitro free radical scavenging ability (IC_50_ 18.6 μM, while that of curcumin was 26.5 μM) was enhanced [14], indicating that the different substitution positions of dimethylaminomethyl group may have an impact on anti-oxidant activity. It is worth noting that the anti-inflammatory activity was slightly decreased after the introduction of the dimethylaminomethyl group (Table S1).

### 3.2. Effect of Modification of Aromatic Ring Substituents on Biological Activity of Dimethylamino-Methyl Group Substituted Curcumin Derivatives

Comparing the anti-inflammatory activity of compound **10** (inhibition rate 71% at 10 μM) with that of compounds **9** (inhibition rate 41% at 10 μM) and **15** (inhibition rate 44% at 10 μM), it was found that methoxy substituent at the ortho and meta positions of the hydroxyl group may reduce the anti-inflammatory activity of the compound. In addition, when R_1_ and R_5_ (compound **14**) or R_2_ and R_4_ (compound **2**) were both substituted with methoxy, it could also lead to a decrease in anti-inflammatory activity.

There is no obvious rule that the antioxidant activity changes with the aromatic ring structure. Interestingly, compound **5a** exhibited both excellent anti-oxidant (survival rate 106% at 20 μM) and anti-radiation activities (cell survival rate 104% at 15 μM), which may be due to its capacity to promote the proliferation of PC12 cell line (Figure 3). Similarly, compound **11** could promote the proliferation of the PC12 cell line, and showed good anti-radiation (cell survival rate 94% at 20 μM) and anti-oxidant activities (cell survival rate 96% at 20 μM).

### 3.3. Effect of Salt Formation on the Biological Activity and Aqueous Solubility of Curcumin Deriva-Tives

Comparing the bioactivity of curcumin-derivative acetates with that of curcumin derivatives themselves (Tables S1–S3), it was found that salt formation significantly increased the aqueous solubility of the compounds and had almost no negative effect on their physiological activities, such as their anti-inflammatory, anti-oxidant, and anti-radiation activities.

Although there is often no statistically significant difference between the bioactivity of curcumin-derivative acetates and curcumin derivatives themselves, salt formation increases the significance of difference in activity between some curcumin derivatives and curcumin. For example, the significance of difference in antioxidant activity between curcumin derivative **5** and curcumin was *p* = 0.0054, and the significance of difference between its acetate **5a** and curcumin was *p* < 0.0001 (Figure 5C).

## 4. Materials and Methods

### 4.1. General Experimental Procedures

All chemicals were obtained from suppliers (Sigma-Adrich, St. Louis, MO, USA, TCI, Ark). NMR spectra were recorded on a Bruker ECA-400 MHz (Bruker Co. Billerica, MA, USA) spectrometer with TMS as an internal standard. Chemical shifts were in ppm (δ), and coupling constants (J) were reported in Hertz (Hz). High-resolution electrospray ionization mass spectra (HR-ESI-MS) was performed on the Agilent G6230A TOF LC/MS (Agilent Technologies Co. Ltd., Santa Clara, CA, USA) using ESI ion source. Absorbances in vitro experiments were measured using a spectrophotometer (Thermo, Singapore Republic of Singapore). Column chromatography (CC) was performed using silica gel (Qingdao Marine Chemical Inc., Qingdao, China). Thin-layer chromatography (TLC) was performed with silica gel GF254 (Yantai Huayang New Material Technology Co., Ltd., Yantai, China). Data of in vitro experiments were analyzed and plotted using Graghpad Prism (version 8, GraphPad Software, San Diego, California, 2020).

### 4.2. Procedure for the Synthesis of Target Compounds

#### 4.2.1. Synthesis of 3-((Dimethylamino)methyl)-4-Hydroxy-5-Methoxybenzaldehyde (**a**)

Intermediate **a** was synthesized using the method from reference [19]. To a mixture of dimethylamine (40%, 1 g, 1 eq) and HCHO (37%, 1.1 g, 1.5 eq) at 70 °C, vanillin (0.68 g, 0.5 eq) in MeOH (20 mL) was added in batches. The reaction mixture was stirred at 70 °C in air for 12 h. After the completion of the reaction (monitored using TLC), the reaction mixture was evaporated under reduced pressure. The residue was quenched with H_2_O (20 mL) and extracted with EtOAc (3 × 20 mL). The organic layers were dried over anhydrous Na_2_SO_4_ and evaporated in vacuo. The residue was purified using flash column chromatography on silica gel (petroleum ether/EtOAc = 4:1) to afford the compound **a**.

#### 4.2.2. Synthesis of Compounds **b_1_**–**b_15_**

Intermediates **b_1_**–**b_15_** were synthesized by the method from reference [20].To a solution of acetylacetone (0.9 g, 1 eq) in EA (10 mL), boron trioxide (0.21 g, 0.33 eq) was added. The reaction mixture was stirred at 80 °C for 0.5 h. Then, the solution of benzaldehydes (0.33 eq) and trimethyl borate (0.31 g, 0.33 eq) in EA (10 mL) was added to the mixture above. The reaction mixture was stirred at 80 °C for 0.5 h before n-butylamine (0.22 g, 0.33 eq) was added. The reaction mixture was stirred at 100 °C for another 0.5 h before HCl (1 N, 5 mL) was added. After the completion of the reaction (monitored by TLC), the reaction was quenched with H_2_O (20 mL) and extracted with EtOAc (3 × 20 mL). The organic layers were dried over anhydrous Na_2_SO_4_ and evaporated in vacuo. The residue was purified by flash column chromatography on silica gel (petroleum ether/EtOAc = 4:1), respectively, to afford the compounds **b_1_**–**b_15_**.

#### 4.2.3. Synthesis of Compounds **1**–**15** and **1a**–**15a**

Compounds **1**–**15** were synthesized by the method from reference [20]. To a solution of compounds **b_1_**–**b_15_** (1 mmol, 1 eq) in EA (3 mL), boron trioxide (21 mg, 0.33 eq) was added, respectively. The reaction mixture was stirred at 80 °C for 0.5 h. Then, the solution of compound **a** (70 mg, 0.33 eq) and trimethyl borate (31 mg, 0.33 eq) in EA (3 mL) was added to the mixture above. The reaction mixture was stirred at 80 °C for 0.5 h before n-butylamine (22 mg, 0.33 eq) was added. The reaction mixture was stirred at 100 °C for another 0.5 h before HCl (1 N, 2 mL) was added. After completion of the reaction (monitored by TLC), the reaction was quenched with H_2_O (20 mL) and extracted with EtOAc (3 × 20 mL). The organic layers were dried over anhydrous Na_2_SO_4_ and evaporated in vacuo. The residue was purified by flash column chromatography on silica gel (petroleum ether/EtOAc = 4:1), respectively, to afford the compounds **1**–**15**. Compounds **1a**–**15a** were obtained by treating compounds **1**–**15** with excessive CH_3_COOH in acetonitrile.

### 4.3. Cytotoxicity Assay

The cytotoxicity assay was performed using the Cell Counting Kit-8 (CCK-8, Applygen Technologies Co., Ltd., Beijing, China) assay on murine macrophage cell line (RAW264.7) and the rat adrenal pheochromocytoma cells (PC 12) using curcumin as positive controls, respectively. RAW264.7 cells were cultured in 96-well plates at a density of 3 × 10^4^ cells/mL in Dulbecco’s modified Eagle’s medium (DMEM, Gibco, Baltimore, MD, USA) with 10% fetal calf serum (Gibco, Baltimore, MD, USA), 100 U/mL penicillin, and 100 μg/mL streptomycin. Plates were incubated in an atmosphere containing 5% CO_2_ at 37 mL. PC 12 cells were cultured in 96-well plates at a density of 8000 cells/mL in Dulbecco’s modified Eagle’s medium (DMEM, Gibco, Baltimore, MD, USA) with 10% fetal calf serum (Gibco, Baltimore, MD, USA), 100 U/mL penicillin, and 100 μg/mL streptomycin. Plates were incubated in an atmosphere containing 5% CO_2_ at 37 °C. Cells were treated with different concentrations of test compounds for 48 h. Then, 10 μL CCK-8 solution were added to all the wells. After 4 h incubation, the absorbance at 450 nm of each well was measured in a multiscan photometer.

### 4.4. Determination of NO Content

RAW 264.7 cells were plated in 96-well plates at a density of 1 × 10^4^ cells/well and incubated overnight at 37 °C under 5% CO_2_. Then, the cells were treated with compounds **1**–**15** and **1a**–**15a** at a concentration of 10 µM, respectively for 1 h and then stimulated with LPS (1 µg/mL). Dexamethasone (Solarbio, D8040, Beijing, China) was used as the positive control. Then, 24 h later, NO production was determined by measuring the nitrite content using Griess reagents. Fifty microliters of culture supernatants were transferred to 96-well plates and mixed with 50 μL Griess reagent A. After incubation at room temperature for 5 min, the samples were mixed with an equal volume Griess reagent B for another 5 min. The absorbance of each mixture at 540 nm was measured using a microplate reader.

### 4.5. Antioxidant Activity Effect Evaluation

PC 12 cells were seeded in 96-well plates at a density of 8000 cells/well and incubated overnight. Then, the cells were treated with target compounds at a concentration of 10 μM, respectively for 1 h, and then stimulated with H_2_O_2_ (140 μM). After incubating under the same conditions for 24 h, 10 μL CCK-8 solution was added to each well. After 2 h incubation, the absorbance of each well was measured at 450 nm using a microplated reader (Multiskan MK-3, Thermo, Waltham, MA, USA).

### 4.6. Radiation Protection In Vitro

The rat adrenal pheochromocytoma cells (PC 12) were used for in vitro studies. A Cell Counting Kit-8 (CCK-8, Applygen Technologies Co., Ltd., Beijing, China) assay was used to determine cell viability. Cells were seeded in 96-well plates with a density of 8 × 10^3^ cells/ well and treated with curcumin and different concentrations of test compounds (10 µM) for 24 h before irradiation. Cells were irradiated with dose of 8.0 Gy at a dose rate of 0.8 Gy/min. Then, 10 μL CCK-8 solution was added to all the wells. After 4 h incubation, the absorbance at 450 nm of each well was measured in a multiscan photometer.

### 4.7. Aqueous Solubility Determination

A UV spectrophotometer was used to test the aqueous solubility of target compounds. Each compound to be tested was prepared into solutions with concentrations of 0.2 M, 0.1 M, 0.05 M, 0.025 M, 0.0125 M, 0.00625 M, and 0.003125 M. The optical density (OD) values of the prepared solutions were measured using an ultraviolet spectrophotometer, and concentration–OD-value standard curves were drawn for each compound to be tested. The saturated solutions of the target compounds were diluted by 5000 times and detected using an ultraviolet spectrophotometer to measure the OD values, respectively. The measured OD values were substituted into the corresponding concentration-OD value standard curve to calculate the diluted concentrations, which were multiplied by 5000 to obtain the saturated solution concentrations of the target compounds.

### 4.8. Statistical Analysis

In vitro experimental data were represented as mean ± SD of three independent experiments. The statistical analyses were performed using the one-way analysis of ANOVA in GraphPad Prism 8.

## 5. Conclusions

In summary, a series of dimethylaminomethyl-substituted curcumin derivatives were designed and synthesized (compounds **1**–**15**). Acetate of these derivatives were prepared (compounds **1a**–**15a**). The various physiological activities of curcumin derivatives and their acetate were tested in vitro and compared with curcumin, including anti-inflammatory activity, anti-oxidant activity, anti-radiation activity, and cytotoxic activities. Finally, aqueous solubility of these curcumin derivative acetates was measured. Compounds **6**, **10**, **12**, **3a**, **5a**, **6a**, **7a**, **8a**, **10a**, **11a**, **12a**, **13a**, **14a**, and **15a** exhibited significantly better anti-inflammatory activity than curcumin, and could be further developed as anti-inflammatory lead compounds. Compounds **5**, **1a**, **5a**, **8a**, and **12a** showed significantly better anti-oxidant activity than curcumin, and could be further developed as anti-oxidant lead compounds. Compounds **6**, **5a**, **6a**, **13a**, and **14a** exhibited significantly better anti-radiation activity than curcumin, and could be further developed as anti-radiation lead compounds. Those compounds with more than two kinds of physiological activities deserve more attention of researchers, such as compounds **6**, **5a**, **6a**, **8a**, **12a**, and **14a**. The aqueous solubility of curcumin-derivative acetates (112.26–335.36 mg/mL) was greatly improved compared with that of curcumin (<0.1 mg/mL).

## Figures and Tables

**Table 1:** Synthesis of curcumin derivatives **1**–**15** and their acetates **1a**–**15a**.

Compound	The Left Arene Ring	Compound	The Left Arene Ring	Compound	The Left Arene Ring
**1**, **1a**	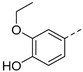	**6**, **6a**	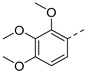	**11**, **11a**	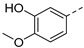
**2**, **2a**	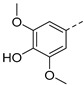	**7**, **7a**	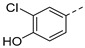	**12**, **12a**	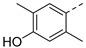
**3**, **3a**	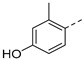	**8**, **8a**	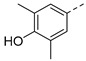	**13**, **13a**	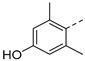
**4**, **4a**	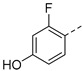	**9**, **9a**	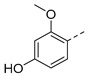	**14**, **14a**	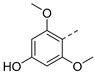
**5**, **5a**	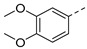	**10**, **10a**	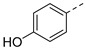	**15**, **15a**	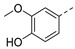

**Table 2:** Aqueous solubility of curcumin derivative acetates **1a**–**15a**.

Compound	OD ^1^	Solubility (mg/mL)
**1a**	0.34	201.06
**2a**	0.71	148.19
**3a**	0.66	254.95
**4a**	0.51	314.20
**5a**	0.30	117.88
**6a**	0.23	112.26
**7a**	0.58	257.90
**8a**	0.61	118.16
**9a**	0.94	167.04
**10a**	0.84	335.16
**11a**	0.51	132.20
**12a**	0.43	206.04
**13a**	0.74	285.48
**14a**	0.47	126.01
**15a**	0.72	283.81

^1^ The optical density values were recorded at 384 nm, and the values of compounds **1a**–**15a** were measured at the 5 × 10^3^ fold diluted saturated concentration, since the values of the saturated solution were too high to be measured.

## Data Availability

The data presented in this study are available in the Supplementary Materials.

## Supplementary Materials

The following supporting information can be downloaded at: https://www.mdpi.com/article/10.3390/molecules29091985/s1, Table S1. Inhibitory effects of the curcumin derivatives **1**–**15** and their acetates **1a**–**15a** on LPS-induced NO production. Table S2. Protection effects of the curcumin derivatives **1**–**15** and their acetates **1a**–**15a** on H_2_O_2_-induced oxidative damage. Table S3. Protection effects of the curcumin derivatives **1**–**15** and their acetates **1a**–**15a** on Radiation. 1 Figure S1: ^1^H-NMR spectrum of **1** in CD_3_OD. Figure S2: ^13^C-NMR spectrum of **1** in DMSO-*d*_6_. Figure S3: HR-ESI-MS spectrum of **1.** Figure S4: ^1^H-NMR spectrum of **2** in DMSO-*d*_6_. Figure S5: ^13^C-NMR spectrum of **2** in DMSO-*d*_6_. Figure S6: HR-ESI-MS spectrum of **2.** Figure S7: ^1^H-NMR spectrum of **3** in CD_3_OD. Figure S8: ^13^C-NMR spectrum of **3** in CD_3_OD. Figure S9: HR-ESI-MS spectrum of **3**. Figure S10: ^1^H-NMR spectrum of **4** in CD_3_OD. Figure S11: ^13^C-NMR spectrum of **4** in CD_3_OD. Figure S12: HR-ESI-MS spectrum of **4.** Figure S13: ^1^H-NMR spectrum of **5** in CD_3_OD. Figure S14: ^13^C-NMR spectrum of **5** in CD_3_OD. Figure S15: HR-ESI-MS spectrum of **5.** Figure S16: ^1^H-NMR spectrum of **6** in CD_3_OD. Figure S17: ^13^C-NMR spectrum of **6** in DMSO-*d*_6_. Figure S18: HR-ESI-MS spectrum of **6**. Figure S19: ^1^H-NMR spectrum of **7** in DMSO-*d*_6_. Figure S20: ^13^C-NMR spectrum of **7** in DMSO-*d*_6_. Figure S21: HR-ESI-MS spectrum of **7**. Figure S22: ^1^H-NMR spectrum of **8** in CD_3_OD. Figure S23: ^13^C-NMR spectrum of **8** in CD_3_OD. Figure S24: HR-ESI-MS spectrum of **8**. Figure S25: ^1^H-NMR spectrum of **9** in CD_3_OD. Figure S26: ^13^C-NMR spectrum of **9** in DMSO-*d*_6_. Figure S27: HR-ESI-MS spectrum of **9**. Figure S28: ^1^H-NMR spectrum of **10** in CD_3_OD. Figure S29: ^13^C-NMR spectrum of **10** in CD_3_OD. Figure S30: HR-ESI-MS spectrum of **10**. Figure S31: ^1^H-NMR spectrum of **11** in CD_3_OD. Figure S32: ^13^C-NMR spectrum of **11** in CD_3_OD. Figure S33: HR-ESI-MS spectrum of **11**. Figure S34: ^1^H-NMR spectrum of **12** in DMSO-*d*_6_. Figure S35: ^13^C-NMR spectrum of **12** in CD_3_OD. Figure S36: HR-ESI-MS spectrum of **12**. Figure S37: ^1^H-NMR spectrum of **13** in CD_3_OD. Figure S38: ^13^C-NMR spectrum of **13** in CD_3_OD. Figure S39: HR-ESI-MS spectrum of **13**. Figure S40: ^1^H-NMR spectrum of **14** in CD_3_OD. Figure S41: ^13^C-NMR spectrum of **14** in CD_3_OD. Figure S42: HR-ESI-MS spectrum of **14**. Figure S43: ^1^H-NMR spectrum of **15** in DMSO-*d*_6_. Figure S44: ^13^C-NMR spectrum of **15** in DMSO-*d*_6_. Figure S45: HR-ESI-MS spectrum of **15**. Figure S46: ^1^H-NMR spectrum of **1a** in CD_3_OD. Figure S47: HR-ESI-MS spectrum of **1a**. Figure S48: ^1^H-NMR spectrum of **2a** in CD_3_OD. Figure S49: HR-ESI-MS spectrum of **2a**. Figure S50: ^1^H-NMR spectrum of **3a** in CD_3_OD. Figure S51: HR-ESI-MS spectrum of **3a**. Figure S52: ^1^H-NMR spectrum of **4a** in CD_3_OD. Figure S53: HR-ESI-MS spectrum of **4a**. Figure S54: ^1^H-NMR spectrum of **5a** in CD_3_OD. Figure S55: HR-ESI-MS spectrum of **5a**. Figure S56: ^1^H-NMR spectrum of **6a** in CD_3_OD. Figure S57: HR-ESI-MS spectrum of **6a.** Figure S58: ^1^H-NMR spectrum of **7a** in CD_3_OD. Figure S59: HR-ESI-MS spectrum of **7a**. Figure S60: ^1^H-NMR spectrum of **8a** in CD_3_OD. Figure S61: HR-ESI-MS spectrum of **8a.** Figure S62: ^1^H-NMR spectrum of **9a** in CD_3_OD. Figure S63: HR-ESI-MS spectrum of **9a**. Figure S64: ^1^H-NMR spectrum of **10a** in CD_3_OD. Figure S65: HR-ESI-MS spectrum of **10a**. Figure S66: ^1^H-NMR spectrum of **11a** in CD_3_OD. Figure S67: HR-ESI-MS spectrum of **11a**. Figure S68: ^1^H-NMR spectrum of **12a** in DMSO-*d*_6_. Figure S69: HR-ESI-MS spectrum of **12a**. Figure S70: ^1^H-NMR spectrum of **13a** in CD_3_OD. Figure S71: HR-ESI-MS spectrum of **13a**. Figure S72: ^1^H-NMR spectrum of **14a** in CD_3_OD. Figure S73: HR-ESI-MS spectrum of **14a**. Figure S74: ^1^H-NMR spectrum of **15a** in CD_3_OD. Figure S75: HR-ESI-MS spectrum of **15a**. Figure S75. HR-ESI-MS spectrum of **15a**. Figure S76. Concentration-absorbance curve of **1a**. Figure S77. Concentration-absorbance curve of **2a**. Figure S78. Concentration-absorbance curve of **3a**. Figure S79. Concentration-absorbance curve of **4a**. Figure S80. Concentration-absorbance curve of **5a**. Figure S81. Concentration-absorbance curve of **6a**. Figure S82. Concentration-absorbance curve of **7a**. Figure S83. Concentration-absorbance curve of **8a**. Figure S84. Concentration-absorbance curve of **9a**. Figure S85. Concentration-absorbance curve of **10a**. Figure S86. Concentration-absorbance curve of **11a**. Figure S87. Concentration-absorbance curve of **12a**. Figure S88. Concentration-absorbance curve of **13a**. Figure S89. Concentration-absorbance curve of **14a**. Figure S90. Concentration-absorbance curve of **15a**.
