# Supplementary material for: Design and Synthesis of Dimethylaminomethyl-Substituted Curcumin Derivatives: Potent Anti-Inflammatory, Anti-Oxidant, and Radioprotection Activity, Improved Aqueous Solubility Compared with Curcumin
Source: Molecules. 2024 Apr 26;29(9):1985. doi: 10.3390/molecules29091985 (PMC11085442; doi:10.3390/molecules29091985)
Supplement: Supplementary file 1 [file molecules-29-01985-s001.zip › molecules-2968197-supplementary.pdf]

Supplementary Material

Section 1: Raw data for in vitro activity evaluation.

Table S1. Inhibitory effects of the curcumin derivatives **1-15** and their acetates **1a-15a** on LPS-induced NO production.

Group	The production of NO			
	Dose (5 μM)	Dose (10 μM)	Dose (15 μM)	Dose (0 μM)
Control				1.20 ± 0.38
LPS				49.86 ± 0.62
Curcumin	37.00 ± 1.12	21.55 ± 1.29	5.96 ± 0.03	
Compound 1	44.55 ± 0.55	34.25 ± 0.96	12.30 ± 0.90	
Compound 2	43.78 ± 1.52	42.04 ± 1.70	36.99 ± 1.85	
Compound 3	39.26 ± 2.12	30.62 ± 1.71	10.36 ± 3.69	
Compound 4	40.64 ± 1.28	29.91 ± 1.78	10.48 ± 0.47	
Compound 5	34.62 ± 0.76	16.89 ± 1.33	9.97 ± 1.11	
Compound 6	27.16 ± 2.73	14.67 ± 3.66	5.95 ± 0.13	
Compound 7	35.15 ± 2.72	18.46 ± 2.34	10.47 ± 1.32	
Compound 8	42.90 ± 0.85	20.21 ± 1.82	5.46 ± 0.34	
Compound 9	42.38 ± 2.73	29.76 ± 1.48	19.69 ± 0.78	
Compound 10	36.66 ± 2.00	15.36 ± 2.09	4.91 ± 0.77	
Compound 11	35.85 ± 0.30	19.36 ± 1.66	7.59 ± 0.35	
Compound 12	32.06 ± 0.58	15.78 ± 0.50	11.39 ± 1.00	
Compound 13	26.57 ± 1.74	21.53 ± 0.35	10.02 ± 0.61	
Compound 14	27.84 ± 1.41	22.09 ± 0.55	9.39 ± 0.34	
Compound 15	41.85 ± 1.07	28.31 ± 0.33	14.00 ± 0.92	
Compound 1a	44.93 ± 0.35	32.32 ± 1.94	5.77 ± 0.09	
Compound 2a	41.89 ± 1.95	38.68 ± 0.96	36.24 ± 0.50	
Compound 3a	32.92 ± 2.00	16.27 ± 1.74	5.29 ± 0.97	

Compound 4a	31.54 ± 1.30	20.51 ± 1.88	9.45 ± 1.96	
Compound 5a	33.90 ± 1.94	15.60 ± 2.78	9.88 ± 0.53	
Compound 6a	27.02 ± 2.91	14.29 ± 1.71	5.23 ± 0.72	
Compound 7a	35.35 ± 1.59	16.83 ± 0.42	9.77 ± 0.63	
Compound 8a	35.08 ± 2.53	16.57 ± 1.70	4.61 ± 0.29	
Compound 9a	36.91 ± 0.59	21.33 ± 0.19	11.29 ± 0.25	
Compound 10a	24.20 ± 1.36	7.81 ± 0.85	3.91 ± 0.22	
Compound 11a	35.82 ± 1.34	15.68 ± 3.02	4.63 ± 0.40	
Compound 12a	18.38 ± 1.63	12.76 ± 0.83	9.05 ± 0.72	
Compound 13a	17.80 ± 2.19	9.09 ± 1.21	5.58 ± 0.11	
Compound 14a	29.10 ± 0.37	13.47 ± 0.31	7.49 ± 0.40	
Compound 15a	33.79 ± 2.90	17.01 ± 0.60	4.71 ± 0.18	

Table S2. Protection effects of the curcumin derivatives **1-15** and their acetates **1a-15a** on H_2O_2-induced oxidative damage.

Group	Cell viability			
	Dose (5 μM)	Dose (10 μM)	Dose (20 μM)	Dose (0 μM)
Control				100
H_2O_2				49.69 ± 1.37
Curcumin	59.57 ± 2.21	71.83 ± 1.52	86.38 ± 1.28	
Compound 1	63.89 ± 2.86	74.67 ± 4.82	86.42 ± 3.89	
Compound 2	55.71 ± 0.79	58.21 ± 0.80	63.41 ± 1.13	
Compound 3	64.73 ± 2.69	71.68 ± 3.77	81.68 ± 2.41	
Compound 4	61.70 ± 2.59	70.99 ± 2.33	84.68 ± 2.76	
Compound 5	71.02 ± 0.56	84.44 ± 1.86	102.52 ± 3.36	
Compound 6	56.61 ± 1.66	67.85 ± 1.91	94.89 ± 0.58	
Compound 7	54.43 ± 1.18	61.94 ± 1.29	78.25 ± 1.43	
Compound 8	64.58 ± 1.64	67.81 ± 2.26	81.67 ± 3.21	
Compound 9	53.22 ± 0.26	58.53 ± 0.46	66.09 ± 1.09	

Compound 10	53.53 ± 2.28	58.20 ± 1.24	77.80 ± 2.65	
Compound 11	63.24 ± 1.07	72.52 ± 0.32	96.33 ± 1.04	
Compound 12	60.69 ± 1.53	70.31 ± 1.65	79.23 ± 1.18	
Compound 13	62.94 ± 0.50	69.55 ± 1.06	80.61 ± 0.83	
Compound 14	59.32 ± 2.89	65.73 ± 2.40	74.30 ± 1.25	
Compound 15	60.18 ± 1.41	71.98 ± 3.71	87.82 ± 1.77	
Compound 1a	66.25 ± 1.36	80.18 ± 1.38	89.36 ± 1.63	
Compound 2a	56.60 ± 0.54	61.51 ± 1.77	65.65 ± 1.31	
Compound 3a	61.60 ± 3.89	72.94 ± 2.65	86.90 ± 1.63	
Compound 4a	67.52 ± 3.31	75.00 ± 2.39	86.52 ± 5.31	
Compound 5a	75.15 ± 5.90	84.53 ± 2.36	106.17 ± 3.34	
Compound 6a	60.05 ± 5.39	76.41 ± 2.29	100.96 ± 1.07	
Compound 7a	54.89 ± 1.04	62.07 ± 4.16	79.65 ± 3.04	
Compound 8a	74.54 ± 1.42	81.49 ± 3.47	87.62 ± 2.98	
Compound 9a	63.56 ± 2.88	70.76 ± 3.98	81.08 ± 2.61	
Compound 10a	54.92 ± 1.34	64.71 ± 2.00	83.99 ± 4.36	
Compound 11a	61.54 ± 1.46	73.69 ± 3.89	97.27 ± 1.95	
Compound 12a	76.29 ± 3.14	81.94 ± 2.30	88.14 ± 2.09	
Compound 13a	71.47 ± 2.53	77.92 ± 3.02	90.25 ± 3.77	
Compound 14a	68.03 ± 3.05	77.10 ± 0.64	81.93 ± 1.35	
Compound 15a	62.21 ± 2.53	72.52 ± 2.02	89.22 ± 0.36	

Table S3. Protection effects of the curcumin derivatives **1-15** and their acetates **1a-15a** on Radiation.

Group	Cell viability			
	Dose (5 μM)	Dose (10 μM)	Dose (20 μM)	Dose (0 μM)
Control				
Radiation				50.22 ± 1.08
Curcumin	55.12 ± 1.37	59.39 ± 0.57	75.47 ± 0.68	

Compound 1	47.61 ± 1.20	57.30 ± 1.29	89.72 ± 8.17
Compound 2	50.96 ± 1.31	52.89 ± 2.05	54.68 ± 1.78
Compound 3	49.57 ± 2.70	51.85 ± 2.04	57.44 ± 1.80
Compound 4	49.46 ± 1.97	51.75 ± 0.40	59.56 ± 0.59
Compound 5	58.70 ± 4.93	64.93 ± 4.67	104.23 ± 2.86
Compound 6	58.25 ± 1.05	62.18 ± 1.03	83.08 ± 6.65
Compound 7	61.54 ± 2.47	63.26 ± 3.00	69.98 ± 4.31
Compound 8	53.58 ± 0.78	55.23 ± 1.26	78.50 ± 2.59
Compound 9	56.32 ± 1.77	59.94 ± 1.26	65.00 ± 1.84
Compound 10	58.43 ± 1.95	63.89 ± 2.22	79.31 ± 3.13
Compound 11	65.09 ± 0.40	84.55 ± 3.40	94.25 ± 4.35
Compound 12	56.58 ± 1.03	63.12 ± 1.52	76.81 ± 1.27
Compound 13	61.42 ± 1.58	67.83 ± 2.32	87.38 ± 0.60
Compound 14	53.08 ± 0.34	54.44 ± 0.72	63.52 ± 1.49
Compound 15	58.64 ± 0.61	63.45 ± 0.21	76.99 ± 1.23
Compound 1a	60.01 ± 5.39	62.85 ± 6.12	75.47 ± 0.68
Compound 2a	57.71 ± 4.12	61.56 ± 4.11	92.24 ± 6.04
Compound 3a	57.10 ± 3.05	64.19 ± 0.94	69.11 ± 2.51
Compound 4a	56.43 ± 3.51	61.48 ± 3.74	97.55 ± 3.44
Compound 5a	58.17 ± 1.86	68.65 ± 5.90	104.65 ± 5.01
Compound 6a	65.56 ± 2.17	71.70 ± 2.56	84.86 ± 6.57
Compound 7a	62.11 ± 1.39	65.49 ± 2.03	75.74 ± 2.05
Compound 8a	60.33 ± 1.38	62.24 ± 0.75	81.89 ± 2.94
Compound 9a	56.88 ± 1.03	60.92 ± 1.55	70.15 ± 2.99
Compound 10a	61.98 ± 2.45	67.94 ± 4.69	81.14 ± 3.72
Compound 11a	60.93 ± 1.64	64.92 ± 0.58	106.32 ± 7.58
Compound 12a	59.04 ± 1.00	63.19 ± 1.90	77.34 ± 2.18
Compound 13a	66.55 ± 2.11	75.50 ± 1.73	88.50 ± 1.18
Compound 14a	64.83 ± 0.66	68.68 ± 1.31	77.66 ± 1.75

Compound 15a	61.29 ± 1.82	65.00 ± 2.78	77.64 ± 1.03

Section 2: Structure data of curcumin derivatives **1-15** and their acetates **1a-15a**.

(1E,6E)-1-(3-((dimethylamino)methyl)-4-hydroxy-5-methoxyphenyl)-7-(3-ethoxy-4-hydroxyphenyl)hepta-1,6-diene-3,5-dione (**1**) ^{1}H NMR (600 MHz, CD_3OD) δ 7.57 (d, J = 15.6 Hz, 1H), 7.37 (s, 1H), 7.23 (d, J = 37.0 Hz, 2H), 7.12 (s, 2H), 7.03 (s, 1H), 6.83 (d, J = 8.1 Hz, 1H), 6.76 (s, 1H), 6.63 (d, J = 15.6 Hz, 1H), 5.33 (s, 1H), 4.32 (s, 2H), 4.15 (d, J = 6.9 Hz, 2H), 3.97 (s, 2H), 3.90 (s, 3H), 2.85 (s, 6H), 1.45 (t, J = 6.8 Hz, 3H). ^{13}C NMR (151 MHz, DMSO-d_6) δ 183.67, 183.62, 150.09, 148.29, 147.59, 141.22, 139.88, 126.79, 125.69, 123.51, 121.53, 116.26, 113.14, 110.93, 101.26, 64.39, 60.61, 56.21, 44.56, 40.53, 15.18. HRMS(ESI) m/z: calcd for $C_{25}H_{30}NO_6^+$ $[M+H]^+$: 440.2073, found: 440.2069. Yield: 22.56%.

(1E,6E)-1-(3-((dimethylamino)methyl)-4-hydroxy-5-methoxyphenyl)-7-(4-hydroxy-3,5-dimethoxyphenyl)hepta-1,6-diene-3,5-dione **(2)** ^{1}H NMR (600 MHz, DMSO-d_6) δ 7.55 (dd, J = 15.7, 10.8 Hz, 2H), 7.26 (s, 1H), 7.05 (s, 2H), 6.78 (dd, J = 31.9, 15.8 Hz, 2H), 6.08 (s, 1H), 3.83 (s, 9H), 3.63 (s, 2H), 2.30 (s, 2H), 2.27 (s, 6H). ^{13}C NMR (151 MHz, DMSO-d_6) δ 183.34, 182.96, 149.66, 148.12, 147.81, 140.98, 140.82, 138.41, 126.76, 125.20, 125.15, 122.90, 122.75, 121.46, 121.05, 110.51, 106.27, 100.79, 60.16, 59.83, 56.09, 55.73, 55.58, 44.07, 43.80. HRMS(ESI) m/z: calcd for $C_{25}H_{30}NO_7^+$ $[M+H]^+$: 456.2022, found: 456.2016. Yield: 38.46%.

(1E,6E)-1-(3-((dimethylamino)methyl)-4-hydroxy-5-methoxyphenyl)-7-(4-hydroxy-2-methylphenyl)hepta-1,6-diene-3,5-dione (**3**) ^{1}H NMR (600 MHz, CD_3OD) δ 7.84 (d, J = 15.6 Hz, 1H), 7.58 – 7.51 (m, 2H), 7.30 (d, J = 19.8 Hz, 2H), 7.08 (d, J = 38.5 Hz, 1H), 6.65 (d, J = 2.7 Hz, 2H), 6.53 (d, J = 15.6 Hz, 1H), 4.33 (d, J = 5.2 Hz, 2H), 3.95 (s, 3H), 3.90 (s, 2H), 2.88 (s, 7H), 2.34 (s, 3H). ^{13}C NMR (151 MHz, CD_3OD) δ 190.50, 159.42, 148.58, 140.84, 139.93, 137.38, 127.74, 125.26, 125.09, 123.51, 121.29, 120.39, 116.97, 113.52, 109.75, 59.95, 59.86, 55.08, 54.69, 42.89. HRMS(ESI) m/z: calcd for $C_{24}H_{28}NO_5^+$ $[M+H]^+$: 410.1967, found: 410.1962. Yield: 24.52%.

(1E,6E)-1-(3-((dimethylamino)methyl)-4-hydroxy-5-methoxyphenyl)-7-(2-fluoro-4-hydroxyphenyl)hepta-1,6-diene-3,5-dione (**4**) ^{1}H NMR (600 MHz, CD_3OD) δ 7.70 (t, J = 8.7 Hz, 1H), 7.65 – 7.36 (m, 4H), 6.83 (dd, J = 43.7, 15.9 Hz, 2H), 6.72 (dd, J = 20.3, 10.8 Hz, 2H),

4.26 (s, 2H), 3.92 (s, 3H), 2.73 (s, 6H), 2.51 (s, 2H). ^{13}C NMR (151 MHz, CD_3OD) δ 184.00, 182.34, 163.41, 161.74, 151.21, 148.56, 141.03, 132.49, 129.61, 129.58, 125.28, 123.69, 122.35, 120.54, 112.27, 109.89, 102.77, 59.89, 59.58, 55.08, 42.79. HRMS(ESI) m/z: calcd for $C_{23}H_{25}FNO_5^+$ [M+H]$^+$: 414.1716, found: 414.1714. Yield: 27.96%

(1E,6E)-1-(3,4-dimethoxyphenyl)-7-(3-((dimethylamino)methyl)-4-hydroxy-5-methoxyphenyl)hepta-1,6-diene-3,5-dione (**5**) ^{1}H NMR (600 MHz, CD_3OD) δ 7.54 (dd, *J* = 15.6, 5.3 Hz, 2H), 7.31 (s, 1H), 7.25 (s, 1H), 7.18 (s, 1H), 7.14 (d, J = 8.1 Hz, 1H), 6.93 (d, *J* = 8.2 Hz, 1H), 6.67 (dd, *J* = 38.5, 15.7 Hz, 2H), 4.33 (s, 2H), 3.96 (s, 3H), 3.87 (s, 3H), 3.84 (s, 3H), 2.88 (s, 6H), 2.85 (s, 2H). ^{13}C NMR (151 MHz, DMSO-d_6) δ 183.84, 183.45, 151.48, 149.50, 148.66, 141.03, 140.54, 128.03, 126.55, 126.20, 123.51, 122.73, 122.49, 117.79, 112.42, 112.15, 110.95, 101.62, 56.64, 56.10, 56.08, 42.33. HRMS(ESI) m/z: calcd for $C_{25}H_{30}NO_6^+$ [M+H]$^+$: 440.2073, found: 440.2068. Yield: 35.95%.

(1E,6E)-1-(3-((dimethylamino)methyl)-4-hydroxy-5-methoxyphenyl)-7-(2,3,4-trimethoxyphenyl)hepta-1,6-diene-3,5-dione (**6**) ^{1}H NMR (600 MHz, CD_3OD) δ 7.83 (d, *J* = 16.0 Hz, 1H), 7.58 (d, *J* = 15.8 Hz, 1H), 7.42 (d, *J* = 8.8 Hz, 1H), 7.37 (s, 1H), 7.27 (s, 1H), 6.84 (dd, *J* = 8.7, 4.9 Hz, 1H), 6.74 (t, *J* = 15.8 Hz, 2H), 4.35 (s, 2H), 3.97 (s, 3H), 3.91 (d, *J* = 2.2 Hz, 3H), 3.89 (s, 3H), 3.83 (s, 3H), 2.89 (s, 6H), 2.86 (d, *J* = 12.6 Hz, 2H). ^{13}C NMR (151 MHz, DMSO-d_6) δ 184.43, 182.88, 155.87, 153.11, 150.18, 148.31, 142.30, 141.59, 134.66, 125.67, 123.52, 123.42, 123.31, 121.57, 121.54, 110.93, 109.05, 101.71, 61.88, 60.93, 60.54, 56.52, 56.21, 44.54. HRMS(ESI) m/z: calcd for $C_{26}H_{32}NO_7^+$ [M+H]$^+$: 470.2178, found: 470.2173.Yield: 11.23%.

(1E,6E)-1-(3-chloro-4-hydroxyphenyl)-7-(3-((dimethylamino)methyl)-4-hydroxy-5-methoxyphenyl)hepta-1,6-diene-3,5-dione (**7**) ^{1}H NMR (600 MHz, DMSO-d_6) δ 11.13 (s, 1H), 10.78 (d, *J* = 50.4 Hz, 1H), 9.97 (d, *J* = 171.7 Hz, 1H), 7.79 (d, *J* = 4.8 Hz, 1H), 7.61 (s, 1H), 7.55 (dd, *J* = 16.1, 7.4 Hz, 3H), 7.47 (s, 1H), 7.18 (d, *J* = 8.4 Hz, 1H), 6.87 (dd, *J* = 29.7, 15.8 Hz, 2H), 4.31 (d, *J* = 35.9 Hz, 2H), 3.94 (d, *J* = 1.9 Hz, 3H), 2.74 (s, 6H), 2.73 – 2.69 (m, 2H). ^{13}C NMR (151 MHz, DMSO-d_6) δ 183.84, 183.45, 151.48, 149.50, 148.66, 141.03, 140.55, 128.03, 126.55, 126.21, 123.52, 122.73, 122.49, 117.79, 112.42, 112.15, 110.95, 101.62, 56.64, 56.10, 56.08, 54.51, 42.33. HRMS(ESI) m/z: calcd for $C_{23}H_{25}ClNO_5^+$ [M+H]$^+$: 430.1421, found: 430.1416. Yield:

13.89%.

(1E,6E)-1-(3-((dimethylamino)methyl)-4-hydroxy-5-methoxyphenyl)-7-(4-hydroxy-3,5-dimethylphenyl)hepta-1,6-diene-3,5-dione (**8**) ^{1}H NMR (600 MHz, CD_3OD) δ 7.52 (dd, *J* = 23.3, 15.8 Hz, 2H), 7.32 (s, 1H), 7.24 (s, 1H), 7.20 (s, 2H), 6.71 (d, *J* = 15.8 Hz, 1H), 6.56 (d, *J* = 15.8 Hz, 1H), 4.32 (s, 2H), 3.96 (s, 3H), 2.87 (s, 6H), 2.85 (s, 2H), 2.22 (s, 6H). ^{13}C NMR (151 MHz, CD_3OD) δ 185.99, 183.27, 157.33, 149.57, 149.34, 142.81, 140.51, 130.09, 128.80, 127.76, 126.15, 126.02, 123.68, 122.99, 121.76, 117.79, 112.73, 112.60, 57.83, 57.49, 56.79, 43.35, 16.64. HRMS(ESI) m/z: calcd for $C_{25}H_{30}NO_5^+$ [M+H]$^+$: 424.2118, found: 424.2120. Yield: 25.53%.

(1E,6E)-1-(3-((dimethylamino)methyl)-4-hydroxy-5-methoxyphenyl)-7-(4-hydroxy-2-methoxyphenyl)hepta-1,6-diene-3,5-dione (**9**) ^{1}H NMR (600 MHz, CD_3OD) δ 7.88 (d, *J* = 15.9 Hz, 1H), 7.54 (d, *J* = 15.7 Hz, 1H), 7.46 (d, *J* = 8.4 Hz, 1H), 7.33 (d, *J* = 22.5 Hz, 1H), 7.25 (s, 1H), 7.13 – 7.01 (m, 1H), 6.69 (dd, *J* = 46.0, 15.8 Hz, 2H), 6.47 – 6.36 (m, 2H), 4.33 (d, *J* = 12.9 Hz, 2H), 3.96 (s, 3H), 3.90 (s, 2H), 3.85 (d, *J* = 14.7 Hz, 3H), 2.87 (d, *J* = 18.2 Hz, 6H). ^{13}C NMR (151 MHz, DMSO-*d_6*) δ 191.17, 162.03, 160.28, 149.98, 148.30, 140.90, 135.74, 130.46, 125.78, 123.37, 123.07, 121.61, 120.99, 114.91, 110.85, 108.84, 101.42, 99.60, 60.37, 56.20, 55.98, 55.35, 44.45. HRMS(ESI) m/z: calcd for $C_{24}H_{28}NO_6^+$ [M+H]$^+$: 426.1916, found: 426.1911. Yield: 10.80%.

(1E,6E)-1-(3-((dimethylamino)methyl)-4-hydroxy-5-methoxyphenyl)-7-(4-hydroxyphenyl)hepta-1,6-diene-3,5-dione (**10**) ^{1}H NMR (600 MHz, CD_3OD) δ 7.61 – 7.53 (m, 2H), 7.46 (d, *J* = 8.5 Hz, 2H), 7.29 (d, *J* = 33.5 Hz, 2H), 6.82 (d, *J* = 8.5 Hz, 2H), 6.72 (d, *J* = 15.8 Hz, 1H), 6.58 (d, *J* = 15.8 Hz, 1H), 4.33 (s, 2H), 3.95 (s, 3H), 2.88 (s, 6H), 2.85 (s, 2H). ^{13}C NMR (151 MHz, CD_3OD) δ 185.67, 183.56, 161.15, 149.56, 149.37, 142.21, 140.69, 131.25, 128.73, 127.87, 126.19, 123.67, 123.01, 122.02, 117.80, 116.92, 112.78, 104.14, 57.77, 57.42, 56.82, 56.61, 43.35. HRMS(ESI) m/z: calcd for $C_{23}H_{26}NO_5^+$ [M+H]$^+$: 396.1811, found: 396.1808. Yield:10.59%.

(1E,6E)-1-(3-((dimethylamino)methyl)-4-hydroxy-5-methoxyphenyl)-7-(3-hydroxy-4-methoxyphenyl)hepta-1,6-diene-3,5-dione (**11**) ^{1}H NMR (600 MHz, CD_3OD) δ 7.55 (dd, *J* = 25.6, 15.8 Hz, 2H), 7.29 (s, 1H), 7.16 – 7.05 (m, 3H), 6.95 (d, *J* = 8.3 Hz, 1H), 6.67 (d, *J* = 15.8 Hz, 1H), 6.58 (d, *J* = 15.8 Hz, 1H), 4.10 (s, 2H), 3.94 (s, 3H), 3.90 (s, 3H), 2.69 (s, 6H). ^{13}C NMR (151 MHz, CD_3OD) δ 190.61, 149.99, 149.68, 148.45, 146.63, 140.54, 140.18, 128.30, 126.33, 124.30,

121.49, 118.65, 113.39, 111.18, 110.63, 57.81, 55.26, 55.02, 42.35. HRMS(ESI) m/z: calcd for $C_{24}H_{28}NO_6^+$ [M+H]$^+$: 426.1916, found: 426.1911. Yield:15.63%.

(1E,6E)-1-(3-((dimethylamino)methyl)-4-hydroxy-5-methoxyphenyl)-7-(4-hydroxy-2,5-dimethylphenyl)hepta-1,6-diene-3,5-dione (**12**) ^{1}H NMR (600 MHz, DMSO- *d*$_6$) *δ* 9.81 (s, 1H), 7.78 (d, *J* = 9.5 Hz, 1H), 7.60 – 7.40 (m, 4H), 6.86 (d, *J* = 15.6 Hz, 1H), 6.80 – 6.61 (m, 2H), 4.32 (s, 2H), 3.93 (d, *J* = 5.3 Hz, 3H), 2.72 (s, 6H), 2.51 (s, 3H), 2.32 (s, 2H), 2.12 (s, 3H). ^{13}C NMR (151 MHz, CD_3OD) *δ* 183.80, 182.88, 157.68, 150.71, 148.52, 140.48, 137.75, 137.39, 128.61, 125.62, 124.76, 123.65, 122.75, 120.83, 120.65, 116.20, 113.28, 109.92, 59.33, 55.12, 53.40, 42.75, 18.01, 14.43. HRMS(ESI) m/z: calcd for $C_{25}H_{30}NO_5^+$ [M+H]$^+$: 424.2124, found: 424.2118. Yield: 36.71%

(1E,6E)-1-(3-((dimethylamino)methyl)-4-hydroxy-5-methoxyphenyl)-7-(4-hydroxy-2,6-dimethylphenyl)hepta-1,6-diene-3,5-dione (**13**) ^{1}H NMR (600 MHz, CD_3OD) *δ* 9.59 (s, 1H), 7.84 (d, *J* = 16.2 Hz, 1H), 7.60 (d, *J* = 15.8 Hz, 1H), 7.35 (dd, *J* = 5.5, 1.9 Hz, 2H), 7.20 (d, *J* = 1.8 Hz, 1H), 7.05 (d, *J* = 1.6 Hz, 1H), 6.64 (d, *J* = 15.8 Hz, 1H), 6.56 (s, 1H), 6.33 (d, *J* = 16.2 Hz, 1H), 4.04 (s, 2H), 3.92 (s, 2H), 3.89 (s, 3H), 2.65 (s, 6H), 2.45 (s, 3H), 2.37 (s, 3H). ^{13}C NMR (151 MHz, CD_3OD) *δ* 190.52, 162.00, 161.80, 150.42, 148.45, 139.76, 132.56, 125.78, 123.30, 122.26, 121.13, 120.80, 109.84, 104.80, 91.66, 59.54, 55.11, 54.78, 42.83. HRMS(ESI) m/z: calcd for $C_{25}H_{30}NO_5^+$ [M+H]$^+$: 424.2124, found: 424.2119. Yield: 23.05%

(1E,6E)-1-(3-((dimethylamino)methyl)-4-hydroxy-5-methoxyphenyl)-7-(4-hydroxy-2,6-dimethoxyphenyl)hepta-1,6-diene-3,5-dione (**14**) ^{1}H NMR (600 MHz, CD_3OD) *δ* 9.59 (s, 1H), 8.08 (d, *J* = 16.0 Hz, 1H), 7.53 (d, *J* = 15.7 Hz, 1H), 7.39 – 7.30 (m, 1H), 7.20 (d, *J* = 1.4 Hz, 1H), 7.04 (s, 1H), 6.99 (d, *J* = 16.0 Hz, 1H), 6.62 (d, *J* = 15.7 Hz, 1H), 6.14 (s, 2H), 3.93 (s, 3H), 3.89 (s, 6H), 3.84 (s, 2H), 2.65 (s, 2H), 2.48 (s, 6H). ^{13}C NMR (151 MHz, CD_3OD) *δ* 190.52, 162.00, 161.80, 150.42, 148.45, 139.76, 132.56, 125.78, 123.30, 122.26, 121.13, 120.80, 109.84, 104.80, 91.66, 59.54, 55.11, 54.78, 42.83. HRMS(ESI) m/z: calcd for $C_{25}H_{30}NO_7^+$ [M+H]$^+$: 456.2022, found: 456.2016. Yield: 20.9%.

(1E,6E)-1-(3-((dimethylamino)methyl)-4-hydroxy-5-methoxyphenyl)-7-(4-hydroxy-3-methoxyphenyl)hepta-1,6-diene-3,5-dione (**15**) ^{1}H NMR (600 MHz, DMSO-*d*$_6$) *δ* 7.57 – 7.53

(m, 1H), 7.32 (s, 1H), 7.26 (d, *J* = 1.4 Hz, 1H), 7.15 (dd, *J* = 8.2, 1.7 Hz, 1H), 7.07 (s, 1H), 6.83 (d, *J* = 8.1 Hz, 1H), 6.75 (d, *J* = 15.8 Hz, 2H), 6.06 (s, 1H), 3.84 (d, *J* = 7.4 Hz, 6H), 3.63 (s, 2H), 2.30 (s, 2H), 2.26 (s, 6H). ^{13}C NMR (151 MHz, DMSO-d_6) δ 191.18, 150.02, 149.82, 148.46, 148.32, 141.19, 141.15, 126.79, 125.75, 123.59, 123.52, 121.58, 116.17, 111.81, 111.04, 101.31, 60.12, 56.24, 56.16, 55.35, 44.36. HRMS(ESI) m/z: calcd for $C_{24}H_{28}NO_6^+$ [M+H]$^+$: 426.1916, found: 426.1914. Yield: 26.21%.

(1E,6E)-1-(3-((dimethylamino)methyl)-4-hydroxy-5-methoxyphenyl)-7-(3-ethoxy-4-hydroxyphenyl)hepta-1,6-diene-3,5-dione acetate (**1a**) ^1H NMR (600 MHz, CD_3OD) δ 9.64 (s, 1H), 7.53 (d, *J* = 6.7 Hz, 2H), 7.41 (d, *J* = 5.2 Hz, 2H), 7.17 (dd, *J* = 63.4, 52.3 Hz, 3H), 6.84 (t, *J* = 15.8 Hz, 1H), 6.59 (d, *J* = 15.1 Hz, 1H), 4.18 – 4.10 (m, 5H), 3.89 (s, 3H), 2.74 (s, 3H), 2.72 (s, 3H), 1.94 (s, 3H). HRMS(ESI) m/z: calcd for $C_{25}H_{30}NO_6^+$ [M+H]$^+$: 440.2073, found: 440.2064. Yield: 94.82%.

(1E,6E)-1-(3-((dimethylamino)methyl)-4-hydroxy-5-methoxyphenyl)-7-(4-hydroxy-3,5-dimethoxyphenyl)hepta-1,6-diene-3,5-dione acetate **(2a)** ^1H NMR (600 MHz, CD_3OD) δ 7.53 (dd, *J* = 15.4, 7.0 Hz, 2H), 7.39 (s, 1H), 7.25 (s, 1H), 7.10 (s, 1H), 6.90 (s, 2H), 6.62 (d, *J* = 15.3 Hz, 2H), 4.10 (s, 2H), 3.93 (s, 3H), 3.88 (d, *J* = 3.7 Hz, 6H), 2.70 (s, 6H), 1.94 (s, 3H). HRMS(ESI) m/z: calcd for $C_{25}H_{30}NO_7^+$ [M-CH_3COOH+H]$^+$: 456.2022, found: 456.2018. Yield: 93.87%.

(1E,6E)-1-(3-((dimethylamino)methyl)-4-hydroxy-5-methoxyphenyl)-7-(4-hydroxy-2-methylphenyl)hepta-1,6-diene-3,5-dione acetate (**3a**) ^1H NMR (600 MHz, CD_3OD) δ 7.88 (d, *J* = 15.6 Hz, 1H), 7.56 (t, *J* = 11.1 Hz, 2H), 7.40 (s, 1H), 7.29 (s, 1H), 7.15 (s, 1H), 6.66 (d, *J* = 8.0 Hz, 3H), 6.55 (d, *J* = 15.5 Hz, 1H), 4.14 (s, 2H), 3.94 (s, 3H), 2.72 (s, 6H), 2.38 (s, 3H), 1.94 (s, 3H). HRMS(ESI) m/z: calcd for $C_{24}H_{28}NO_5^+$ [M-CH_3COOH+H]$^+$: 410.1967, found: 410.1964. Yield: 86.21%.

(1E,6E)-1-(3-((dimethylamino)methyl)-4-hydroxy-5-methoxyphenyl)-7-(2-fluoro-4-hydroxyphenyl)hepta-1,6-diene-3,5-dione acetate (**4a**) ^1H NMR (600 MHz, CD_3OD) δ 7.65 (d, *J* = 15.8 Hz, 1H), 7.58 – 7.49 (m, 2H), 7.28 (s, 1H), 7.17 (s, 1H), 6.65 (d, *J* = 7.8 Hz, 3H), 6.60 –

6.52 (m, 1H), 4.93 – 4.91 (m, 2H), 4.19 (s, 2H), 3.94 (s, 3H), 2.77 (s, 6H), 1.95 (s, 3H). HRMS(ESI) m/z: calcd for $C_{23}H_{25}FNO_5^+$ [M-CH_3COOH+H]$^+$: 414.1716, found: 414.1713. Yield: 92.53%.

(1E,6E)-1-(3,4-dimethoxyphenyl)-7-(3-((dimethylamino)methyl)-4-hydroxy-5-methoxyphenyl)hepta-1,6-diene-3,5-dione acetate (**5a**) ^{1}H NMR (600 MHz, CD_3OD) δ 9.68 (s, 1H), 7.54 (s, 1H), 7.45 (d, *J* = 13.9 Hz, 1H), 7.35 – 7.03 (m, 3H), 6.94 (d, *J* = 6.8 Hz, 1H), 6.65 (s, 1H), 4.21 (s, 2H), 4.03 – 3.74 (m, 9H), 2.78 (s, 2H), 2.75 (s, 6H), 1.95 (s, 3H). HRMS(ESI) m/z: calcd for $C_{25}H_{30}NO_6^+$ [M-CH_3COOH+H]$^+$: 440.2073, found: 440.2069. Yield: 91.34%.

(1E,6E)-1-(3-((dimethylamino)methyl)-4-hydroxy-5-methoxyphenyl)-7-(2,3,4-trimethoxyphenyl)hepta-1,6-diene-3,5-dione acetate (**6a**) ^{1}H NMR (600 MHz, CD_3OD) δ 7.80 (d, J = 15.6 Hz, 1H), 7.54 (d, *J* = 14.8 Hz, 1H), 7.37 (s, 1H), 7.24 (s, 1H), 7.11 (s, 1H), 6.80 (d, J = 7.4 Hz, 1H), 6.66 (d, *J* = 19.3 Hz, 2H), 4.05 (s, 2H), 3.87 (dd, *J* = 36.0, 22.2 Hz, 12H), 2.76 – 2.52 (m, 8H), 1.93 (s, 3H). HRMS(ESI) m/z: calcd for $C_{26}H_{32}NO_7^+$ [M-CH_3COOH+H]$^+$: 470.2178, found: 470.2174.Yield: 89.90%.

(1E,6E)-1-(3-chloro-4-hydroxyphenyl)-7-(3-((dimethylamino)methyl)-4-hydroxy-5-methoxyphenyl)hepta-1,6-diene-3,5-dione acetate (**7a**) ^{1}H NMR (600 MHz, CD_3OD) δ 9.67 (s, 1H), 7.64 – 7.28 (m, 5H), 7.17 (s, 1H), 6.93 (d, *J* = 8.0 Hz, 1H), 6.78 – 6.51 (m, 2H), 4.19 (d, *J* = 8.8 Hz, 2H), 3.95 (s, 3H), 3.91 (s, 2H), 2.75 (d, *J* = 16.8 Hz, 6H), 1.95 (s, 3H). HRMS(ESI) m/z: calcd for $C_{23}H_{25}ClNO_5^+$ [M-CH_3COOH+H]$^+$: 430.1421, found: 430.1415. Yield: 94.21%.

(1E,6E)-1-(3-((dimethylamino)methyl)-4-hydroxy-5-methoxyphenyl)-7-(4-hydroxy-3,5-dimethylphenyl)hepta-1,6-diene-3,5-dione acetate (**8a**) ^{1}H NMR (600 MHz, CD_3OD) δ 7.51 (t, *J* = 16.8 Hz, 2H), 7.27 (s, 1H), 7.20 (s, 2H), 7.16 (s, 1H), 6.60 (dd, *J* = 57.3, 15.2 Hz, 2H), 4.18 (s, 2H), 3.93 (s, 3H), 2.74 (d, *J* = 14.2 Hz, 8H), 2.22 (s, 6H), 1.95 (s, 3H). HRMS(ESI) m/z: calcd for $C_{25}H_{30}NO_5^+$ [M-CH_3COOH+H]$^+$: 424.2118, found: 424.2120. Yield: 26.21%.

(1E,6E)-1-(3-((dimethylamino)methyl)-4-hydroxy-5-methoxyphenyl)-7-(4-hydroxy-2-methoxyphenyl)hepta-1,6-diene-3,5-dione acetate (**9a**) ^{1}H NMR (600 MHz, CD_3OD) δ 7.86 (d, *J* = 15.8 Hz, 1H), 7.50 (d, *J* = 15.5 Hz, 1H), 7.44 (d, *J* = 8.4 Hz, 1H), 7.26 (s, 1H), 7.15 (s, 1H), 6.61

(d, *J* = 15.3 Hz, 2H), 6.43 (dd, *J* = 11.5, 5.1 Hz, 2H), 4.19 (s, 2H), 3.93 (s, 3H), 3.85 (s, 3H), 2.80 – 2.73 (m, 8H), 1.95 (s, 3H). HRMS(ESI) m/z: calcd for $C_{24}H_{28}NO_6^+$ [M-CH_3COOH+H]$^+$: 426.1916, found: 426.1912. Yield: 95.10%.

(1E,6E)-1-(3-((dimethylamino)methyl)-4-hydroxy-5-methoxyphenyl)-7-(4-hydroxyphenyl)hepta-1,6-diene-3,5-dione acetate (**10a**) ^{1}H NMR (600 MHz, CD_3OD) δ 7.53 (dd, *J* = 15.3, 9.9 Hz, 2H), 7.46 (dd, *J* = 10.5, 5.2 Hz, 2H), 7.27 (s, 1H), 7.17 (s, 1H), 6.81 (d, *J* = 8.3 Hz, 2H), 6.60 (dd, *J* = 50.0, 15.2 Hz, 2H), 4.21 (d, *J* = 3.3 Hz, 2H), 3.93 (s, 3H), 2.76 (d, *J* = 13.3 Hz, 8H), 1.96 (s, 3H). HRMS(ESI) m/z: calcd for $C_{23}H_{26}NO_5^+$ [M-CH_3COOH+H]$^+$: 396.1811, found: 396.1808. Yield: 96.62%.

(1E,6E)-1-(3-((dimethylamino)methyl)-4-hydroxy-5-methoxyphenyl)-7-(3-hydroxy-4-methoxyphenyl)hepta-1,6-diene-3,5-dione acetate (**11a**) ^{1}H NMR (600 MHz, CD_3OD) δ 7.48 (dd, *J* = 26.3, 15.7 Hz, 2H), 7.24 (s, 1H), 7.16 (s, 1H), 7.06 (s, 1H), 7.01 (d, *J* = 7.7 Hz, 1H), 6.88 (d, *J* = 8.2 Hz, 1H), 6.61 (d, *J* = 15.5 Hz, 1H), 6.50 (d, *J* = 15.7 Hz, 1H), 4.21 (d, *J* = 12.9 Hz, 2H), 3.92 (s, 2H), 3.85 (s, 3H), 2.78 (s, 6H), 1.96 (s, 3H). HRMS(ESI) m/z: calcd for $C_{24}H_{28}NO_6^+$ [M-CH_3COOH+H]$^+$: 426.1916, found: 426.1913. Yield: 98.26%.

(1E,6E)-1-(3-((dimethylamino)methyl)-4-hydroxy-5-methoxyphenyl)-7-(4-hydroxy-2,5-dimethylphenyl)hepta-1,6-diene-3,5-dione acetate (**12a**) ^{1}H NMR (600 MHz, DMSO-d_6) δ 9.81 (s, 1H), 7.78 (d, *J* = 9.5 Hz, 1H), 7.60 – 7.40 (m, 4H), 6.86 (d, *J* = 15.6 Hz, 1H), 6.80 – 6.61 (m, 2H), 4.32 (s, 2H), 3.93 (d, *J* = 5.3 Hz, 3H), 2.72 (s, 6H), 2.51 (s, 3H), 2.32 (s, 2H), 2.12 (s, 3H). HRMS(ESI) m/z: calcd for $C_{25}H_{30}NO_5^+$ [M-CH_3COOH+H]$^+$: 424.2124, found: 424.2118. Yield: 89.67%.

(1E,6E)-1-(3-((dimethylamino)methyl)-4-hydroxy-5-methoxyphenyl)-7-(4-hydroxy-2,6-dimethylphenyl)hepta-1,6-diene-3,5-dione acetate (**13a**) ^{1}H NMR (600 MHz, CD_3OD) δ 9.69 (s, 1H), 7.80 (d, *J* = 16.1 Hz, 1H), 7.47 (dd, *J* = 27.8, 1.5 Hz, 3H), 7.25 (d, *J* = 51.8 Hz, 1H), 6.76 – 6.38 (m, 2H), 6.28 (d, *J* = 16.1 Hz, 1H), 4.24 (s, 2H), 3.91 (s, 3H), 2.80 (s, 2H), 2.78 (s, 6H), 2.32 (s, 3H), 1.95 (s, 6H). HRMS(ESI) m/z: calcd for $C_{25}H_{30}NO_5^+$ [M-CH_3COOH+H]$^+$: 424.2124, found: 424.2120. Yield: 97.65%.

(1E,6E)-1-(3-((dimethylamino)methyl)-4-hydroxy-5-methoxyphenyl)-7-(4-hydroxy-2,6-dimethoxyphenyl)hepta-1,6-diene-3,5-dione acetate (**14a**) ^{1}H NMR (600 MHz, CD_3OD) δ 8.04 (d, J = 15.9 Hz, 1H), 7.51 – 7.45 (m, 1H), 7.27 (s, 1H), 7.15 (s, 1H), 6.95 (d, J = 15.9 Hz, 1H), 6.63 (d, J = 15.2 Hz, 1H), 6.11 (s, 2H), 4.21 (d, J = 3.2 Hz, 2H), 3.94 (s, 3H), 3.83 (d, J = 24.1 Hz, 6H), 2.77 (d, J = 18.8 Hz, 8H), 1.96 (s, 3H). HRMS(ESI) m/z: calcd for $C_{25}H_{30}NO_7^+$ [M-CH_3COOH+H]$^+$: 456.2022, found: 456.2018. Yield: 96.21%.

(1E,6E)-1-(3-((dimethylamino)methyl)-4-hydroxy-5-methoxyphenyl)-7-(4-hydroxy-3-methoxyphenyl)hepta-1,6-diene-3,5-dione acetate (**15a**) ^{1}H NMR (600 MHz, CD_3OD) δ 7.56 – 7.43 (m, 2H), 7.22 (s, 1H), 7.13 (s, 2H), 7.04 (d, J = 5.6 Hz, 1H), 6.80 (d, J = 7.8 Hz, 1H), 6.57 (t, J = 16.9 Hz, 2H), 4.18 (s, 2H), 3.88 (d, J = 13.5 Hz, 6H), 2.76 (d, J = 11.0 Hz, 6H), 1.96 (s, 3H). HRMS(ESI) m/z: calcd for $C_{24}H_{28}NO_6^+$ [M-CH_3COOH+H]$^+$: 426.1916, found: 426.1912. Yield: 96.53%.

Section 3: NMR spectrums and HRMS of curcumin derivatives **1-15** and their acetates **1a-15a**.

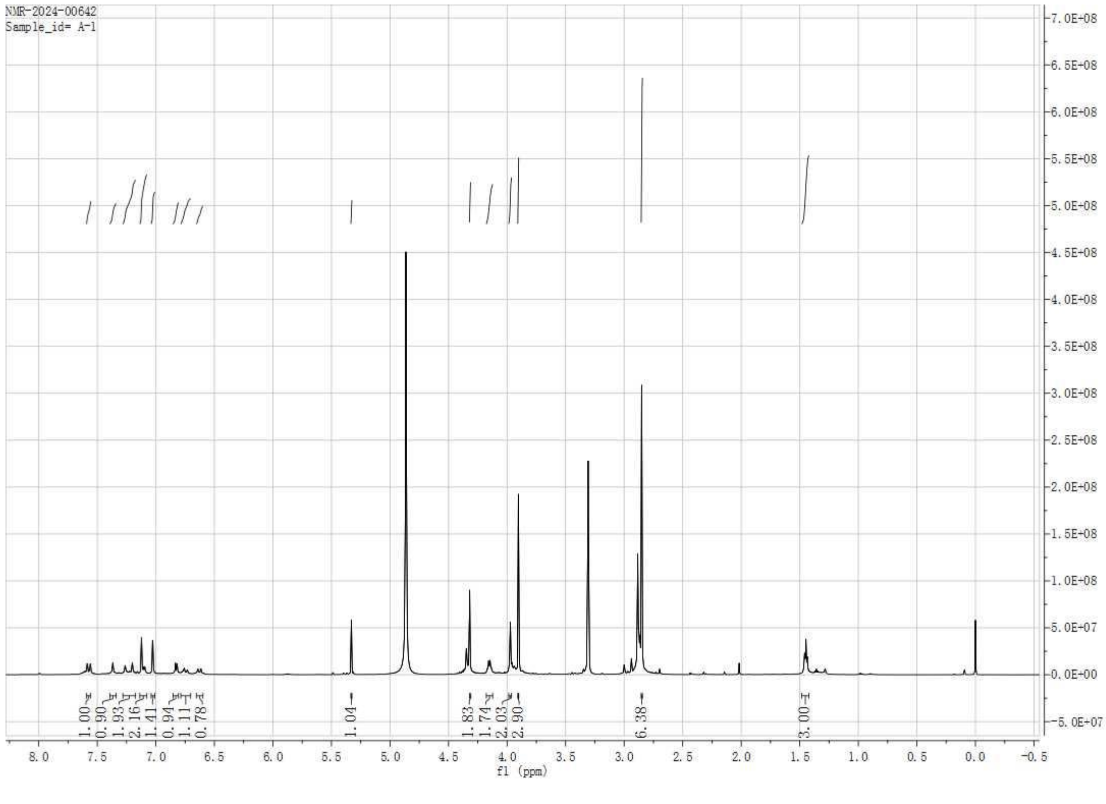

Supplementary Figure S1. ^{1}H-NMR spectrum of **1** in CD_3OD.

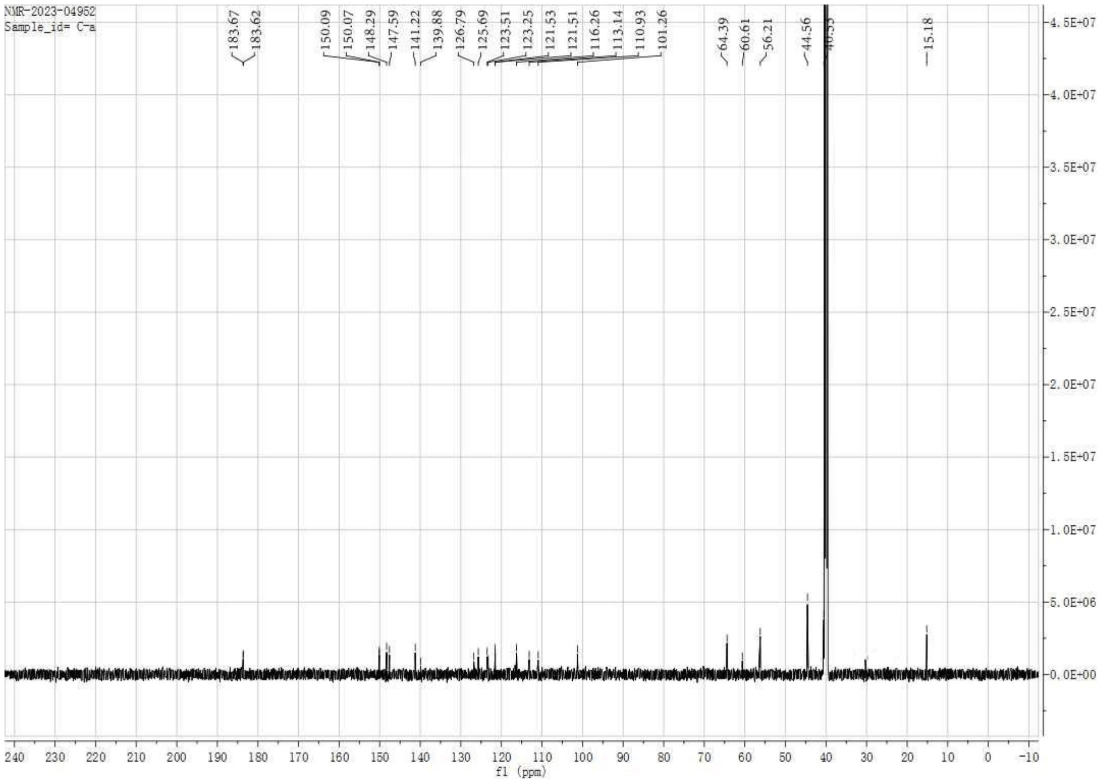

Supplementary Figure S2. ^{13}C-NMR spectrum of **1** in DMSO-d_6.

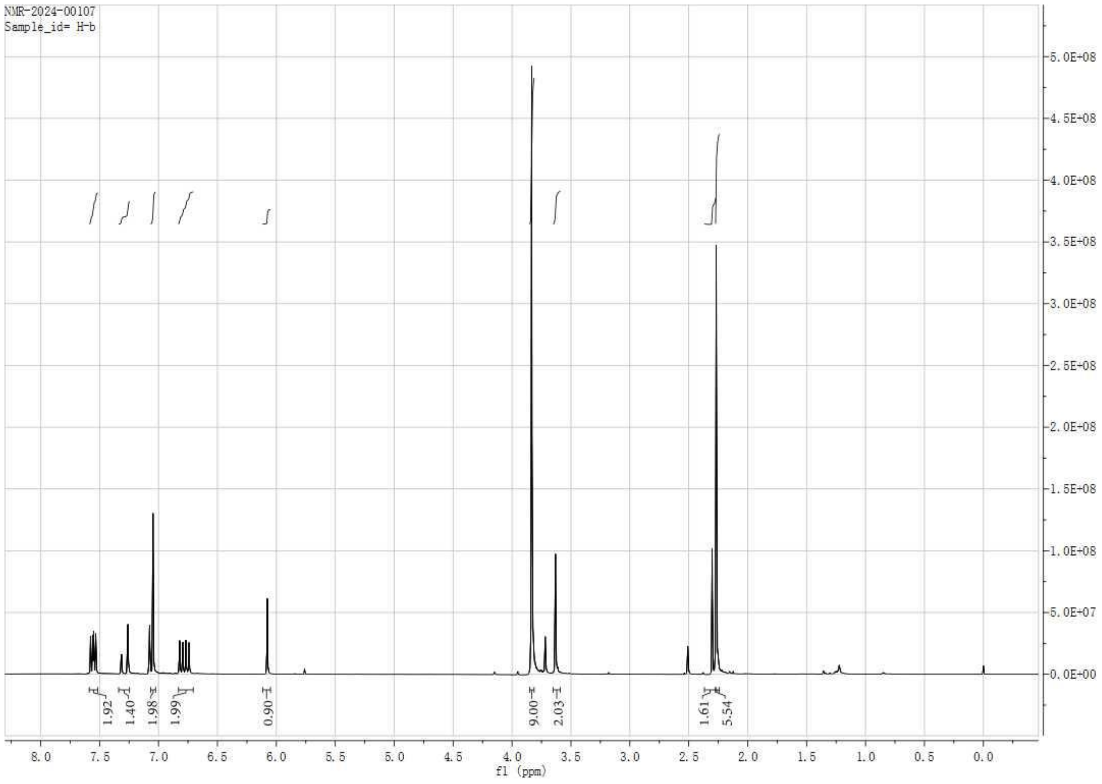

Supplementary Figure S3. ^{1}H-NMR spectrum of **2** in DMSO-d_6.

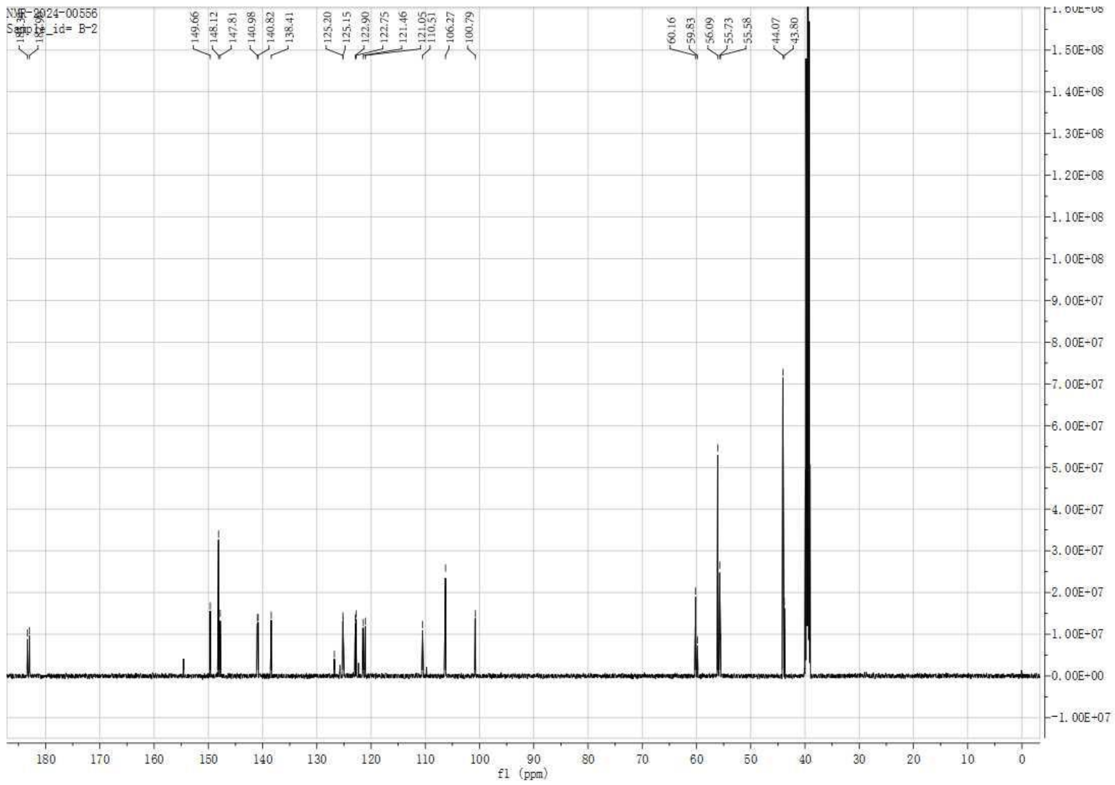

Supplementary Figure S4. ^{13}C-NMR spectrum of **2** in DMSO-d_6.

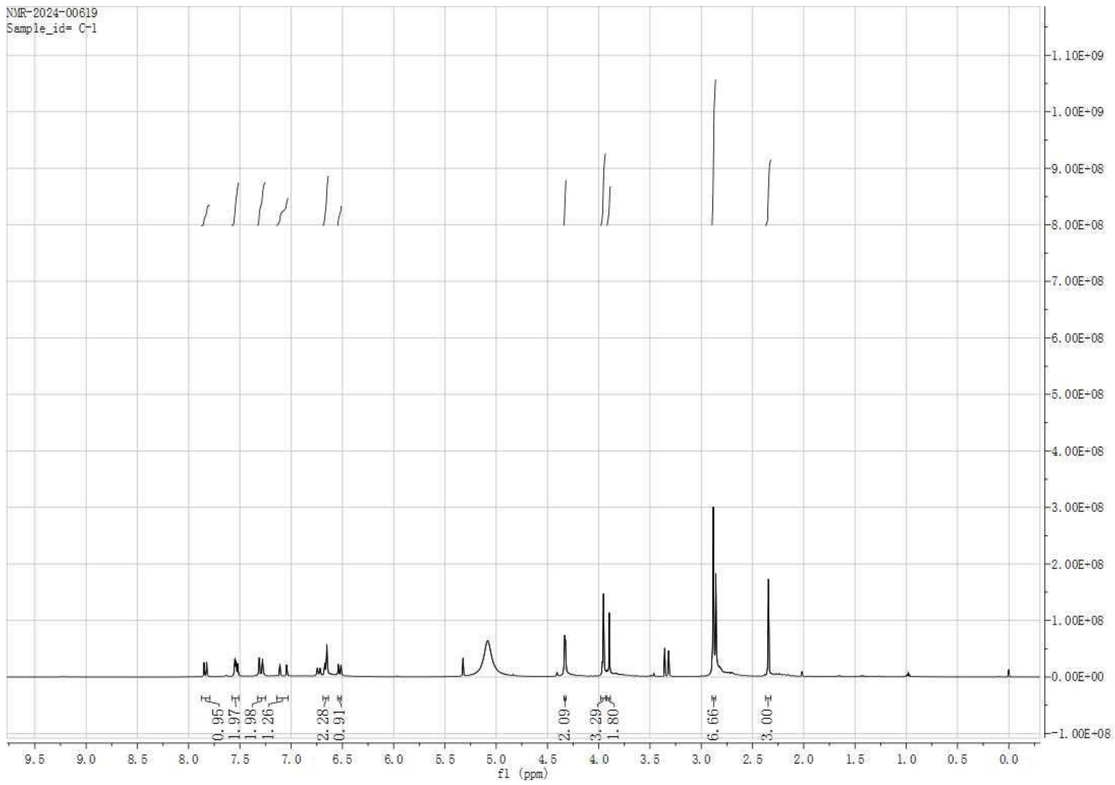

Supplementary Figure S5. ^{1}H-NMR spectrum of **3** in CD_3OD.

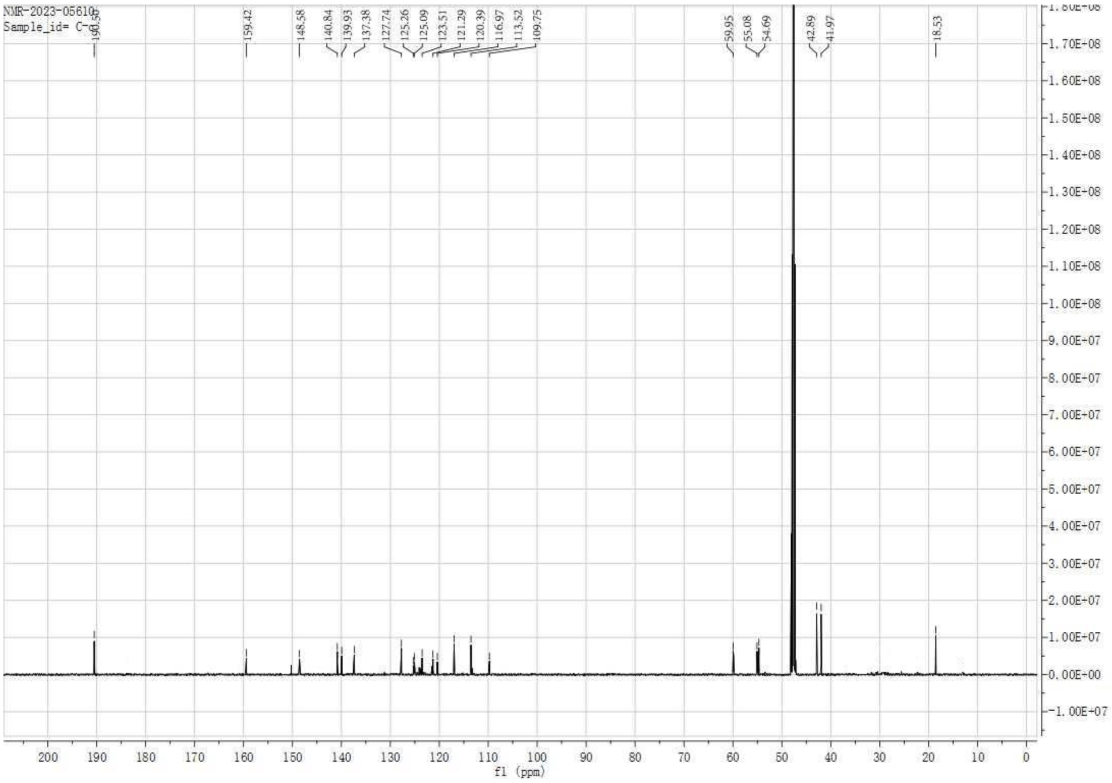

Supplementary Figure S6. ^{13}C-NMR spectrum of **3** in CD_3OD.

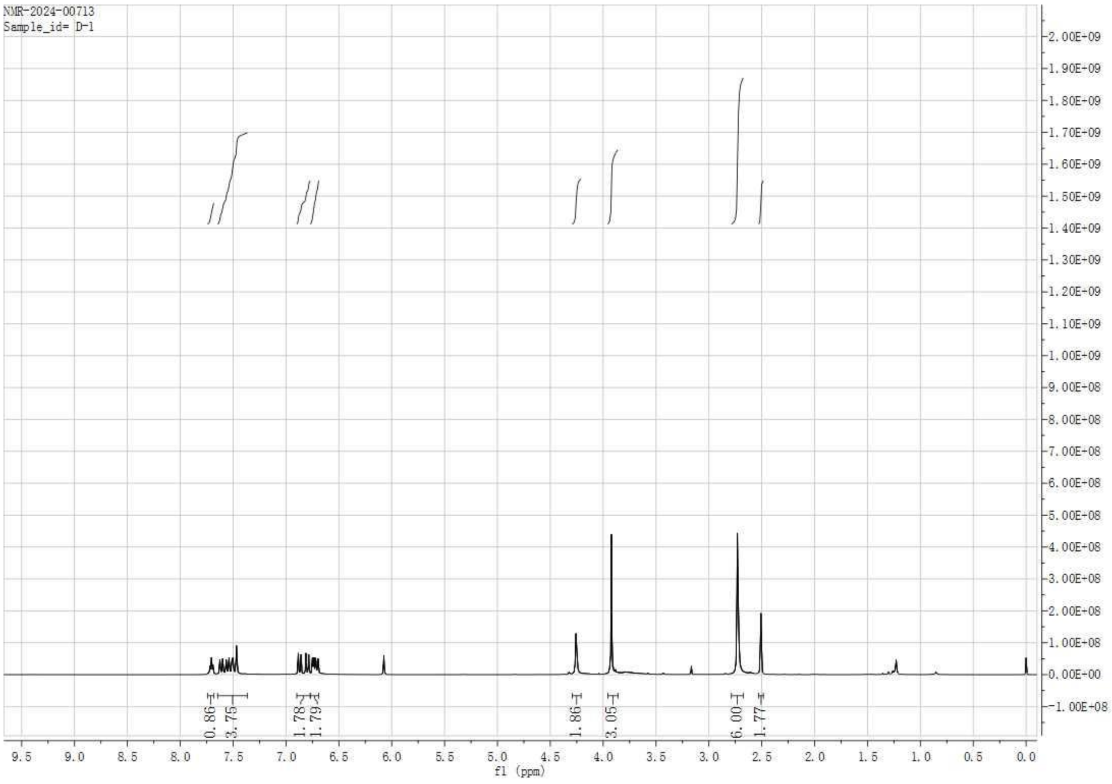

Supplementary Figure S7. ^{1}H-NMR spectrum of **4** in CD_3OD.

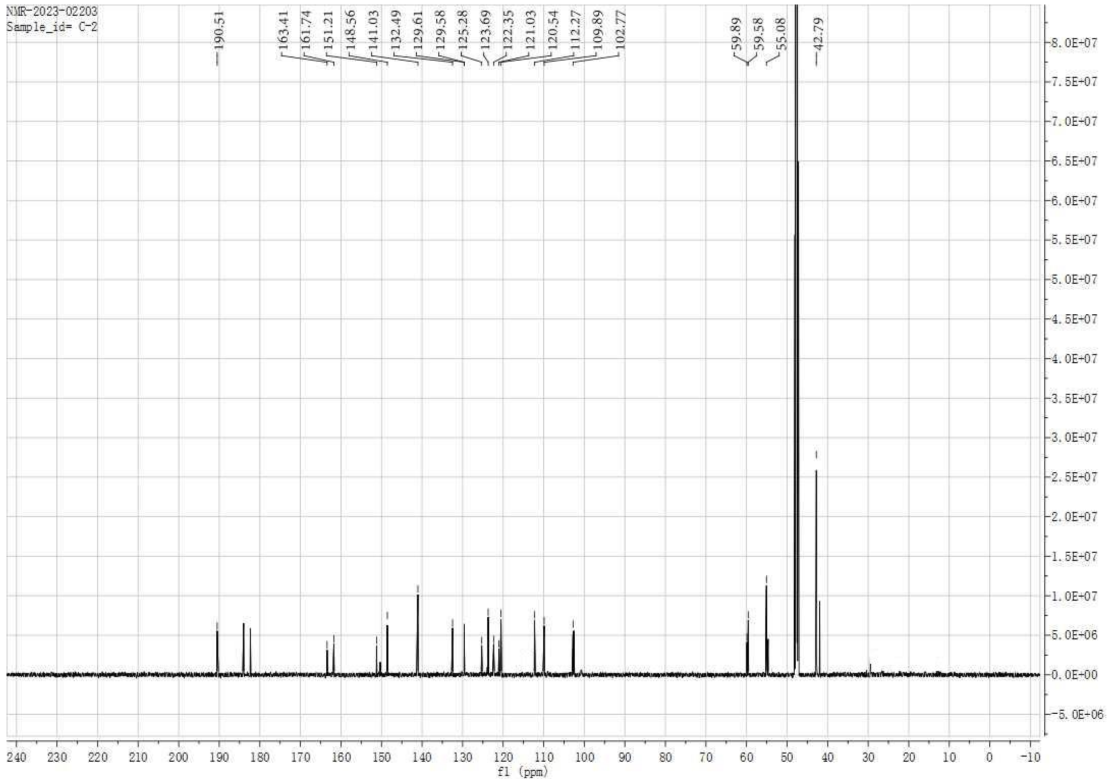

Supplementary Figure S8. ^{13}C-NMR spectrum of **4** in CD_3OD.

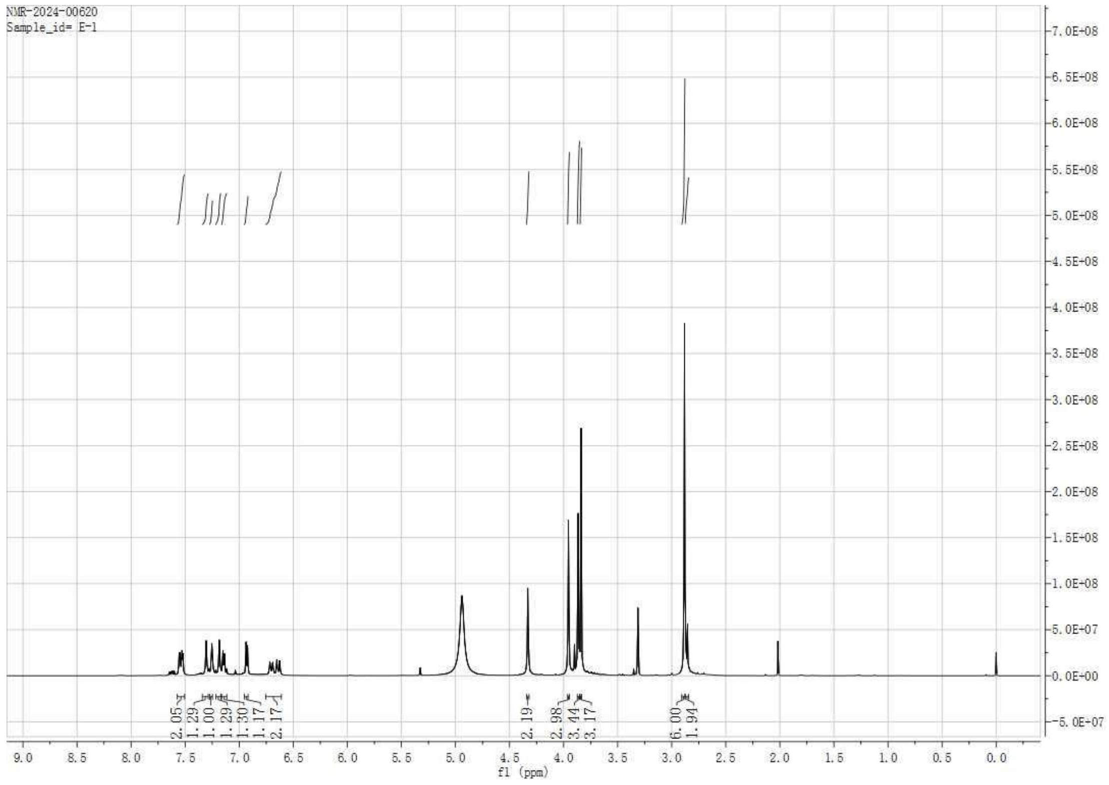

Supplementary Figure S9. ^{1}H-NMR spectrum of **5** in CD_3OD.

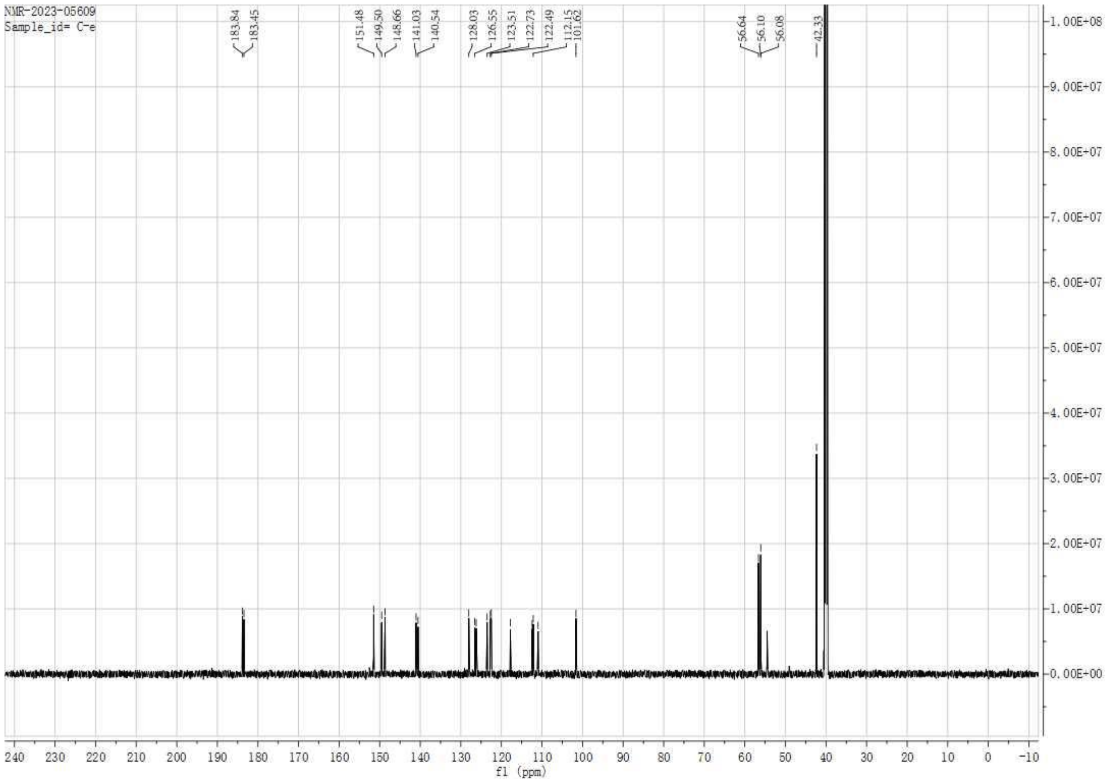

Supplementary Figure S10. ^{13}C-NMR spectrum of **5** in DMSO-d_6.

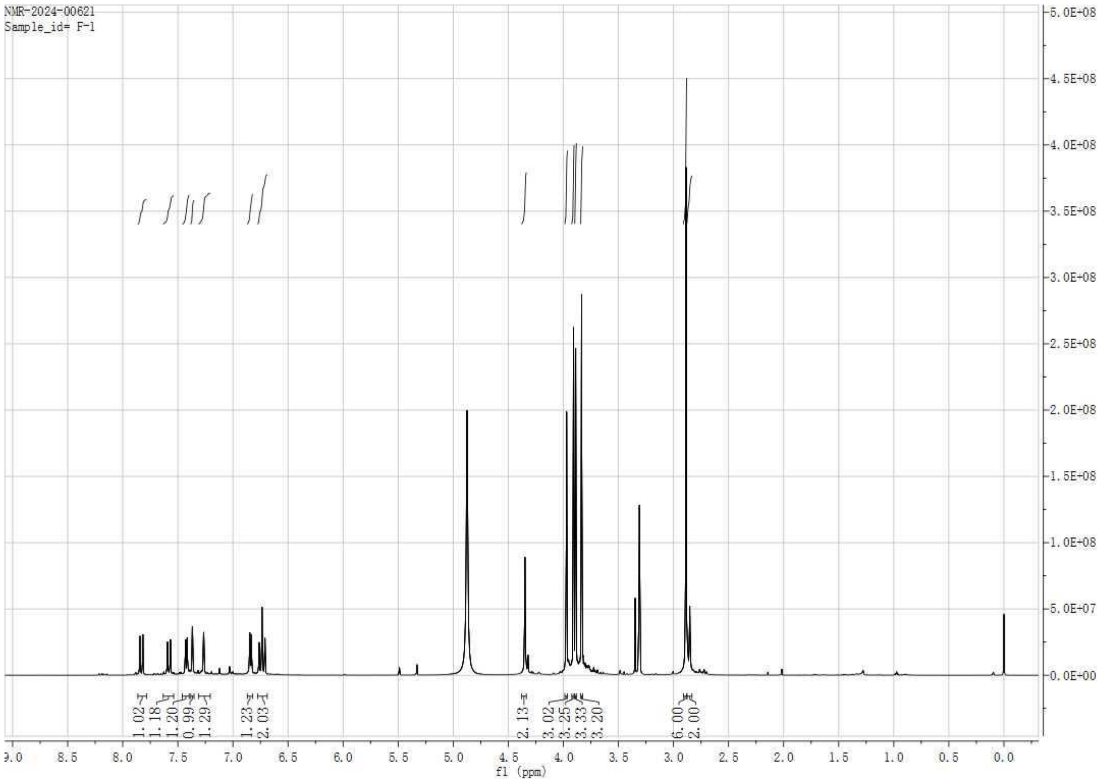

Supplementary Figure S11. ^{1}H-NMR spectrum of **6** in CD_3OD.

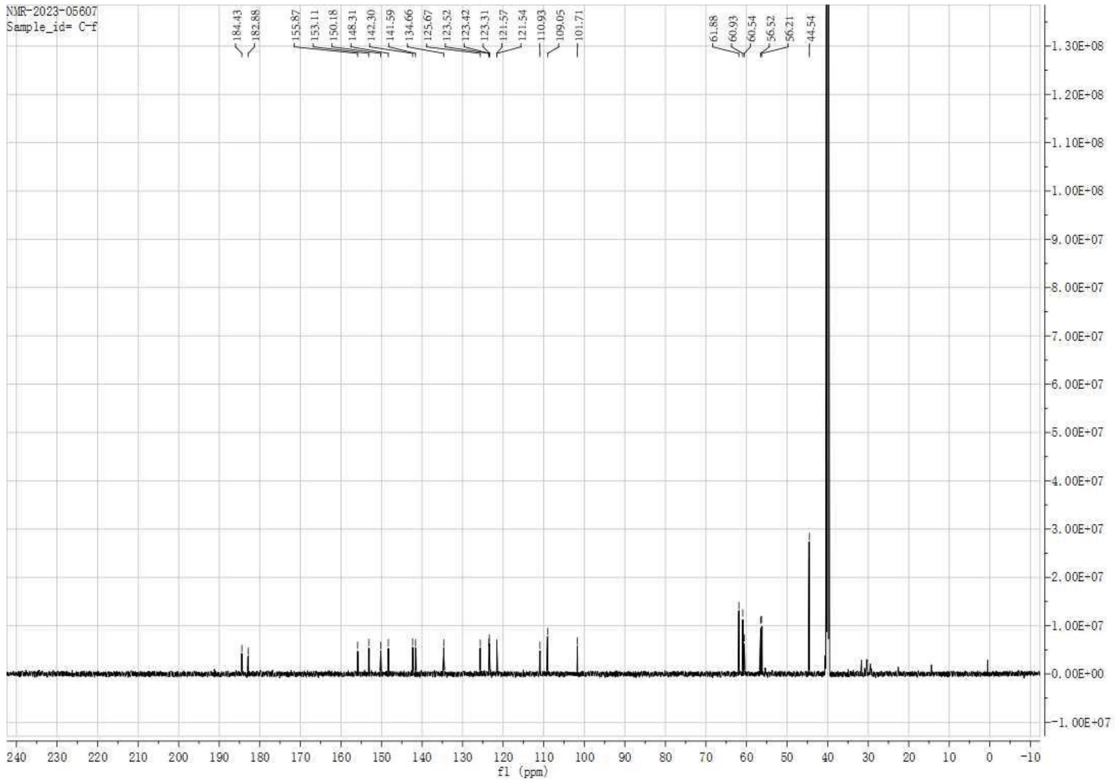

Supplementary Figure S12. ^{13}C-NMR spectrum of **6** in DMSO-d_6.

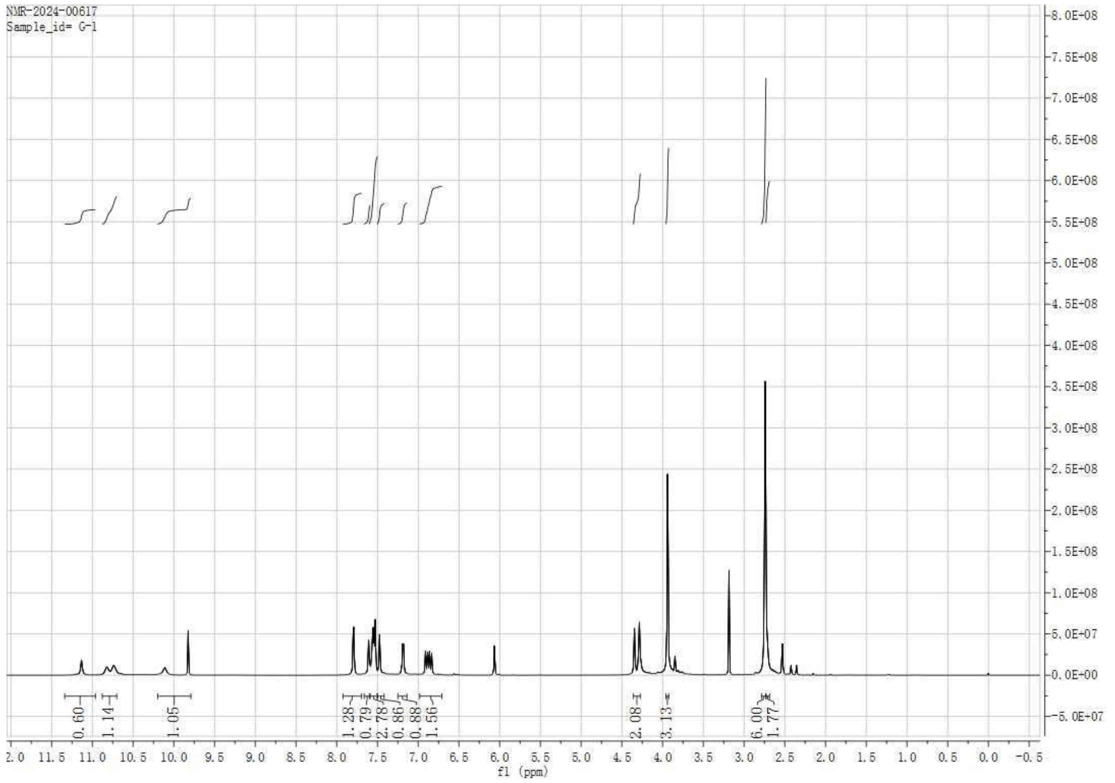

Supplementary Figure S13. ^{1}H-NMR spectrum of **7** in DMSO-d_6.

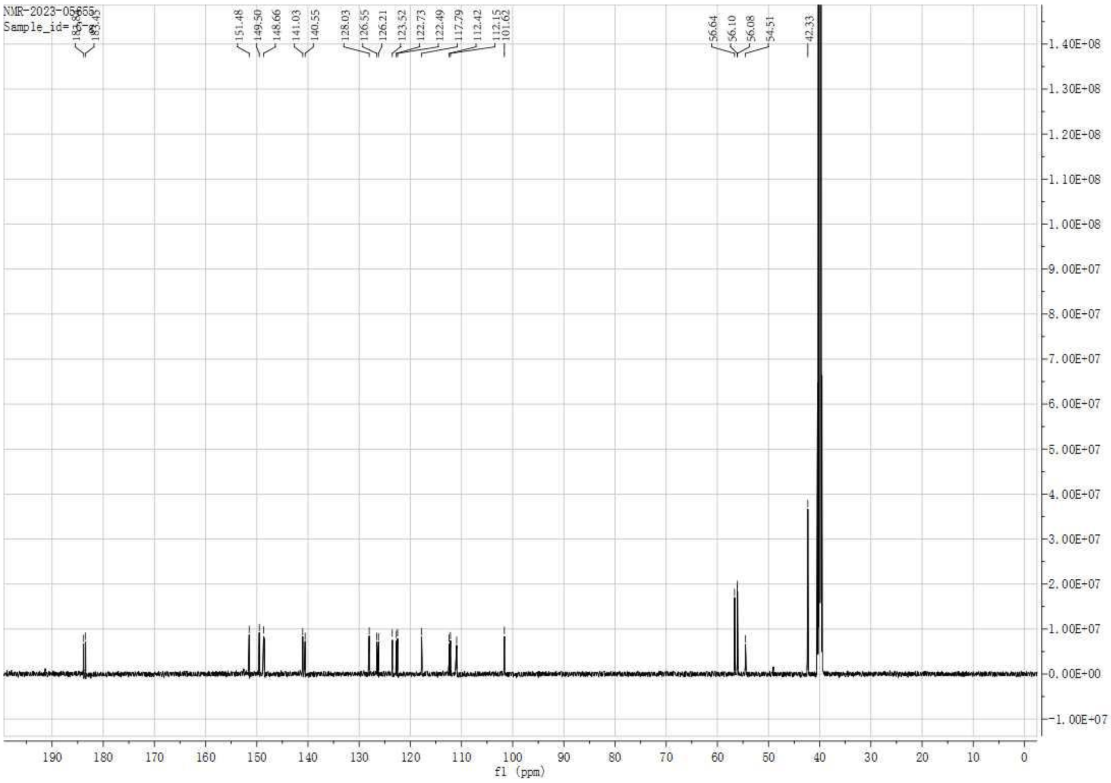

Supplementary Figure S14. ^{13}C-NMR spectrum of **7** in DMSO-d_6.

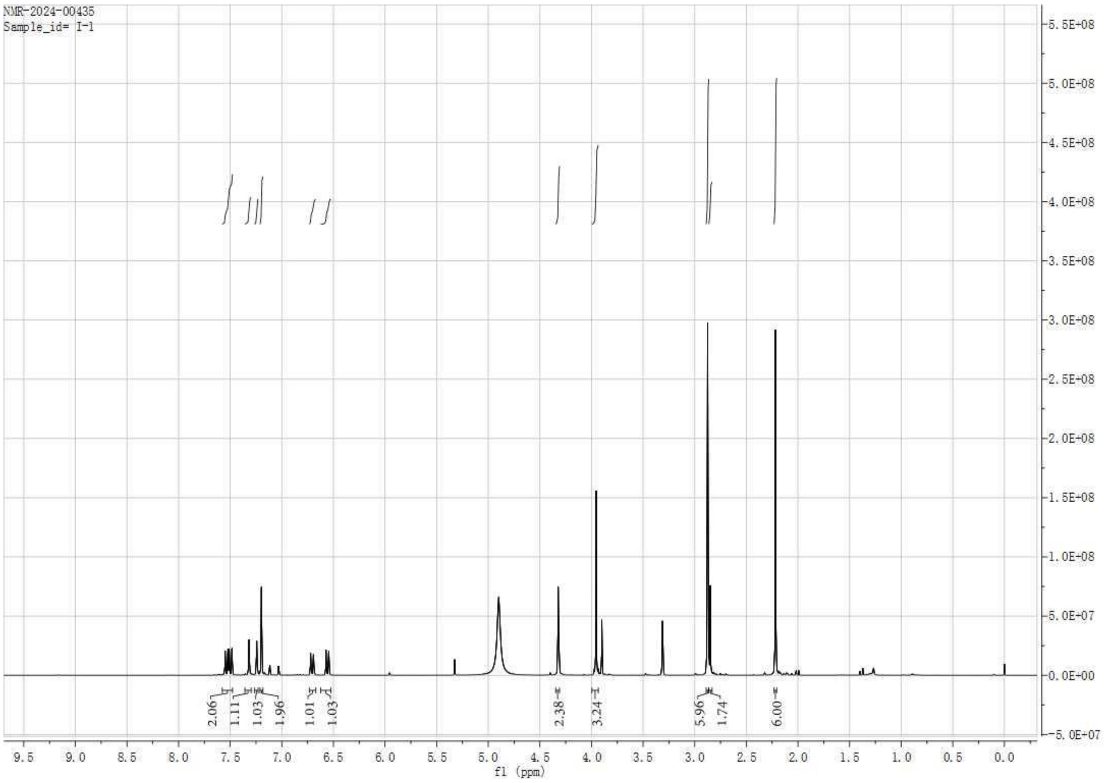

Supplementary Figure S15. ^{1}H-NMR spectrum of **8** in CD_3OD.

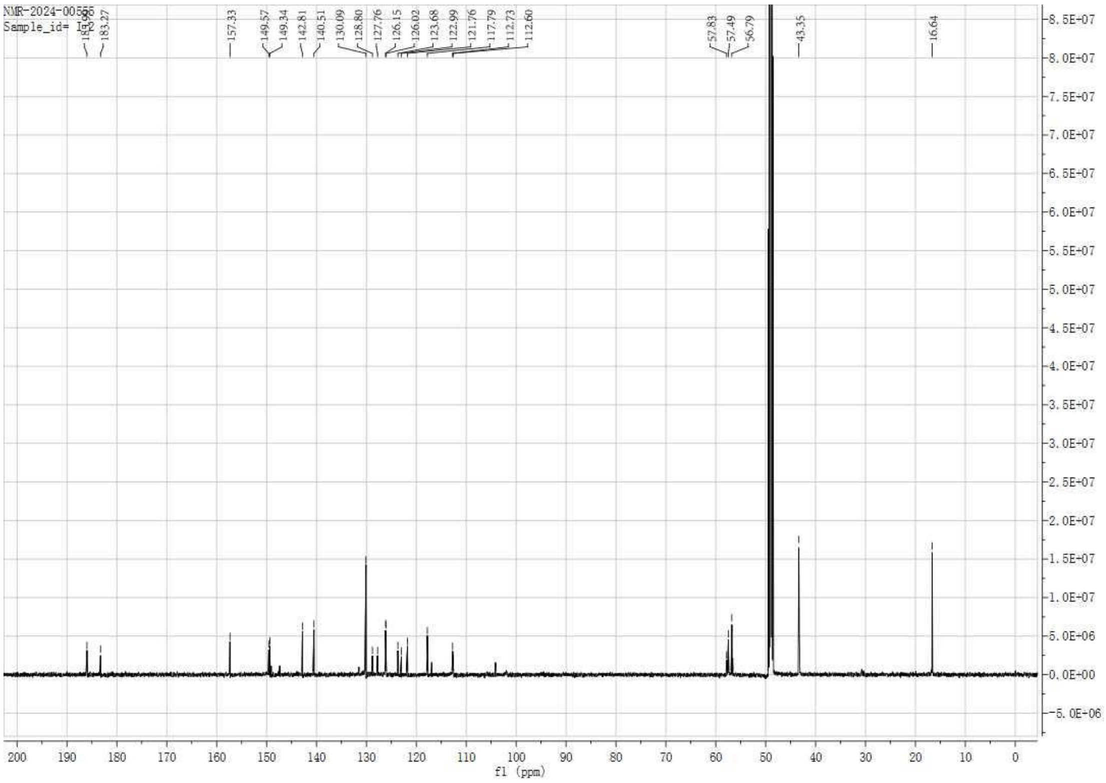

Supplementary Figure S16. ^{13}C-NMR spectrum of **8** in CD$_3$OD.

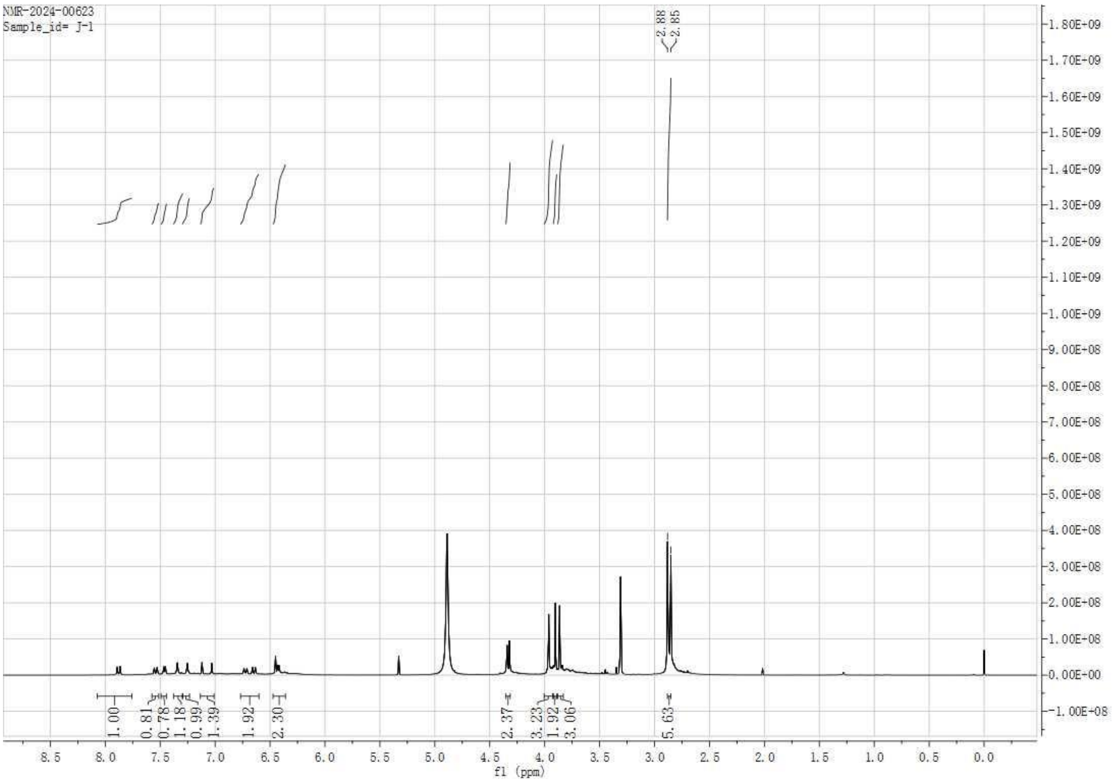

Supplementary Figure S17. ^{1}H-NMR spectrum of **9** in CD$_3$OD.

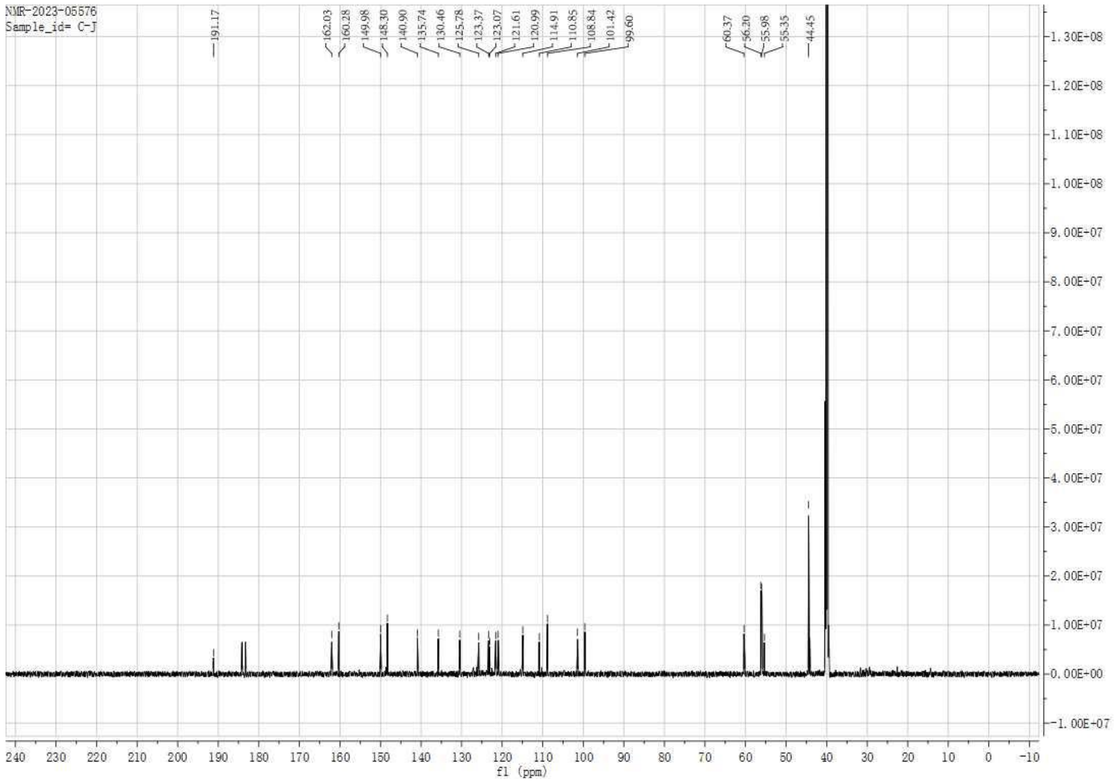

Supplementary Figure S18. ^{13}C-NMR spectrum of **9** in DMSO-d_6.

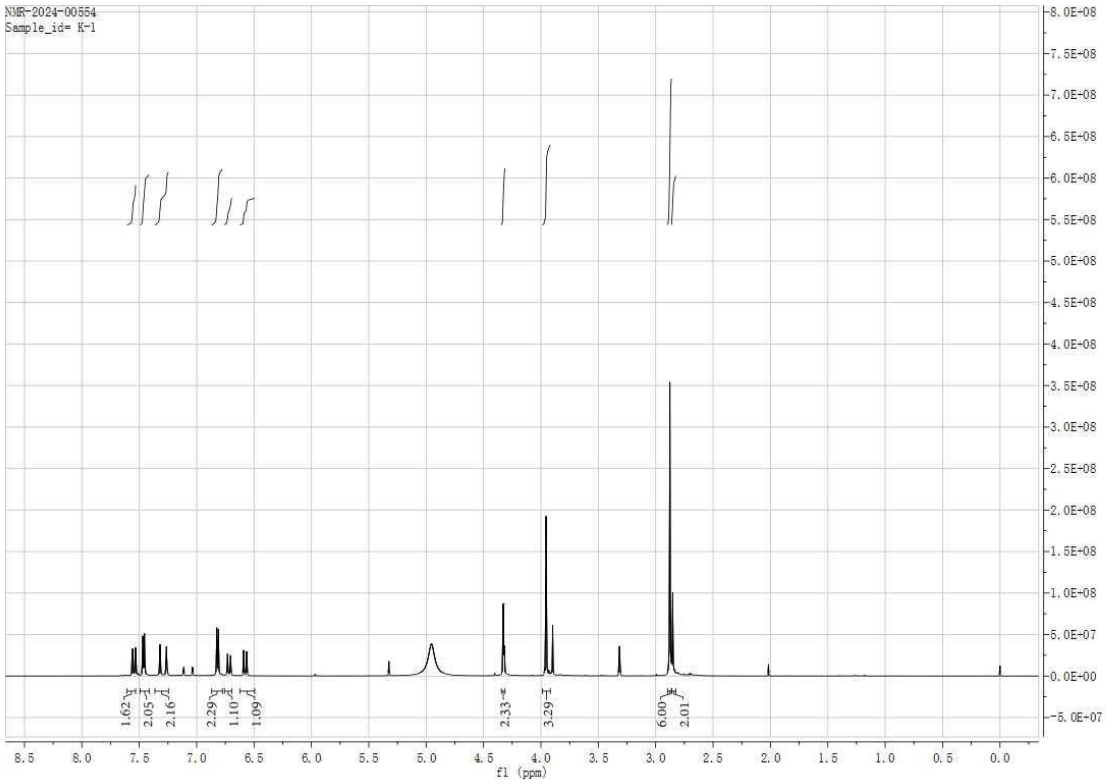

Supplementary Figure S19. ^{1}H-NMR spectrum of **10** in CD_3OD.

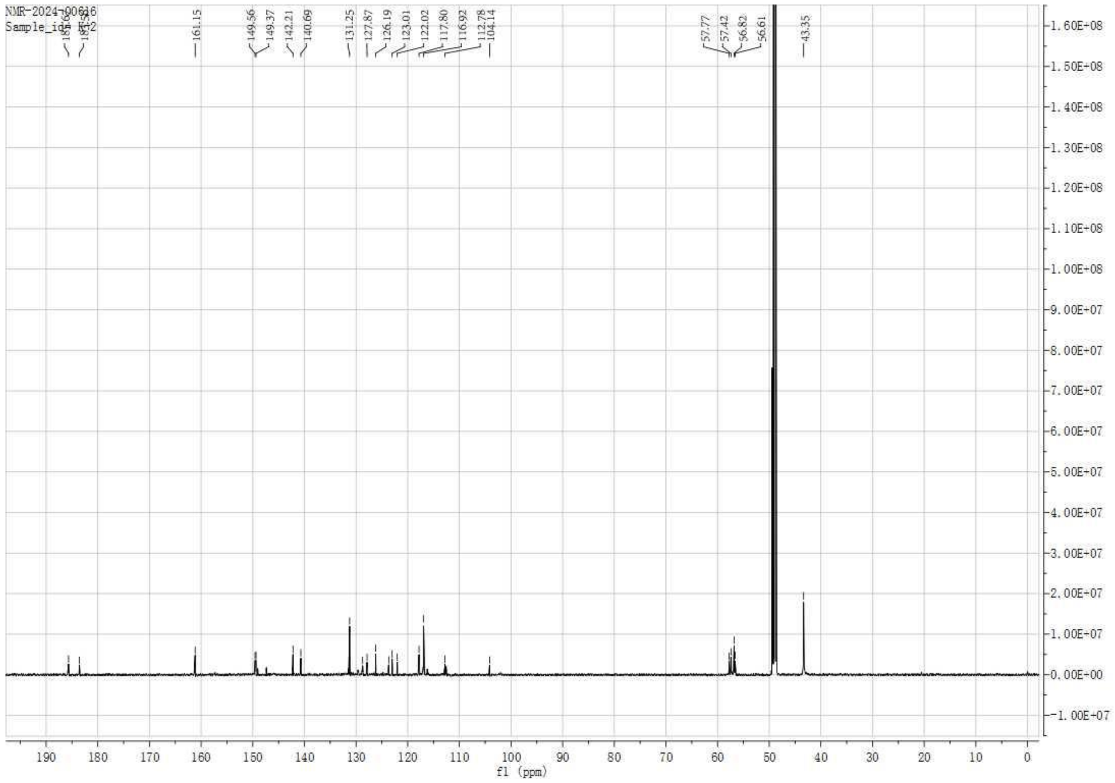

Supplementary Figure S20. ^{13}C-NMR spectrum of **10** in CD_3OD.

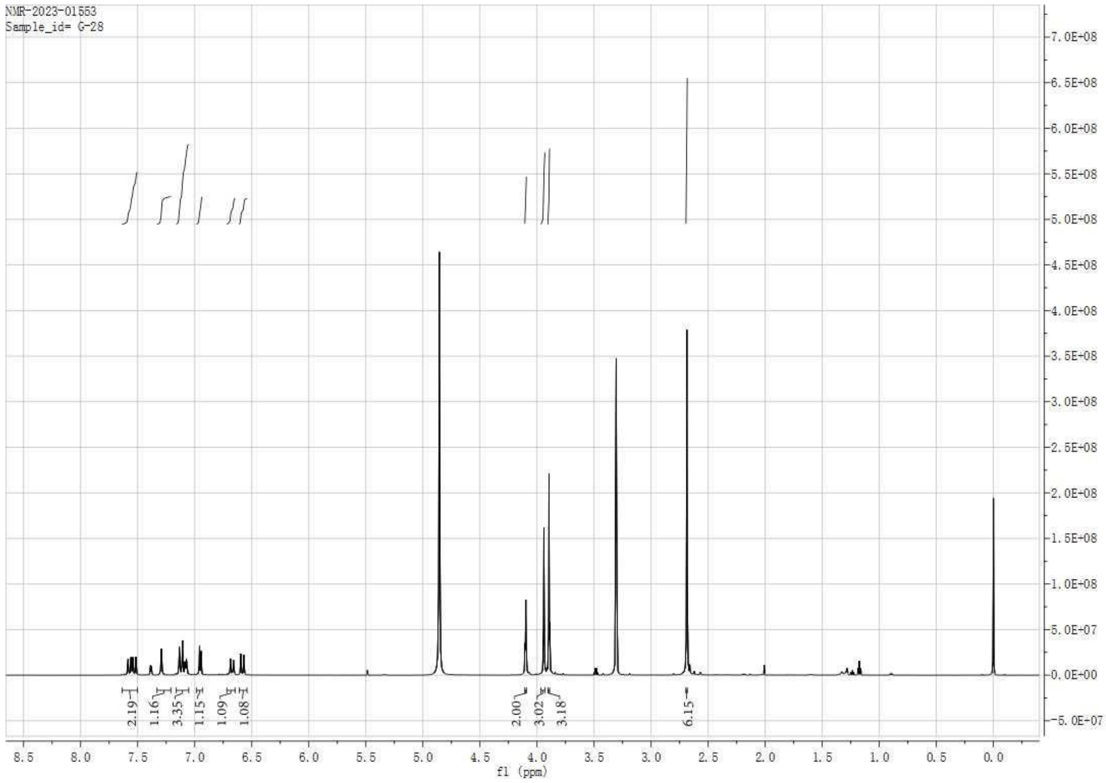

Supplementary Figure S21. ^{1}H-NMR spectrum of **11** in CD_3OD.

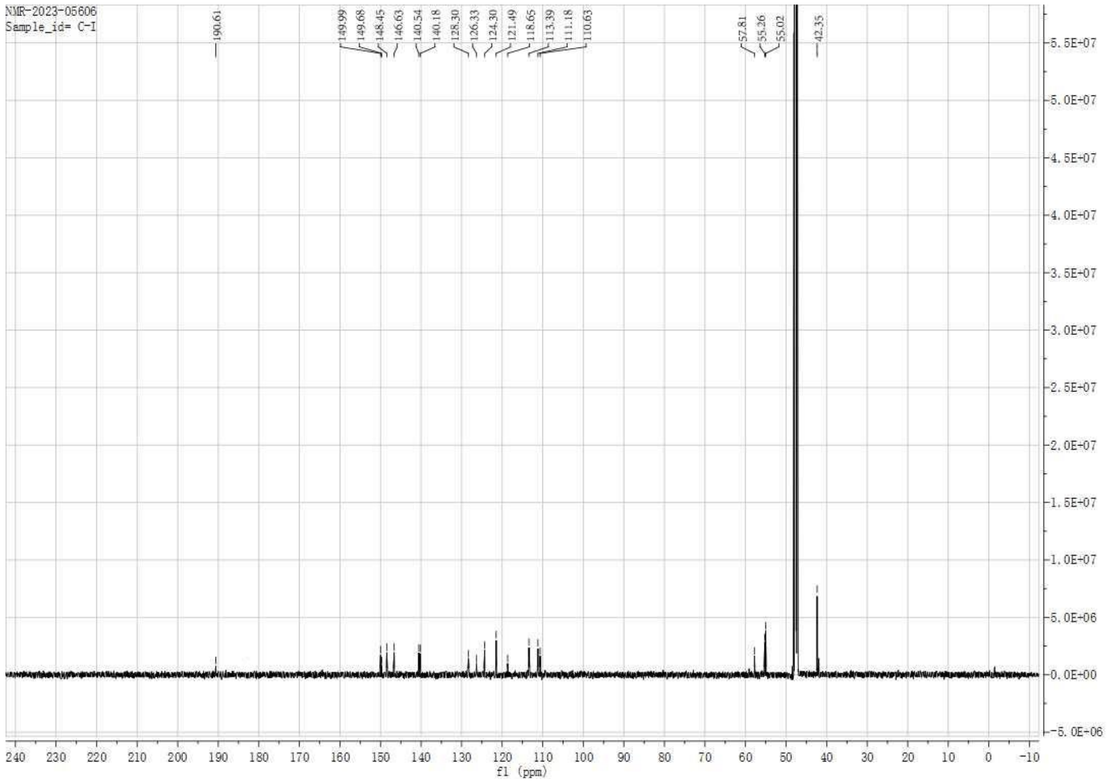

Supplementary Figure S22. ^{13}C-NMR spectrum of **11** in CD_3OD.

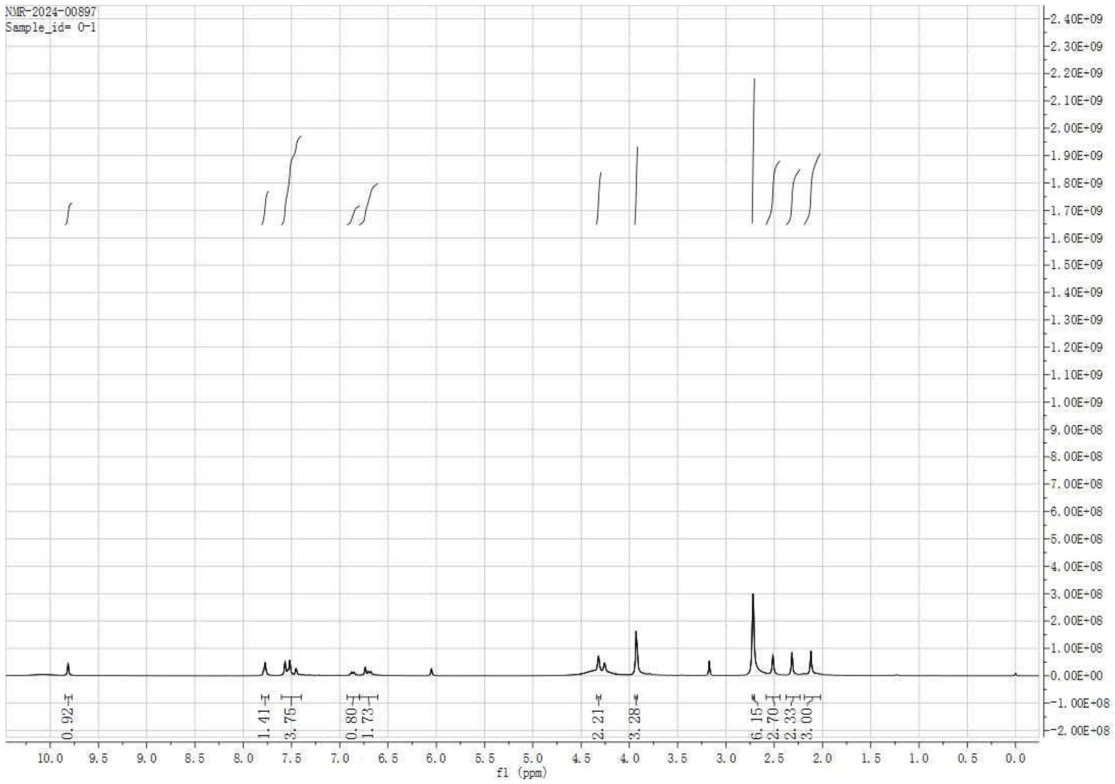

Supplementary Figure S23. ^{1}H-NMR spectrum of **12** in DMSO-d_6.

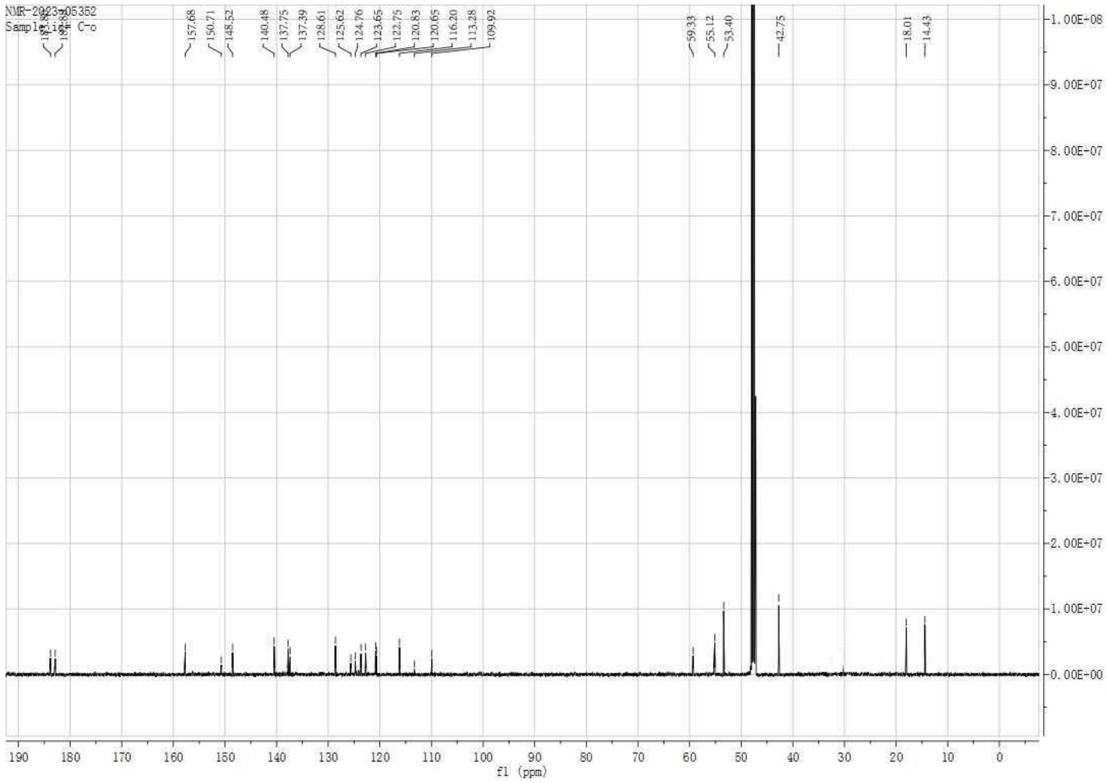

Supplementary Figure S24. ^{13}C-NMR spectrum of **12** in CD$_3$OD.

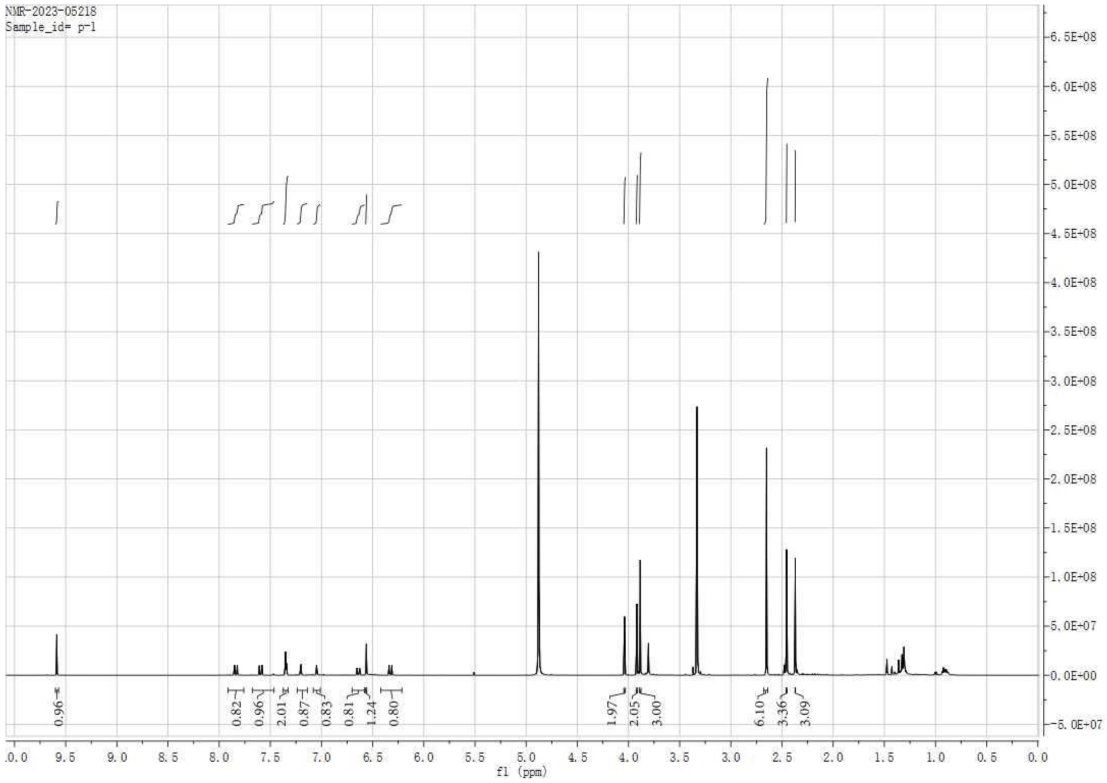

Supplementary Figure S25. ^{1}H-NMR spectrum of **13** in CD$_3$OD.

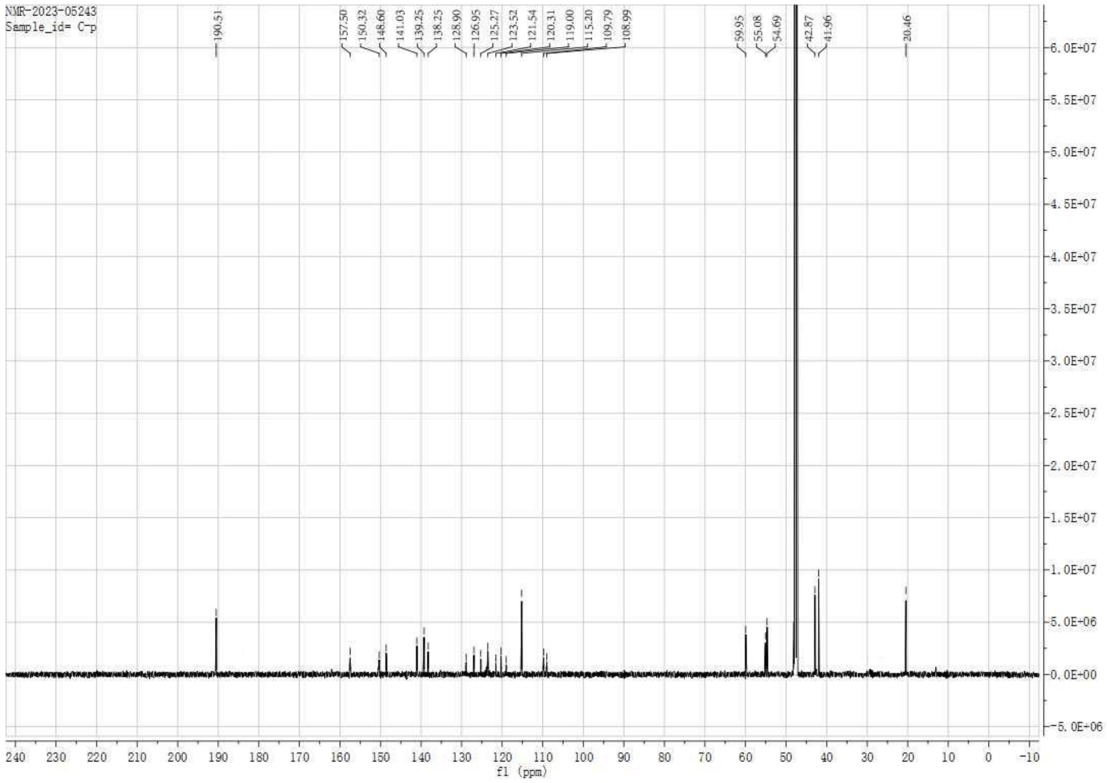

Supplementary Figure S26. ^{13}C-NMR spectrum of **13** in CD_3OD.

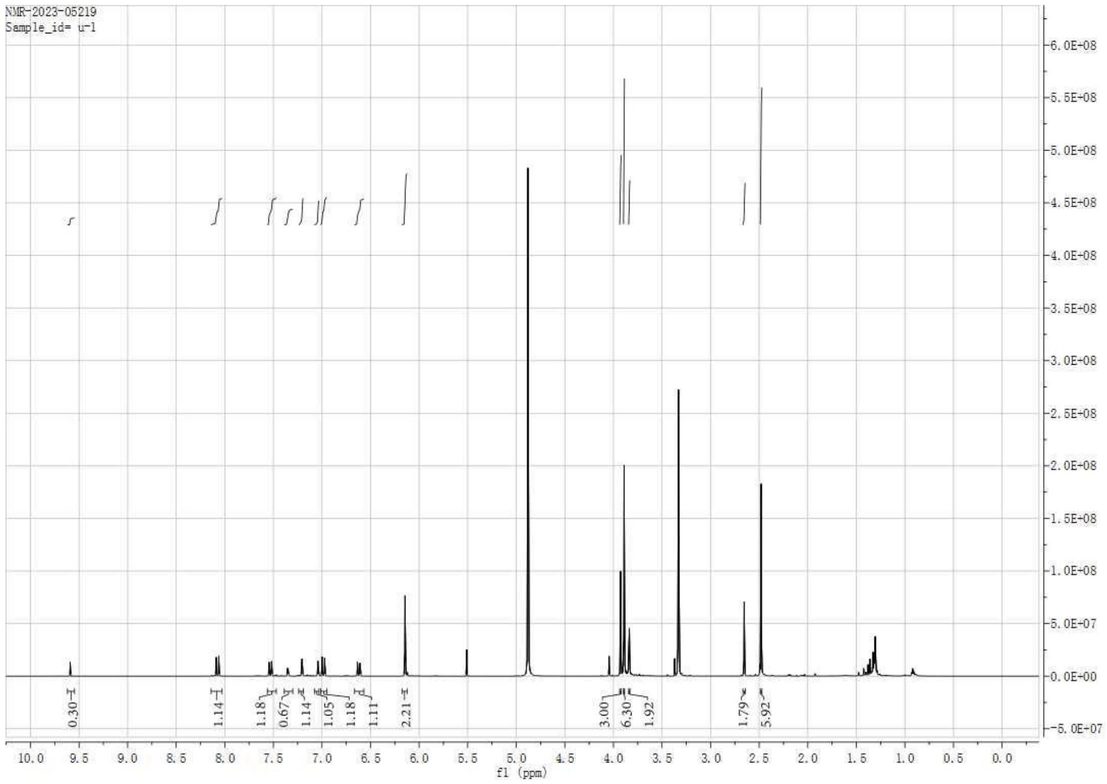

Supplementary Figure S27. ^{1}H-NMR spectrum of **14** in CD_3OD.

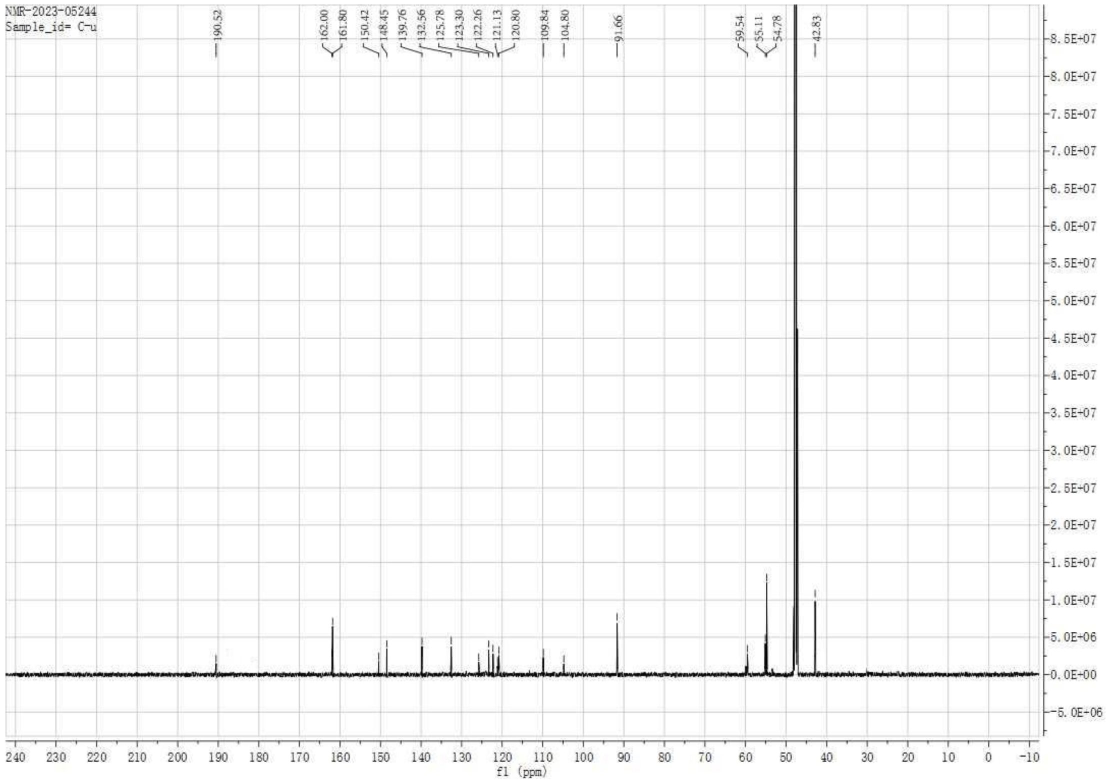

Supplementary Figure S28. ^{13}C-NMR spectrum of **14** in CD_3OD.

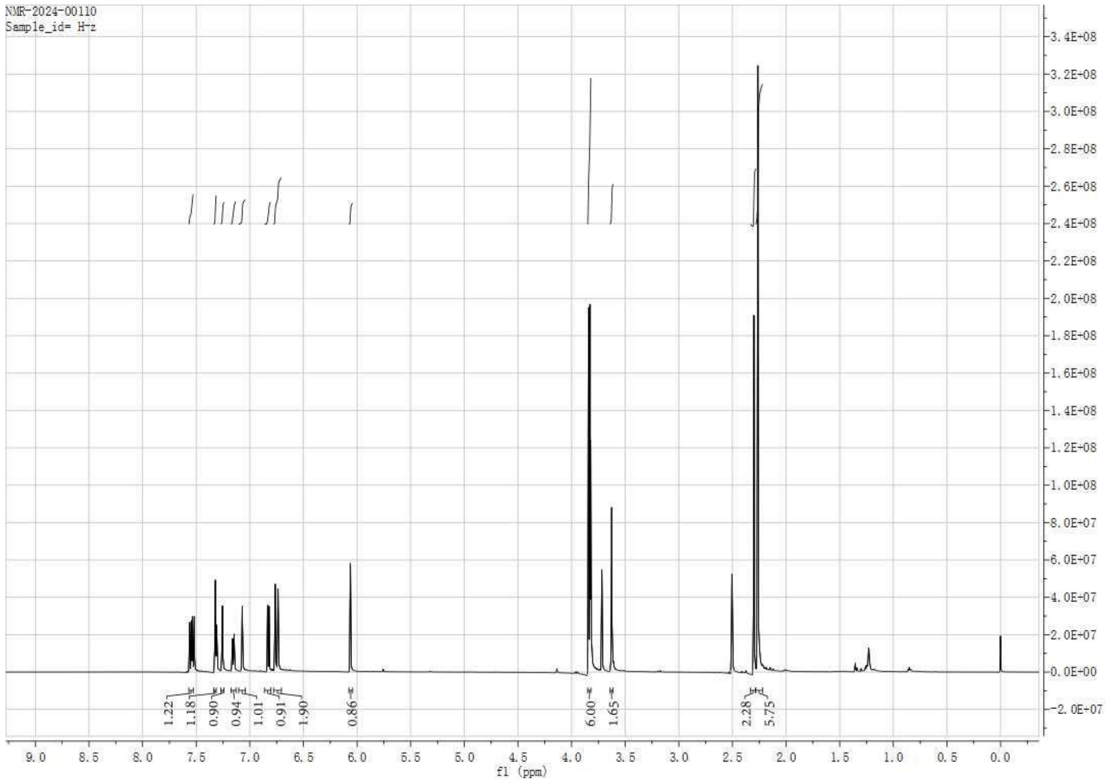

Supplementary Figure S29. ^{1}H-NMR spectrum of **15** in DMSO-d_6.

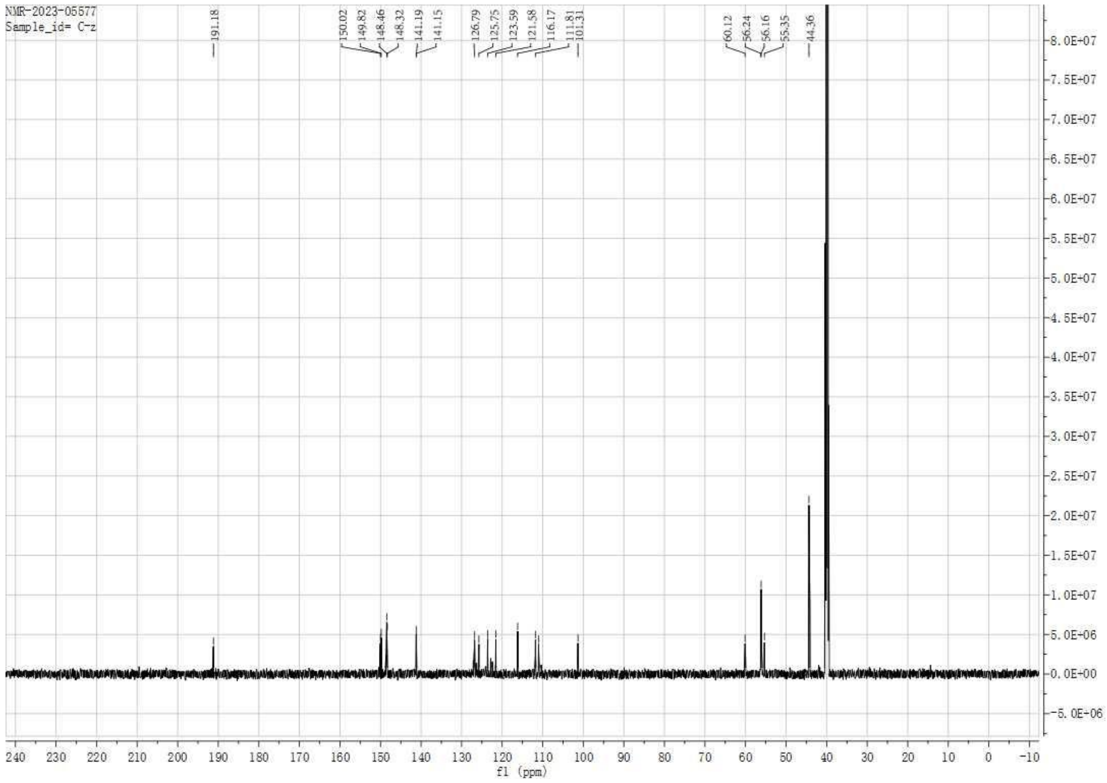

Supplementary Figure S30. ^{13}C-NMR spectrum of **15** in DMSO-d_6.

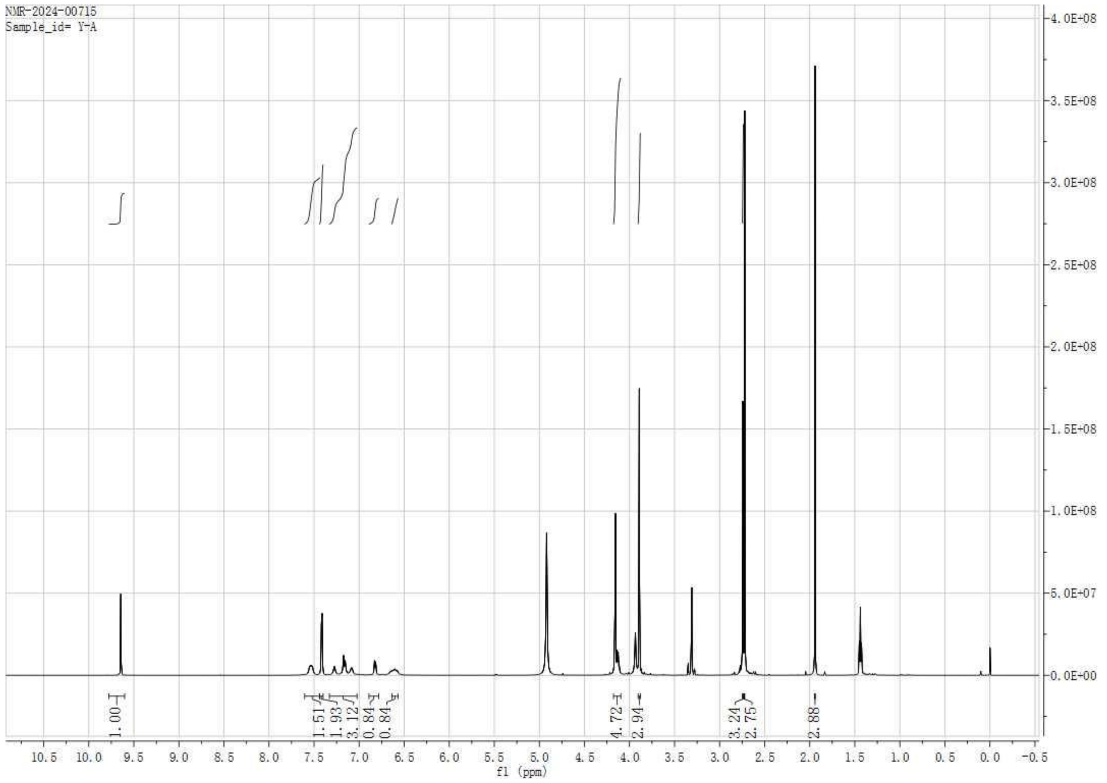

Supplementary Figure S31. ^{1}H-NMR spectrum of **1a** in CD_3OD.

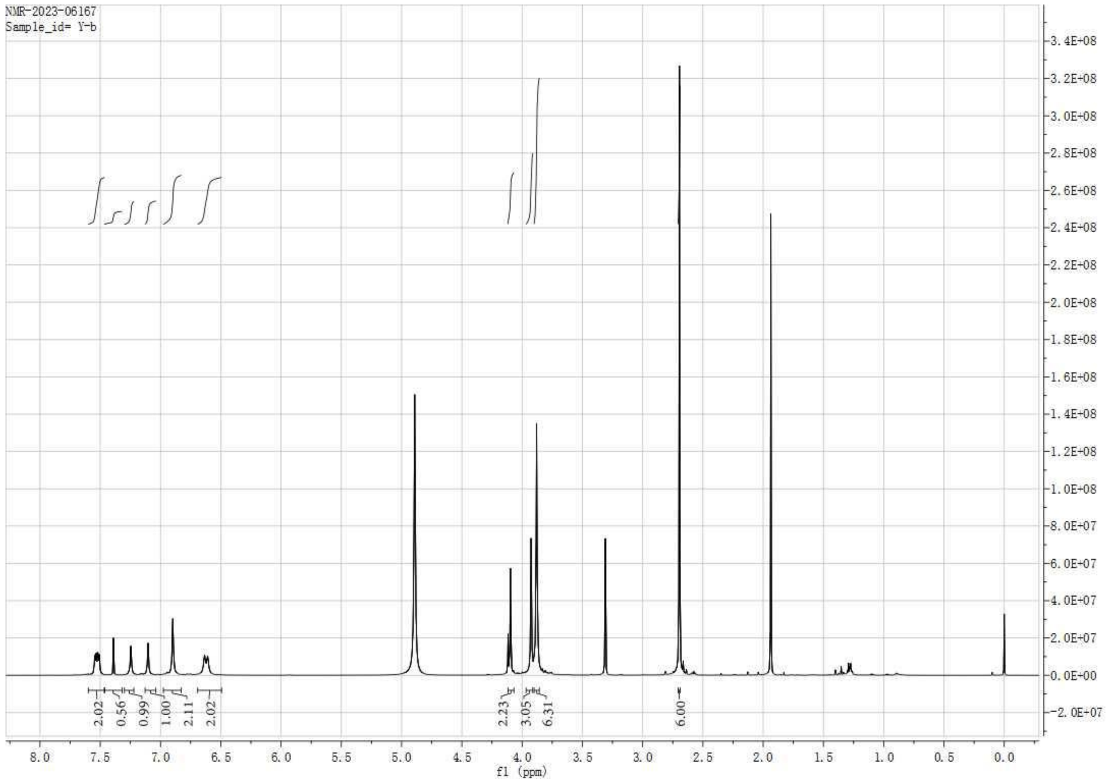

Supplementary Figure S32. ^{1}H-NMR spectrum of **2a** in CD_3OD.

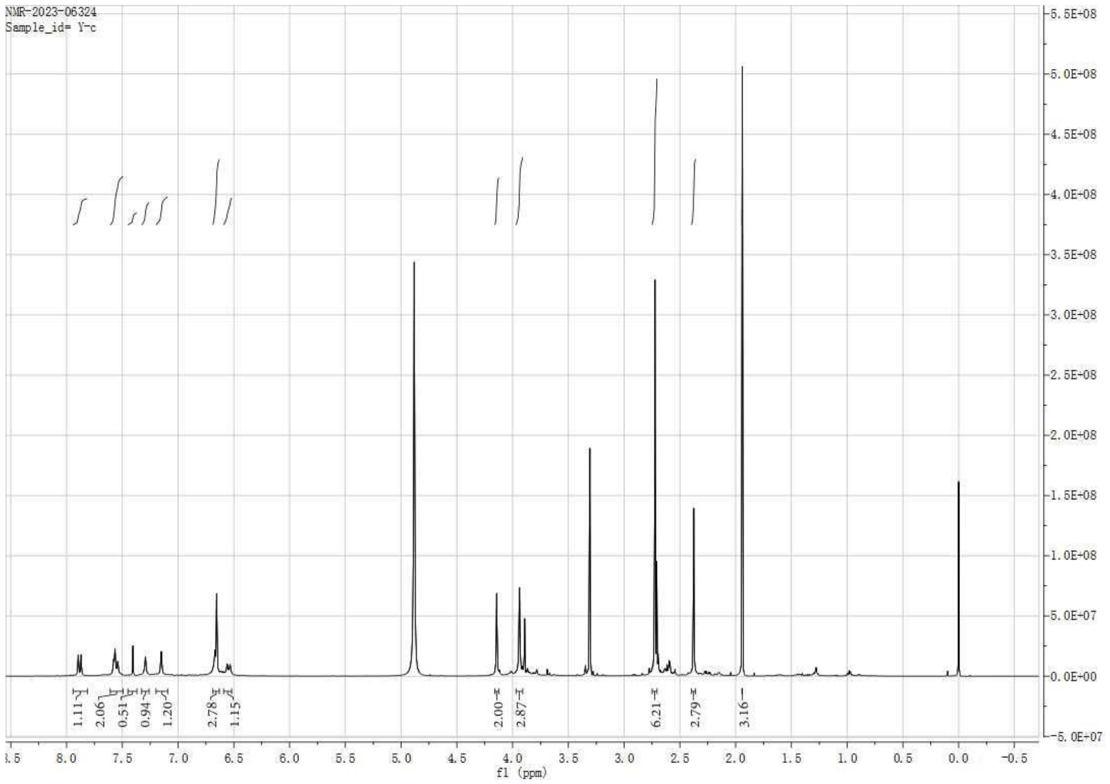

Supplementary Figure S33. ^{1}H-NMR spectrum of **3a** in CD_3OD.

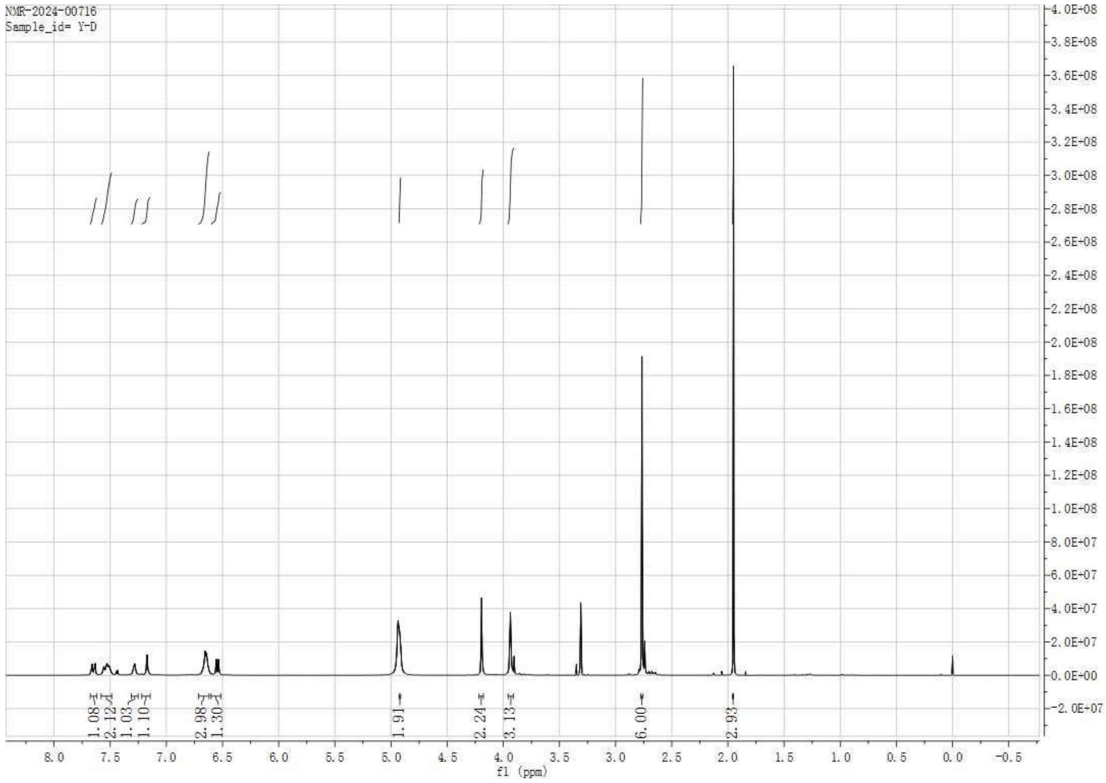

Supplementary Figure S34. ^{1}H-NMR spectrum of **4a** in CD_3OD.

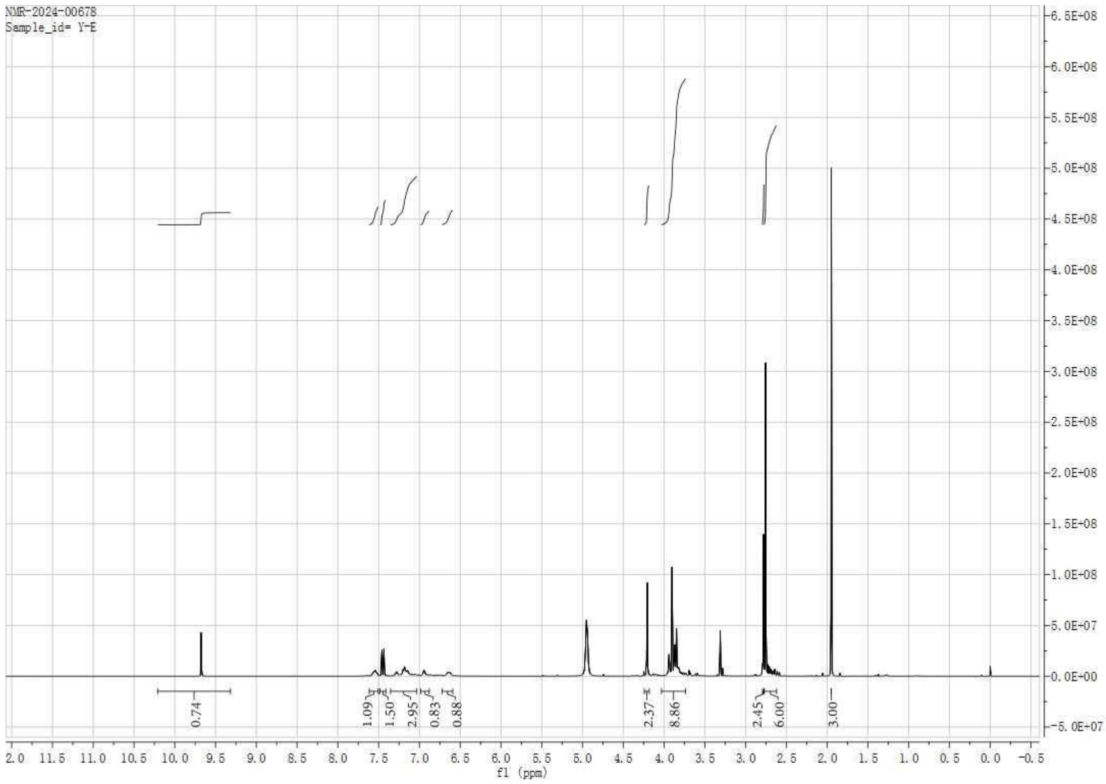

Supplementary Figure S35. ^{1}H-NMR spectrum of **5a** in CD_3OD.

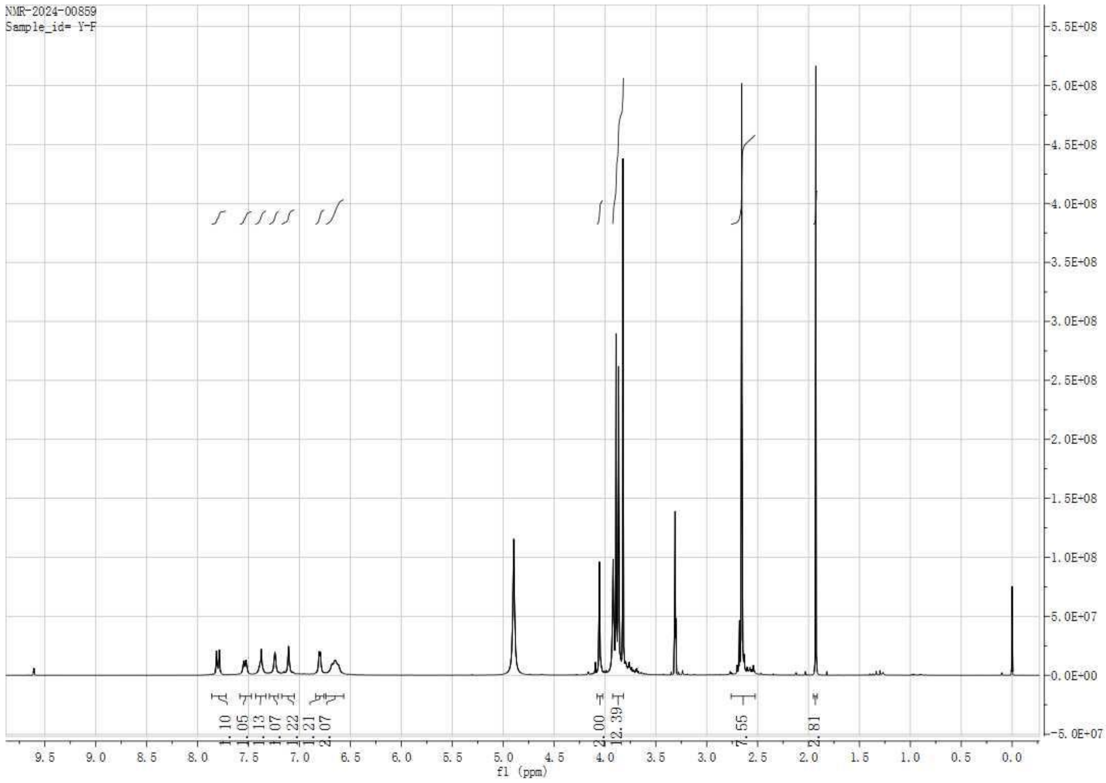

Supplementary Figure S36. ^{1}H-NMR spectrum of **6a** in CD_3OD.

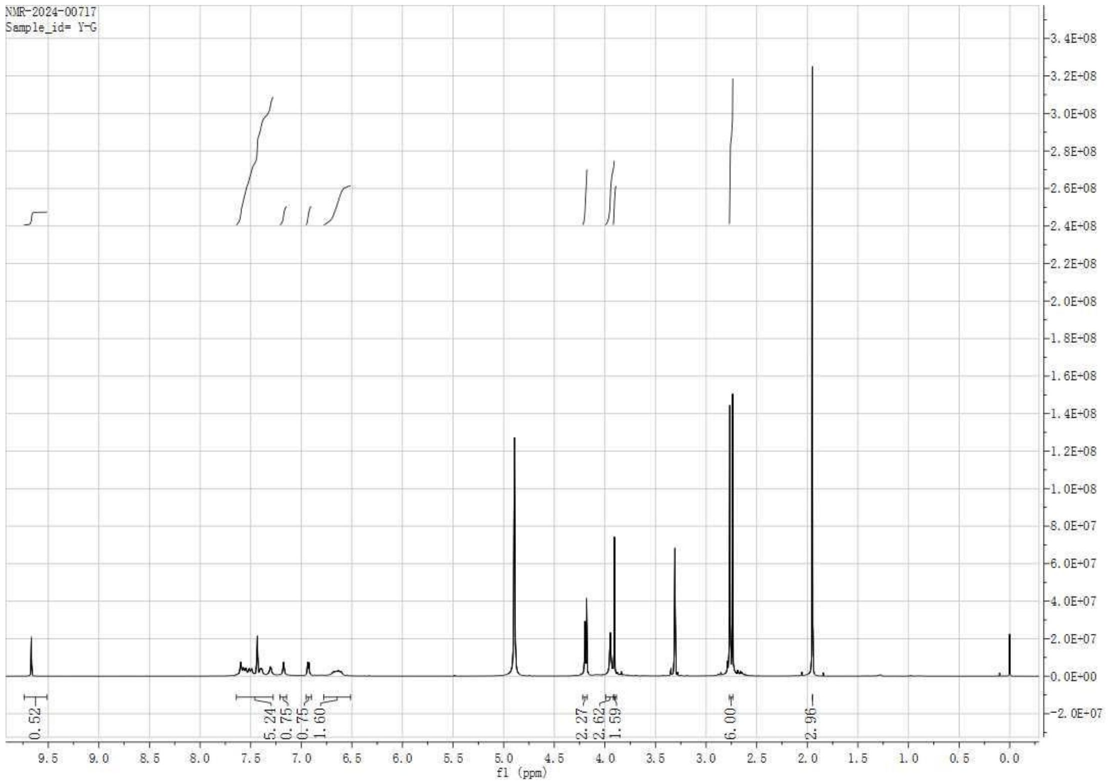

Supplementary Figure S37. ^{1}H-NMR spectrum of **7a** in CD_3OD.

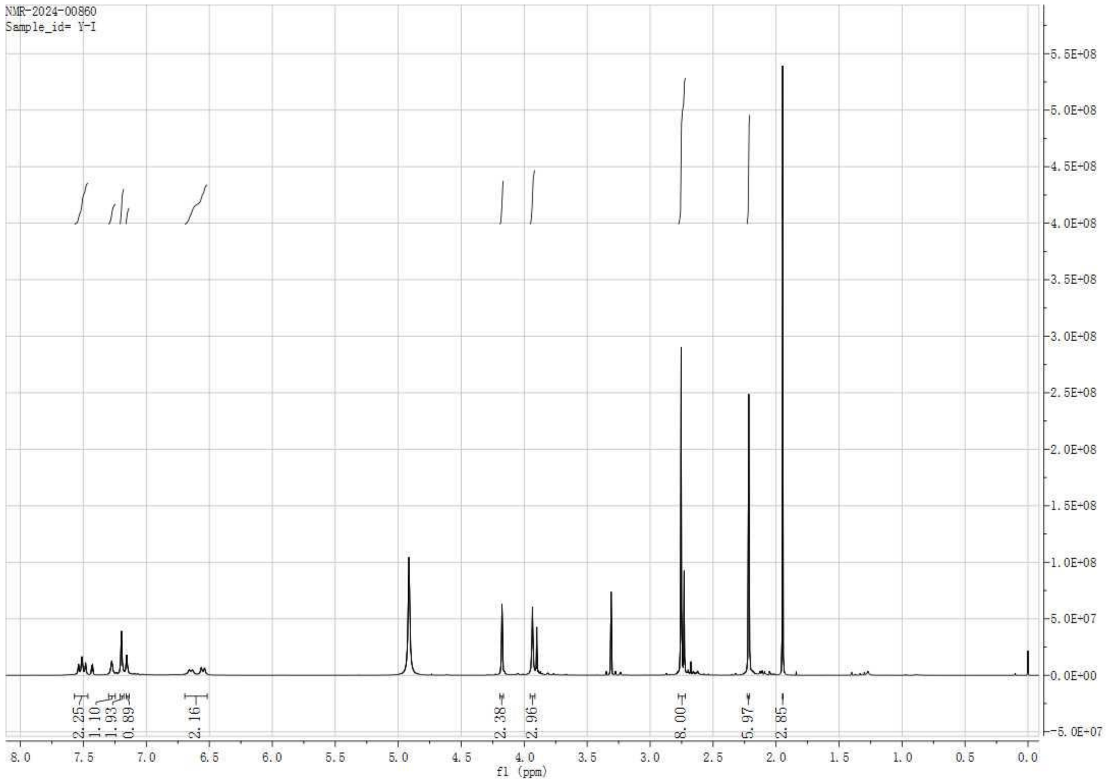

Supplementary Figure S38. ^{1}H-NMR spectrum of **8a** in CD_3OD.

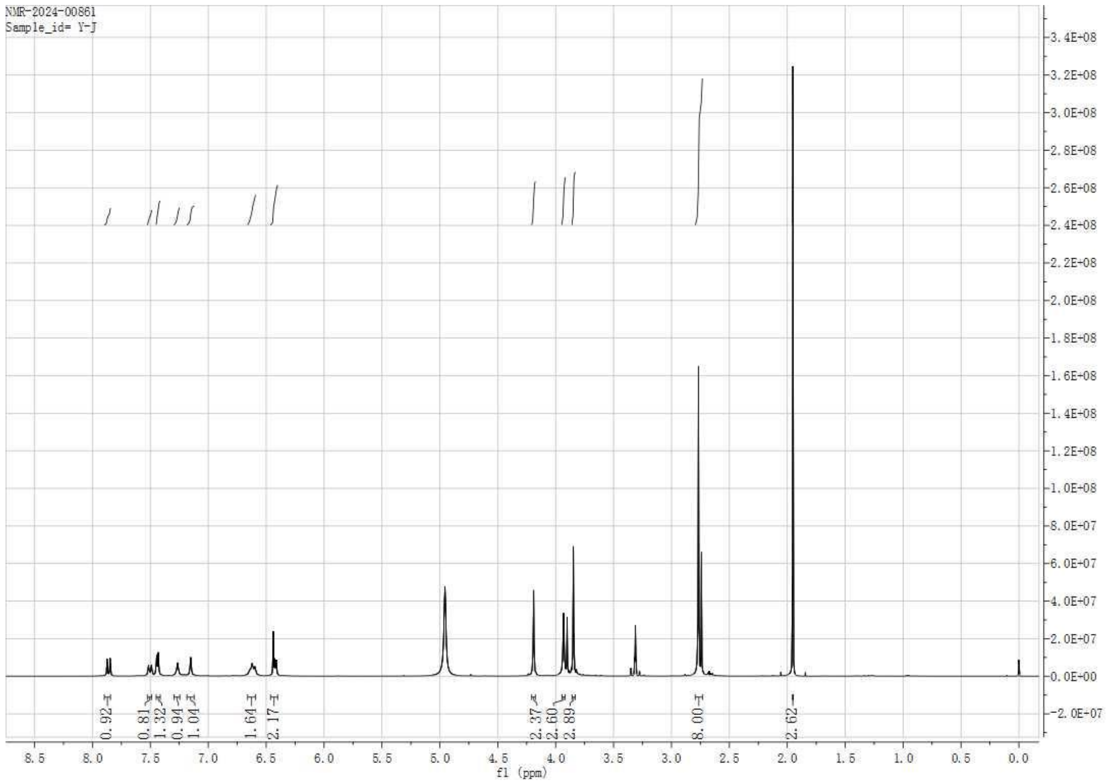

Supplementary Figure S39. ^{1}H-NMR spectrum of **9a** in CD_3OD.

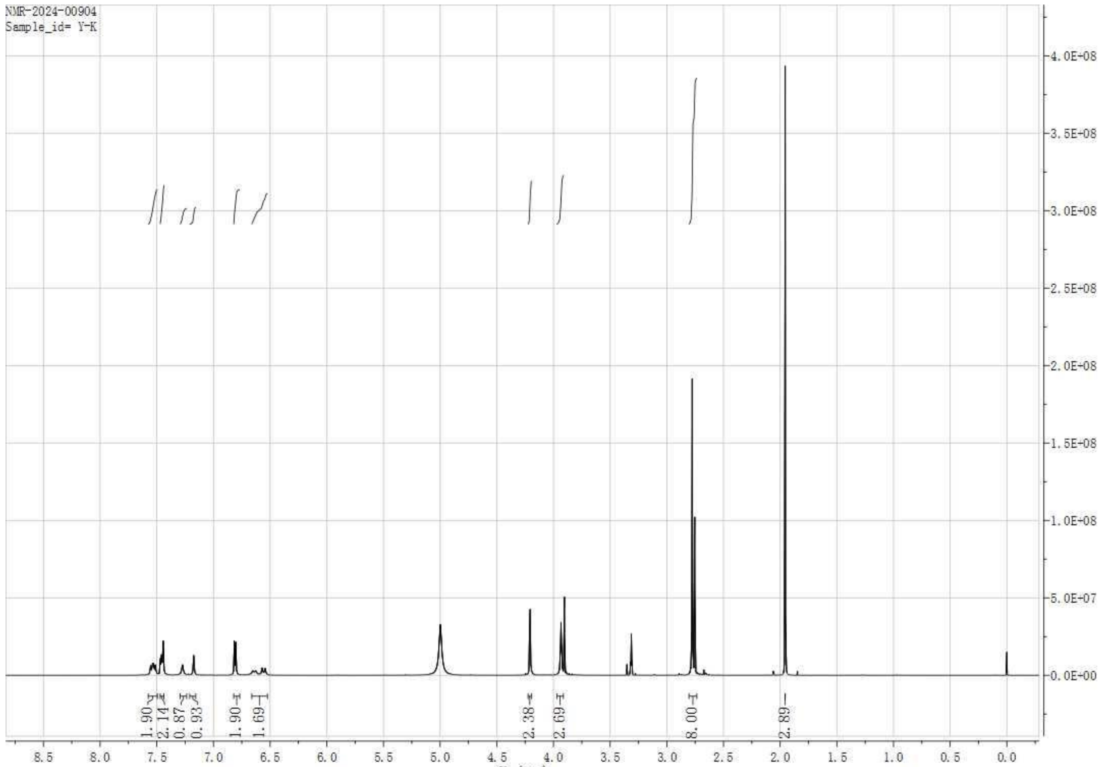

Supplementary Figure S40. ¹H-NMR spectrum of **10a** in CD$_3$OD.

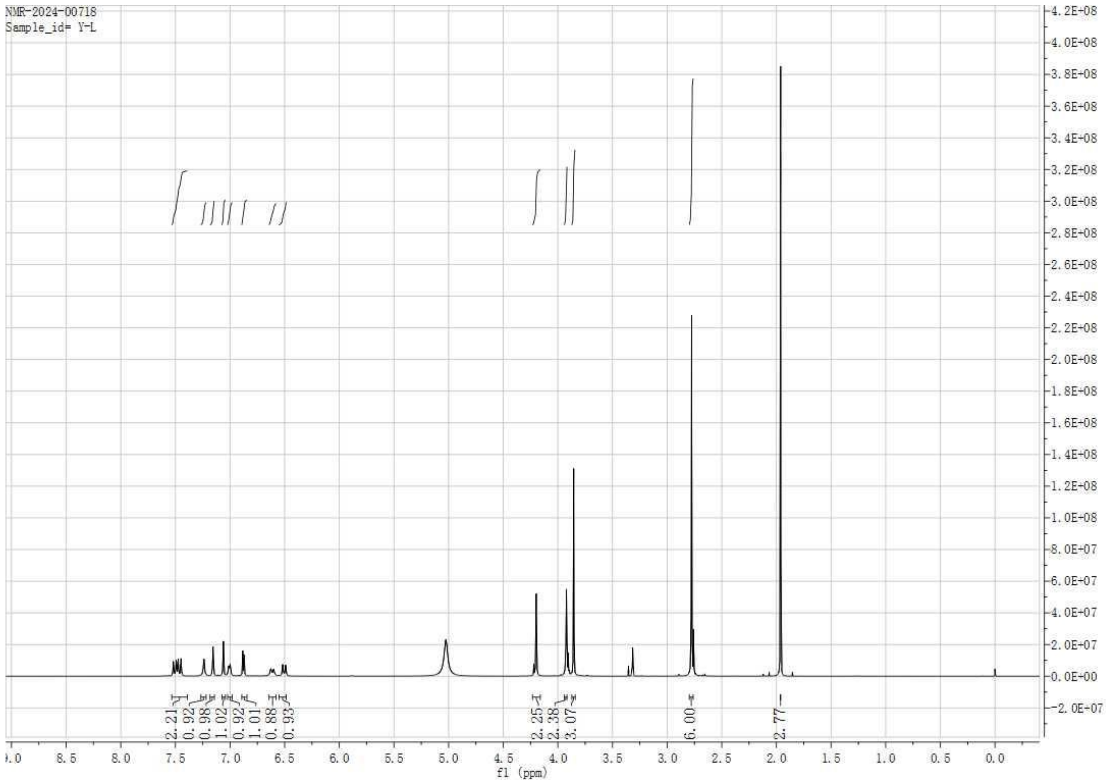

Supplementary Figure S41. ¹H-NMR spectrum of **11a** in CD$_3$OD.

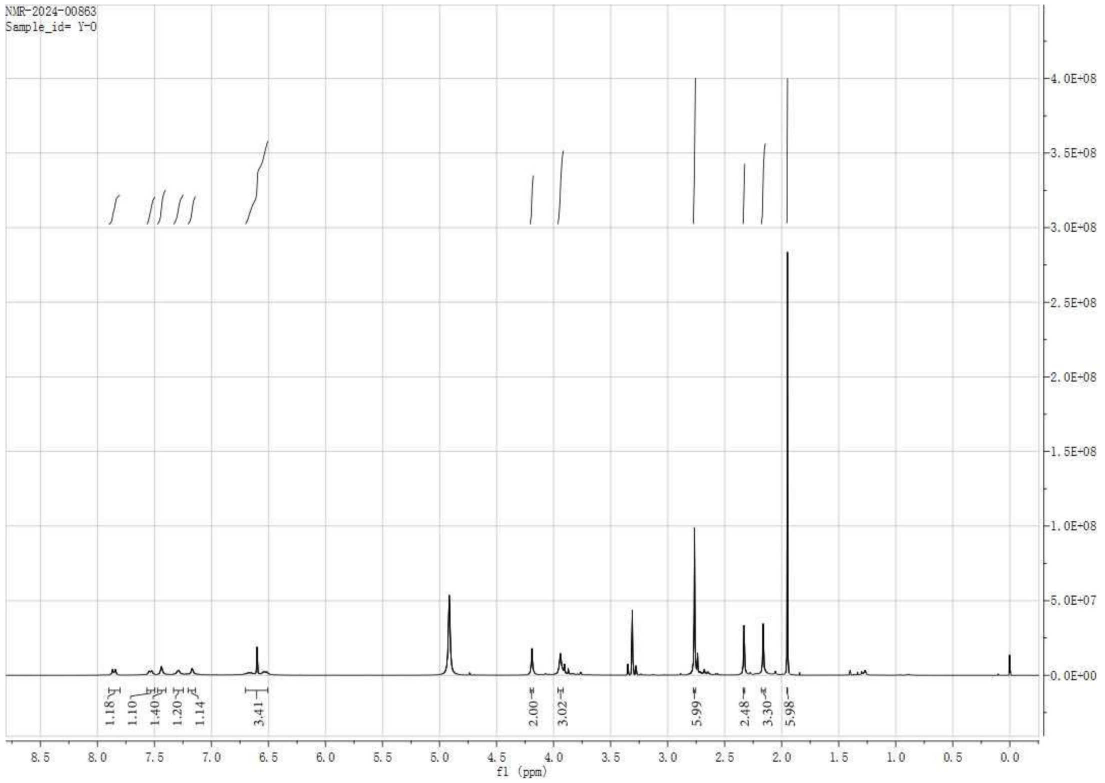

Supplementary Figure S42. ^{1}H-NMR spectrum of **12a** in CD_3OD.

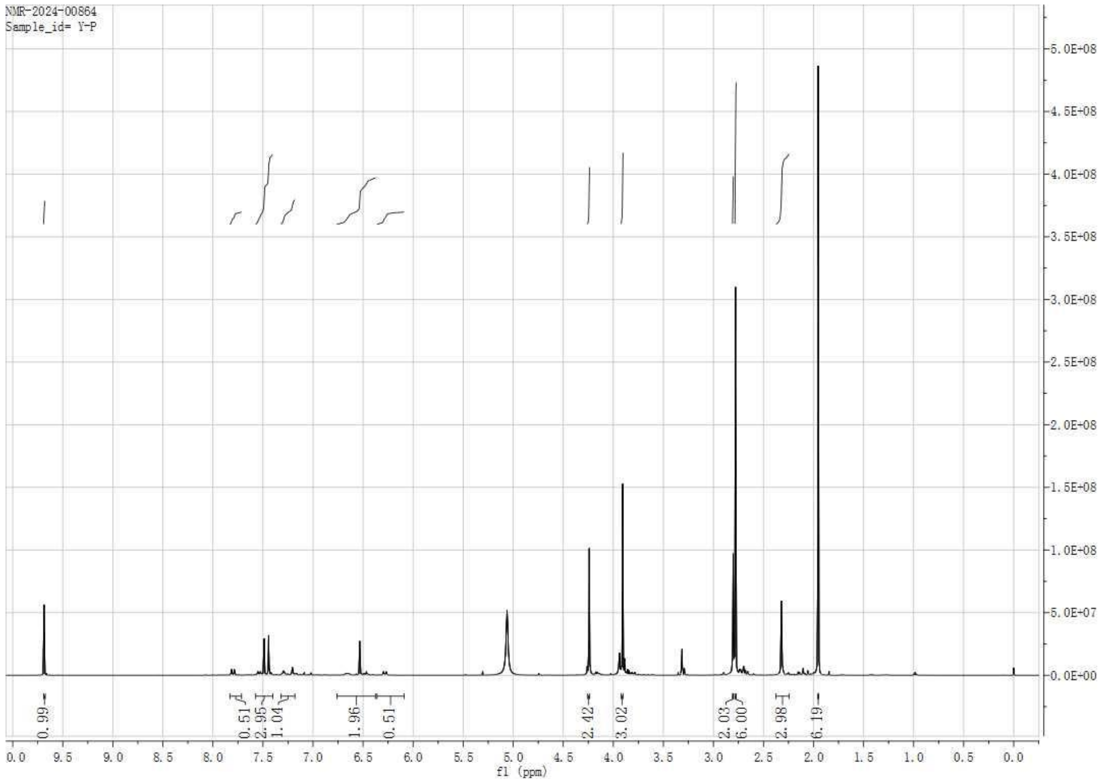

Supplementary Figure S43. ^{1}H-NMR spectrum of **13a** in CD_3OD.

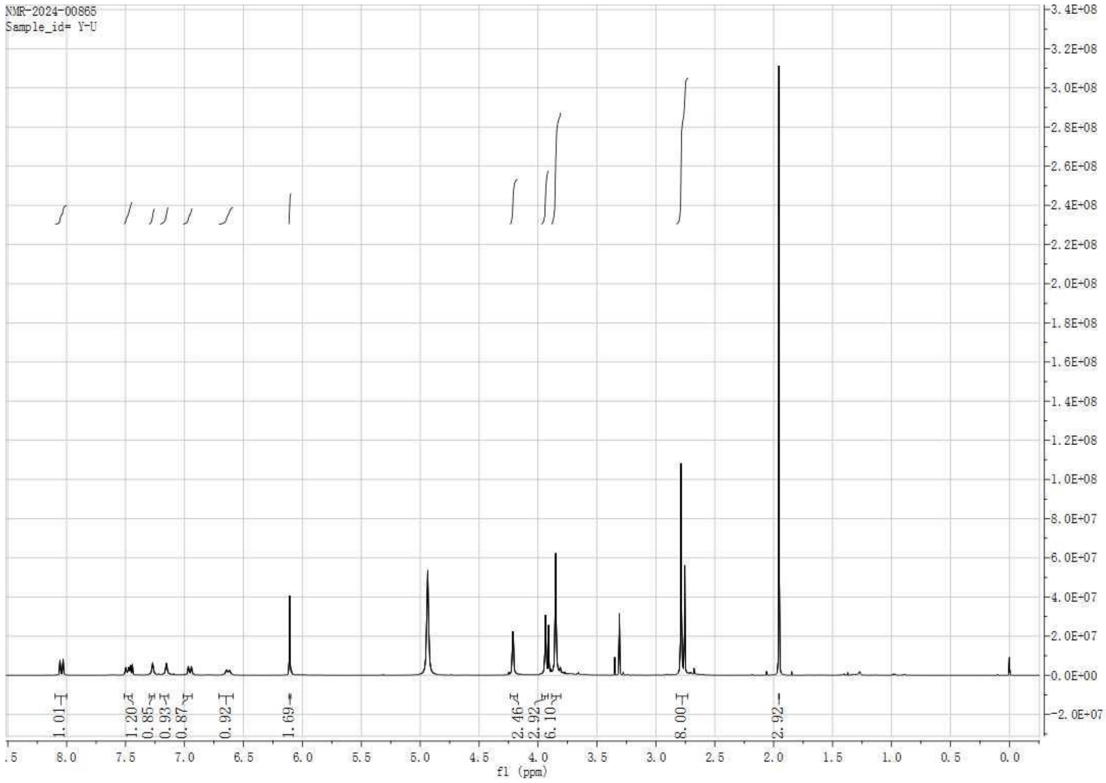

Supplementary Figure S44. ^{1}H-NMR spectrum of **14a** in CD_3OD.

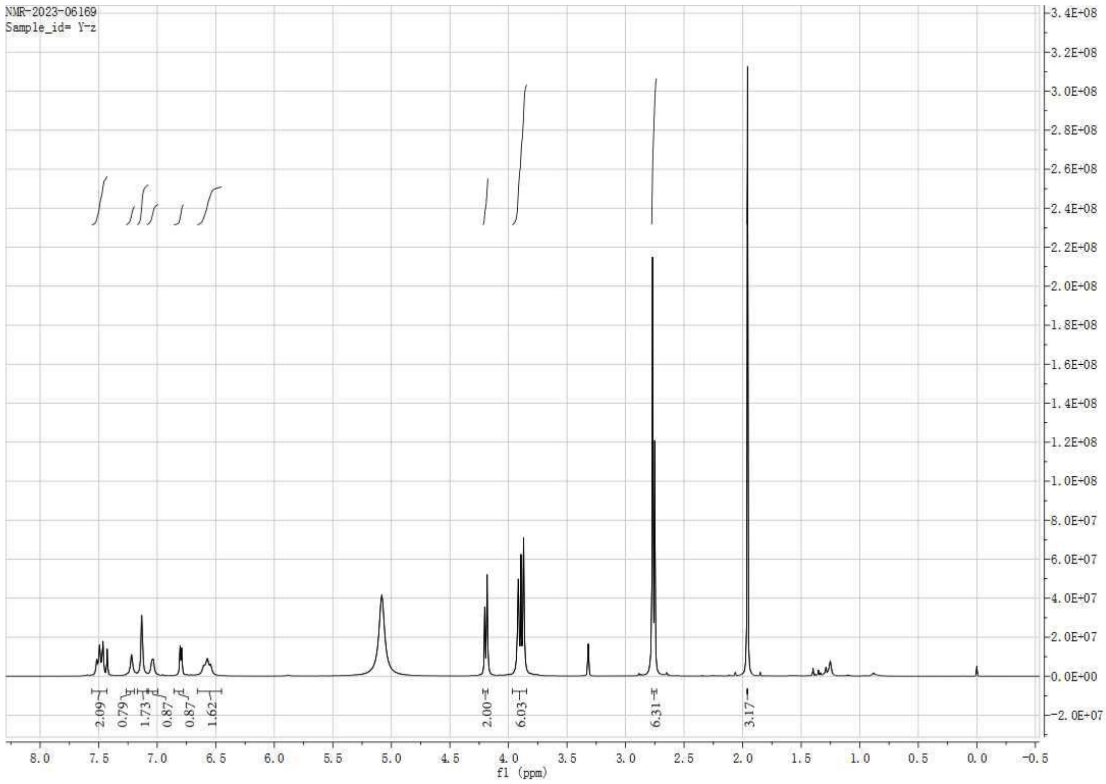

Supplementary Figure S45. ^{1}H-NMR spectrum of **15a** in CD_3OD.

Qualitative Analysis Report

**Data Filename**	2456.d	**Sample Name**	L 2
**Instrument Name**	TOF G6230A	**Acquired Time**	2023-04-23
**Acq Method**	YCL.M	**Acquired SW**	6200 series TOF/6500 series
**IRM Calibration Status**	**Success**		
**User Chormatograms**			

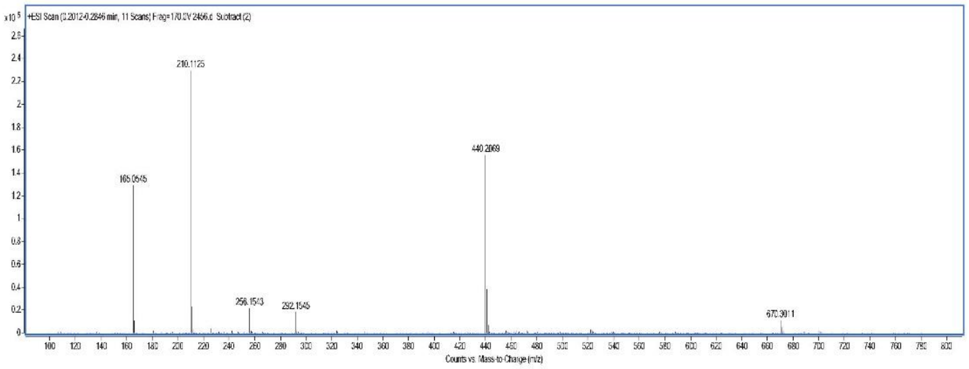

Supplementary Figure S46. HR-ESI-MS spectrum of **1**.

Qualitative Analysis Report

**Data Filename**	2457.d	**Sample Name**	L 4
**Instrument Name**	TOF G6230A	**Acquired Time**	2023-04-23
**Acq Method**	YCL.M	**Acquired SW**	6200 series TOF/6500 series
**IRM Calibration Status**	**Success**		
**User Chormatograms**			

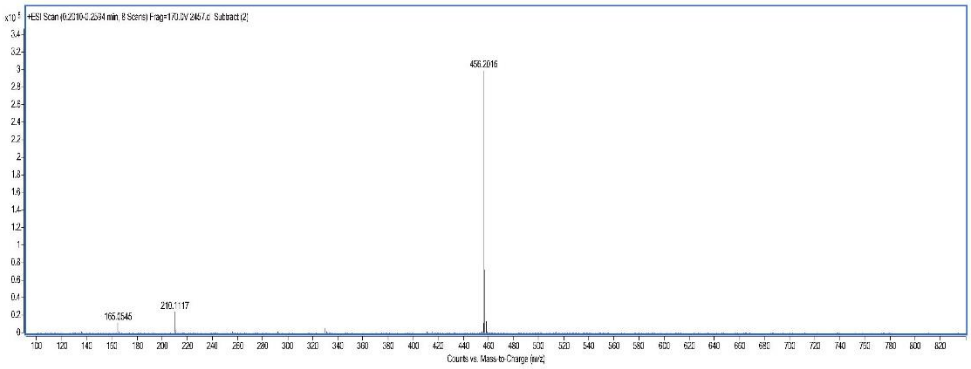

Supplementary Figure S47. HR-ESI-MS spectrum of **2**.

Qualitative Analysis Report

**Data Filename**	2171.d	**Sample Name**	L-6
**Instrument Name**	TOF G6230A	**Acquired Time**	2023-04-13
**Acq Method**	YCL.M	**Acquired SW**	6200 series TOF/6500 series
**IRM Calibration Status**	**Success**		
**User Chormatograms**			

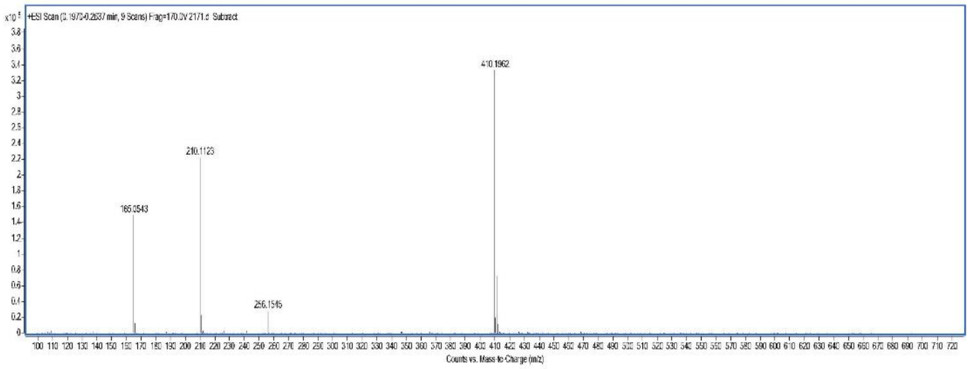

Supplementary Figure S48. HR-ESI-MS spectrum of **3**.

Qualitative Analysis Report

**Data Filename**	2172.d	**Sample Name**	L-8
**Instrument Name**	TOF G6230A	**Acquired Time**	2023-04-13
**Acq Method**	YCL.M	**Acquired SW**	6200 series TOF/6500 series
**IRM Calibration Status**	**Success**		
**User Chormatograms**			

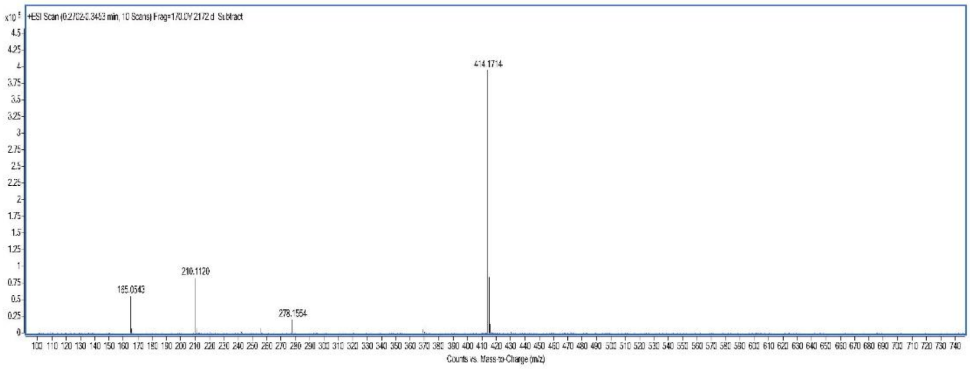

Supplementary Figure S49. HR-ESI-MS spectrum of **4**.

Qualitative Analysis Report

**Data Filename**	2176.d	**Sample Name**	L 10
**Instrument Name**	TOF G6230A	**Acquired Time**	2023-04-13
**Acq Method**	YCL.M	**Acquired SW**	6200 series TOF/6500 series
**IRM Calibration Status**	**Success**		
**User Chormatograms**			

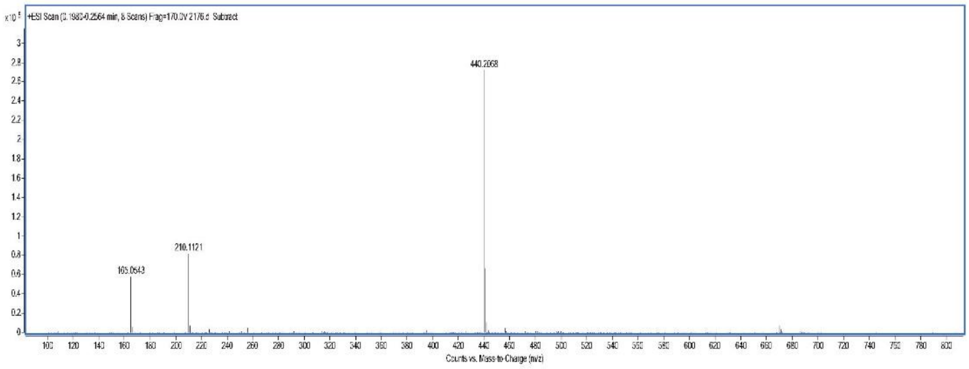

Supplementary Figure S50. HR-ESI-MS spectrum of **5**.

Qualitative Analysis Report

**Data Filename**	2458.d	**Sample Name**	L 12
**Instrument Name**	TOF G6230A	**Acquired Time**	2023-04-23
**Acq Method**	YCL.M	**Acquired SW**	6200 series TOF/6500 series
**IRM Calibration Status**	**Success**		
**User Chormatograms**			

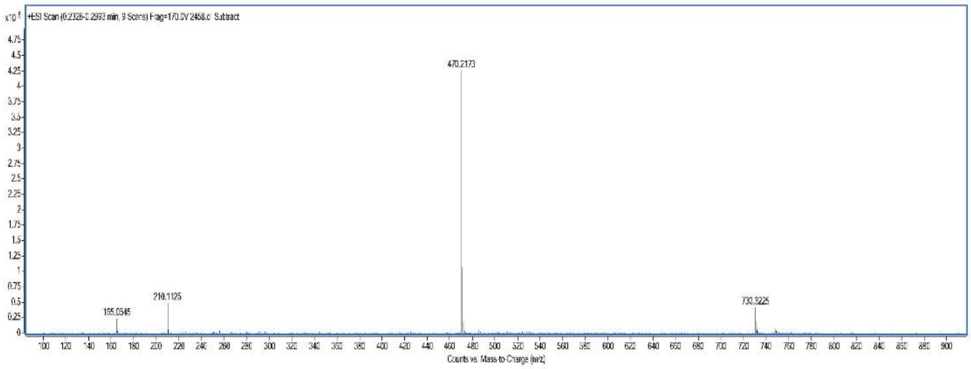

Supplementary Figure S51. HR-ESI-MS spectrum of **6**.

Qualitative Analysis Report

**Data Filename**	2459.d	**Sample Name**	L 14
**Instrument Name**	TOF G6230A	**Acquired Time**	2023-04-23
**Acq Method**	YCL.M	**Acquired SW**	6200 series TOF/6500 series
**IRM Calibration Status**	**Success**		
**User Chormatograms**			

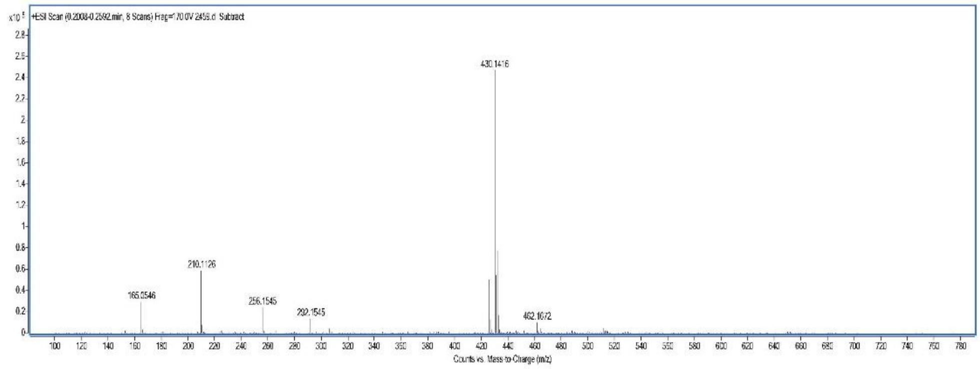

Supplementary Figure S52. HR-ESI-MS spectrum of **7**.

Qualitative Analysis Report

**Data Filename**	2460.d	**Sample Name**	L 16
**Instrument Name**	TOF G6230A	**Acquired Time**	2023-04-23
**Acq Method**	YCL.M	**Acquired SW**	6200 series TOF/6500 series
**IRM Calibration Status**	**Success**		
**User Chormatograms**			

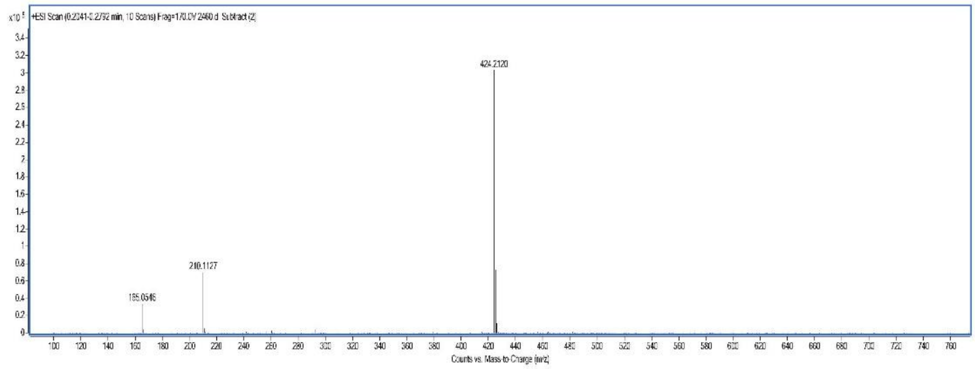

Supplementary Figure S53. HR-ESI-MS spectrum of **8**.

Qualitative Analysis Report

**Data Filename**	2177.d	**Sample Name**	L 18
**Instrument Name**	TOF G6230A	**Acquired Time**	2023-04-13
**Acq Method**	YCL.M	**Acquired SW**	6200 series TOF/6500 series
**IRM Calibration Status**	**Success**		
**User Chormatograms**			

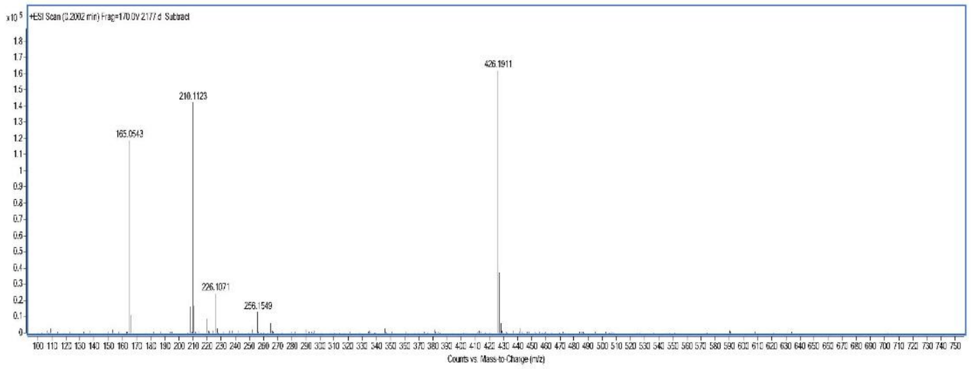

Supplementary Figure S54. HR-ESI-MS spectrum of **9**.

Qualitative Analysis Report

**Data Filename**	2461.d	**Sample Name**	L 20
**Instrument Name**	TOF G6230A	**Acquired Time**	2023-04-23
**Acq Method**	YCL.M	**Acquired SW**	6200 series TOF/6500 series
**IRM Calibration Status**	**Success**		
**User Chormatograms**			

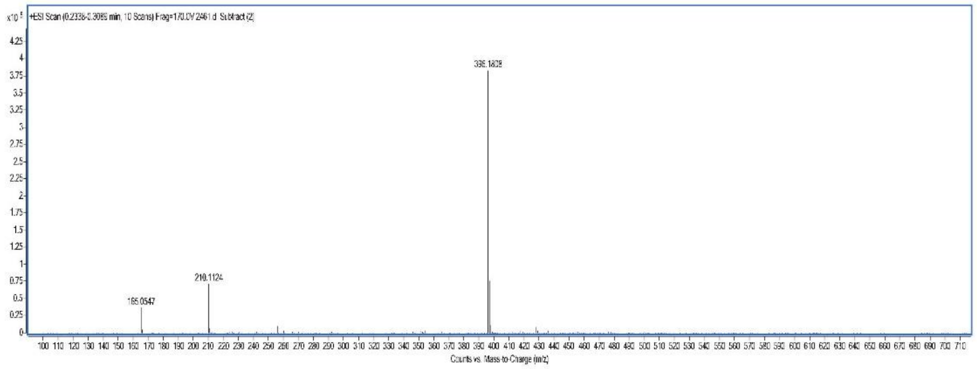

Supplementary Figure S55. HR-ESI-MS spectrum of **10**.

Qualitative Analysis Report

**Data Filename**	2178.d	**Sample Name**	L 22
**Instrument Name**	TOF G6230A	**Acquired Time**	2023-04-13
**Acq Method**	YCL.M	**Acquired SW**	6200 series TOF/6500 series
**IRM Calibration Status**	**Success**		
**User Chormatograms**			

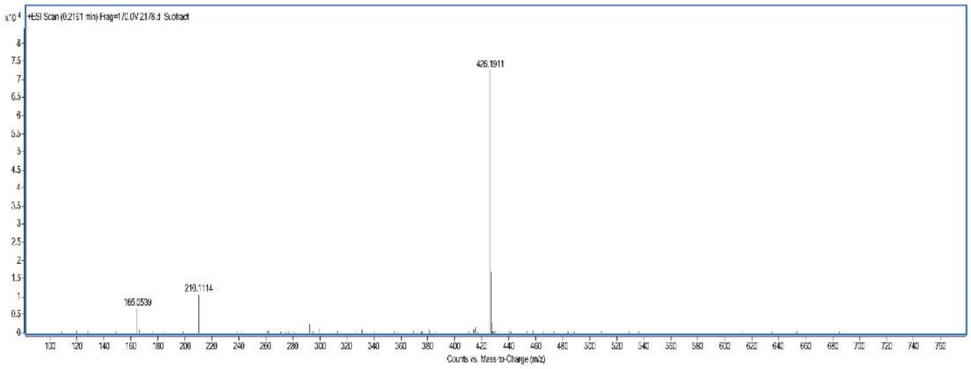

Supplementary Figure S56. HR-ESI-MS spectrum of **11**.

Qualitative Analysis Report

**Data Filename**	3456.d	**Sample Name**	L 24
**Instrument Name**	TOF G6230A	**Acquired Time**	2023-05-29
**Acq Method**	YCL.M	**Acquired SW**	6200 series TOF/6500 series
**IRM Calibration Status**	**Success**		
**User Chormatograms**			

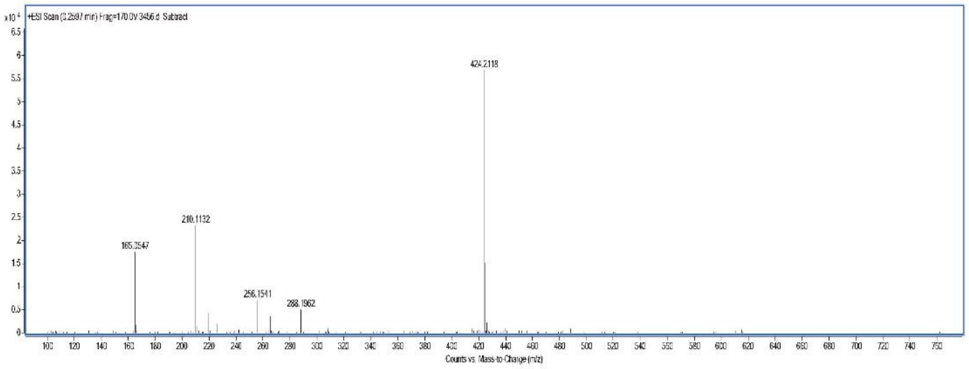

Supplementary Figure S57. HR-ESI-MS spectrum of **12**.

Qualitative Analysis Report

**Data Filename**	2464.d	**Sample Name**	L 26
**Instrument Name**	TOF G6230A	**Acquired Time**	2023-04-23
**Acq Method**	YCL.M	**Acquired SW**	6200 series TOF/6500 series
**IRM Calibration Status**	**Success**		
**User Chormatograms**			

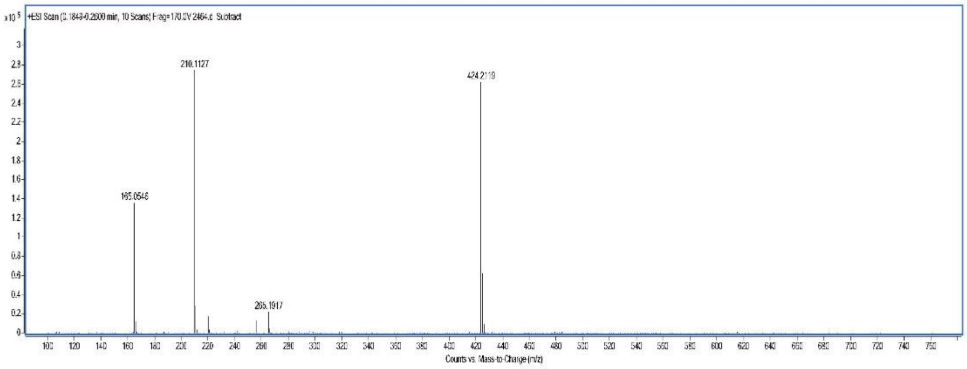

Supplementary Figure S58. HR-ESI-MS spectrum of **13**.

Qualitative Analysis Report

**Data Filename**	3457.d	**Sample Name**	L 28
**Instrument Name**	TOF G6230A	**Acquired Time**	2023-05-29
**Acq Method**	YCL.M	**Acquired SW**	6200 series TOF/6500 series
**IRM Calibration Status**	**Success**		
**User Chormatograms**			

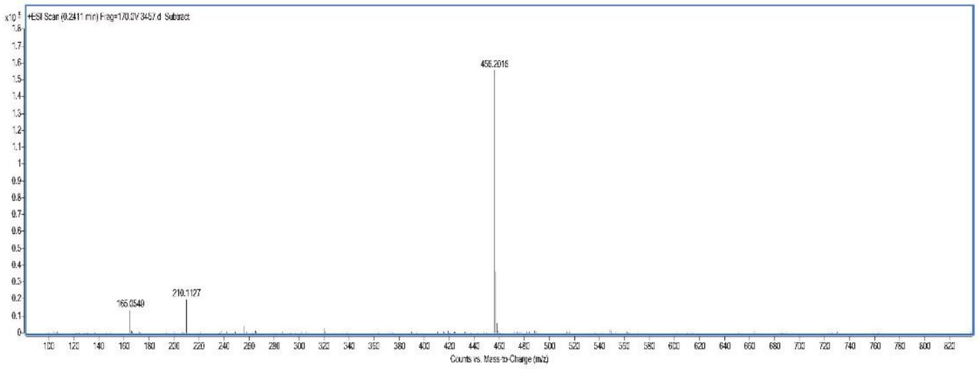

Supplementary Figure S59. HR-ESI-MS spectrum of **14**.

Qualitative Analysis Report

**Data Filename**	2462.d	**Sample Name**	L 30
**Instrument Name**	TOF G6230A	**Acquired Time**	2023-04-23
**Acq Method**	YCL.M	**Acquired SW**	6200 series TOF/6500 series
**IRM Calibration Status**	**Success**		
**User Chormatograms**			

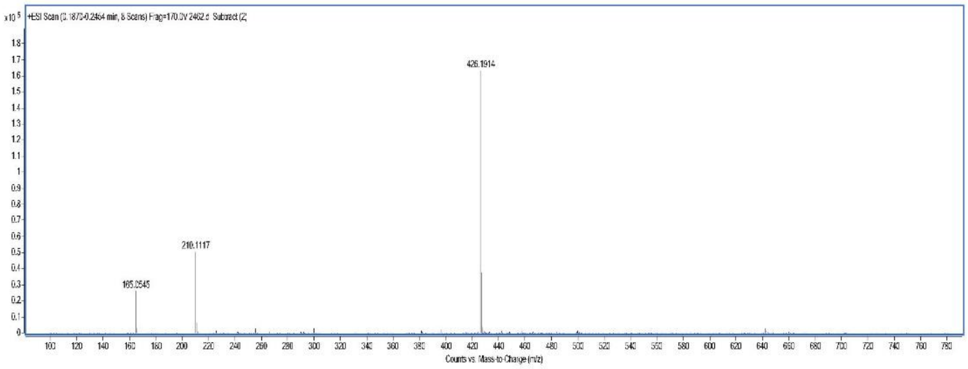

Supplementary Figure S60. HR-ESI-MS spectrum of **15**.

Qualitative Analysis Report

**Data Filename**	0837.d	**Sample Name**	YA 1
**Instrument Name**	TOF G6230A	**Acquired Time**	2024-03-12
**Acq Method**	YCL.M	**Acquired SW**	6200 series TOF/6500 series
**IRM Calibration Status**	**Success**		
**User Chormatograms**			

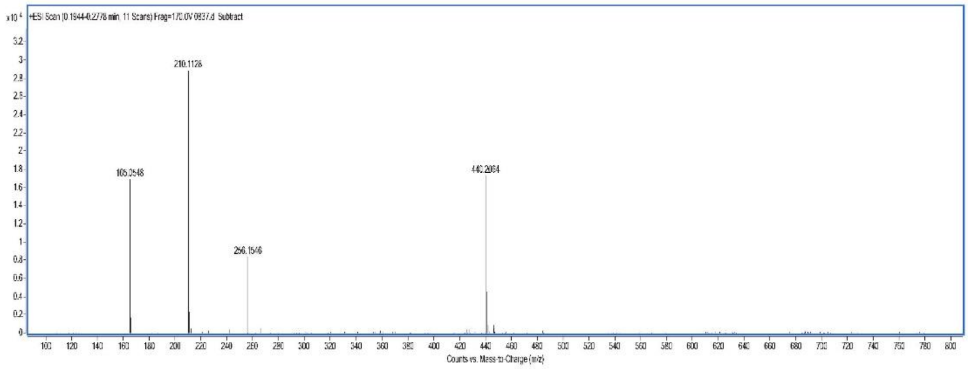

Supplementary Figure S61. HR-ESI-MS spectrum of **1a**.

Qualitative Analysis Report

**Data Filename**	0822.d	**Sample Name**	YB-1
**Instrument Name**	TOF G6230A	**Acquired Time**	2024-03-12
**Acq Method**	YCL.M	**Acquired SW**	6200 series TOF/6500 series
**IRM Calibration Status**	**Success**		
**User Chormatograms**			

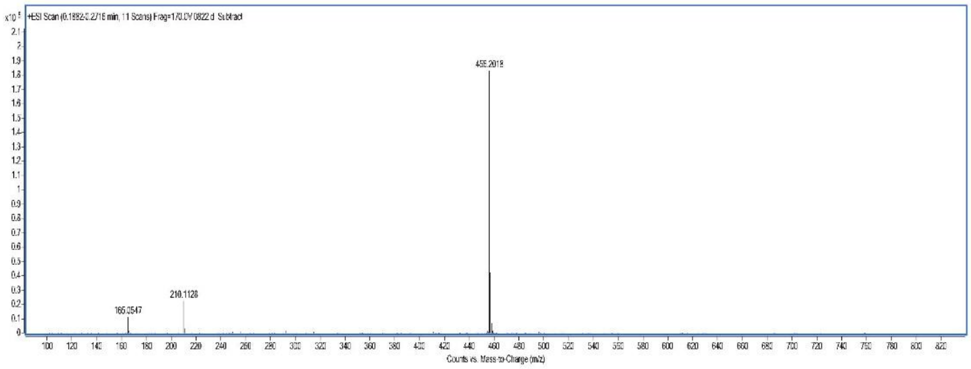

Supplementary Figure S62. HR-ESI-MS spectrum of **2a**.

Qualitative Analysis Report

**Data Filename**	0823.d	**Sample Name**	YC-1
**Instrument Name**	TOF G6230A	**Acquired Time**	2024-03-12
**Acq Method**	YCL.M	**Acquired SW**	6200 series TOF/6500 series
**IRM Calibration Status**	**Success**		
**User Chormatograms**			

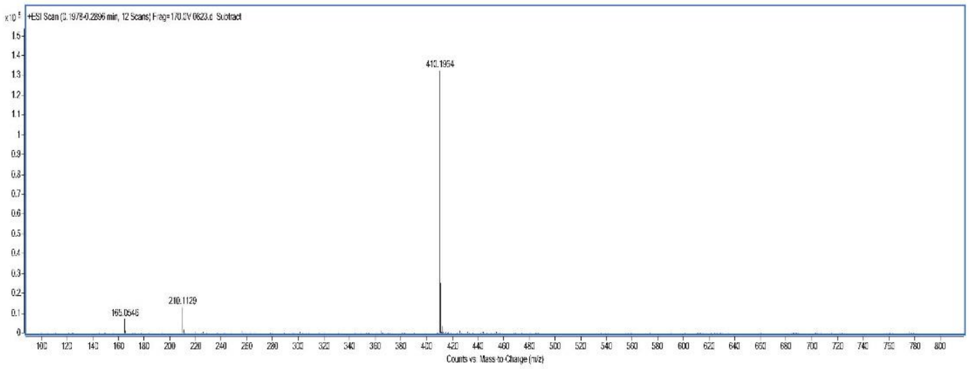

Supplementary Figure S63. HR-ESI-MS spectrum of **3a**.

Qualitative Analysis Report

**Data Filename**	0825.d	**Sample Name**	YD-1
**Instrument Name**	TOF G6230A	**Acquired Time**	2024-03-12
**Acq Method**	YCL.M	**Acquired SW**	6200 series TOF/6500 series
**IRM Calibration Status**	**Success**		
**User Chormatograms**			

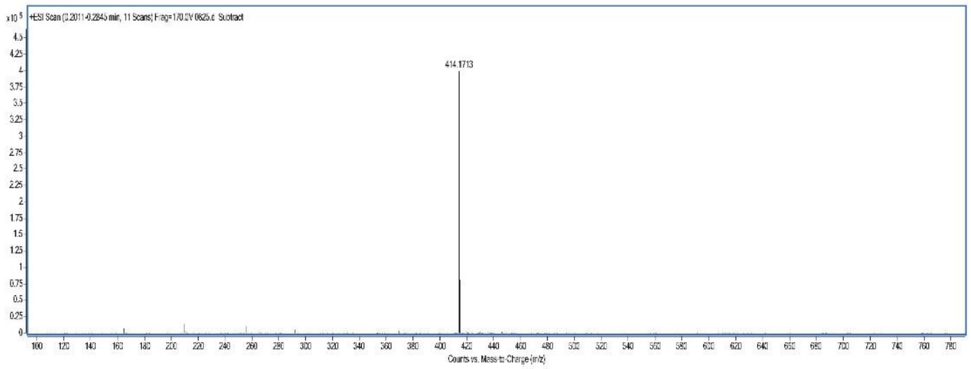

Supplementary Figure S64. HR-ESI-MS spectrum of **4a**.

Qualitative Analysis Report

**Data Filename**	0826.d	**Sample Name**	YE-1
**Instrument Name**	TOF G6230A	**Acquired Time**	2024-03-12
**Acq Method**	YCL.M	**Acquired SW**	6200 series TOF/6500 series
**IRM Calibration Status**	**Success**		
**User Chormatograms**			

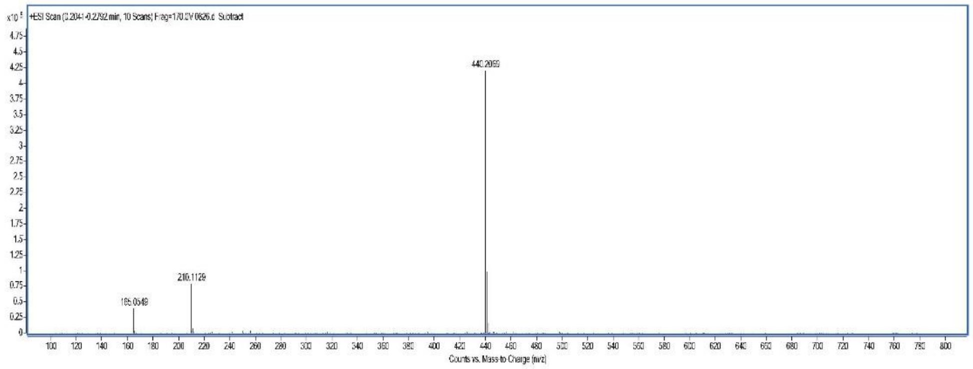

Supplementary Figure S65. HR-ESI-MS spectrum of **5a**.

Qualitative Analysis Report

**Data Filename**	0827.d	**Sample Name**	YF-1
**Instrument Name**	TOF G6230A	**Acquired Time**	2024-03-12
**Acq Method**	YCL.M	**Acquired SW**	6200 series TOF/6500 series
**IRM Calibration Status**	**Success**		
**User Chormatograms**			

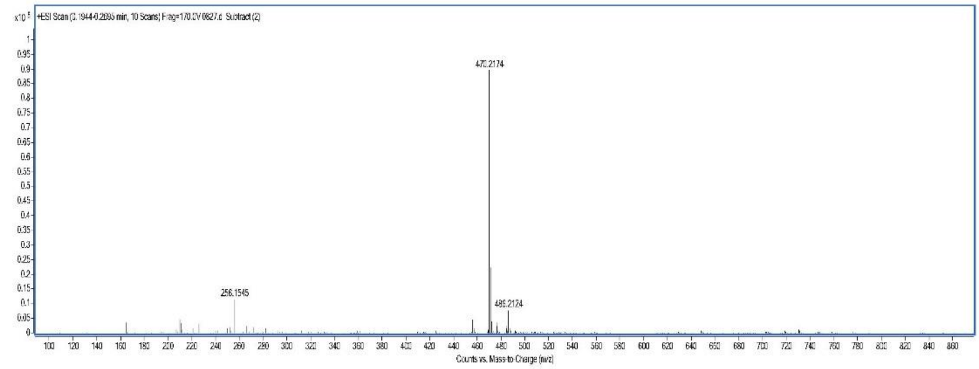

Supplementary Figure S66. HR-ESI-MS spectrum of **6a**.

Qualitative Analysis Report

**Data Filename**	0828.d	**Sample Name**	YG-1
**Instrument Name**	TOF G6230A	**Acquired Time**	2024-03-12
**Acq Method**	YCL.M	**Acquired SW**	6200 series TOF/6500 series
**IRM Calibration Status**	**Success**		
**User Chormatograms**			

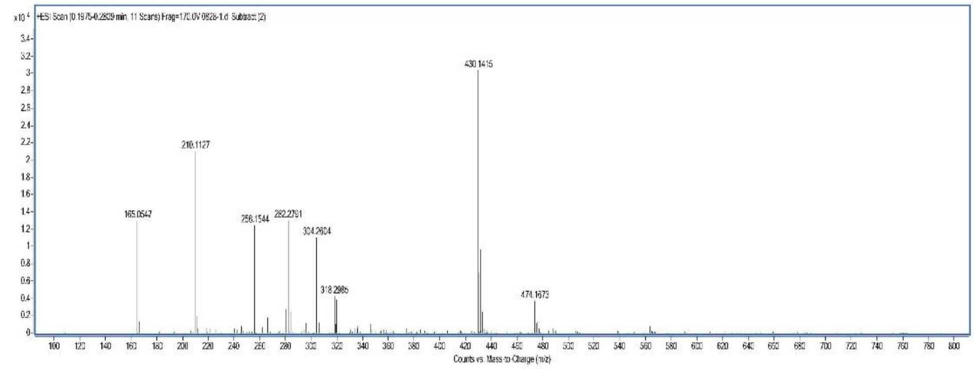

Supplementary Figure S67. HR-ESI-MS spectrum of **7a**.

Qualitative Analysis Report

**Data Filename**	0829.d	**Sample Name**	YJ-1
**Instrument Name**	TOF G6230A	**Acquired Time**	2024-03-12
**Acq Method**	YCL.M	**Acquired SW**	6200 series TOF/6500 series
**IRM Calibration Status**	**Success**		
**User Chormatograms**			

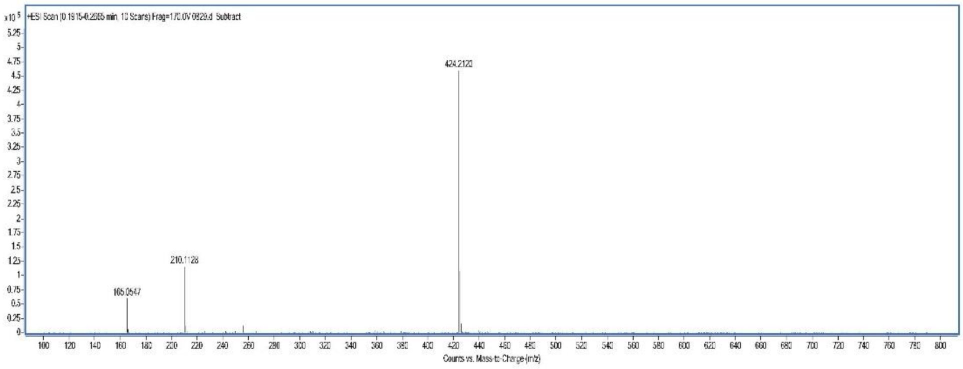

Supplementary Figure S68. HR-ESI-MS spectrum of **8a**.

Qualitative Analysis Report

**Data Filename**	0830.d	**Sample Name**	YJ-1
**Instrument Name**	TOF G6230A	**Acquired Time**	2024-03-12
**Acq Method**	YCL.M	**Acquired SW**	6200 series TOF/6500 series
**IRM Calibration Status**	**Success**		
**User Chormatograms**			

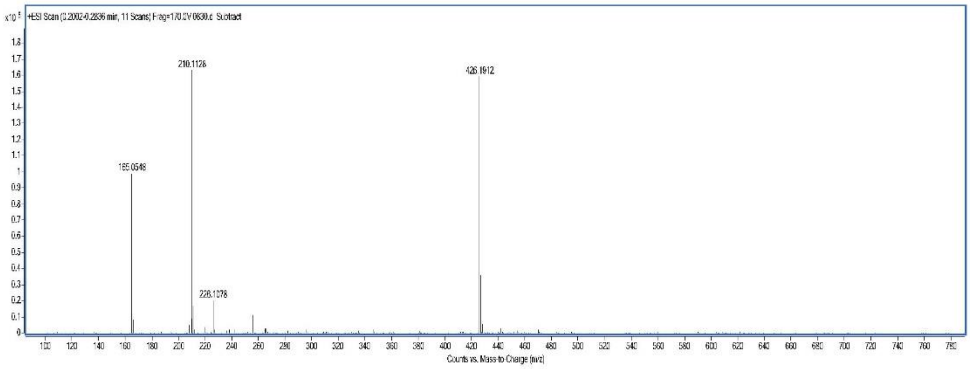

Supplementary Figure S69. HR-ESI-MS spectrum of **9a**.

Qualitative Analysis Report

**Data Filename**	0831.d	**Sample Name**	YK 1
**Instrument Name**	TOF G6230A	**Acquired Time**	2024-03-12
**Acq Method**	YCL.M	**Acquired SW**	6200 series TOF/6500 series
**IRM Calibration Status**	**Success**		
**User Chormatograms**			

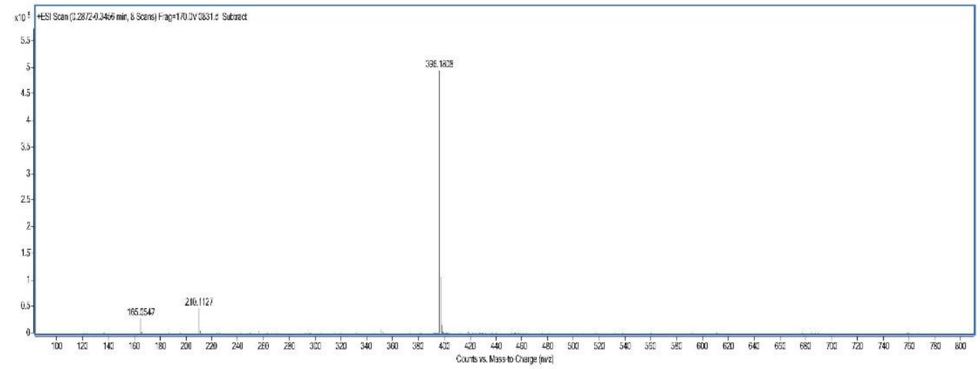

Supplementary Figure S70. HR-ESI-MS spectrum of **10a**.

Qualitative Analysis Report

**Data Filename**	0832.d	**Sample Name**	YL 1
**Instrument Name**	TOF G6230A	**Acquired Time**	2024-03-12
**Acq Method**	YCL.M	**Acquired SW**	6200 series TOF/6500 series
**IRM Calibration Status**	**Success**		
**User Chormatograms**			

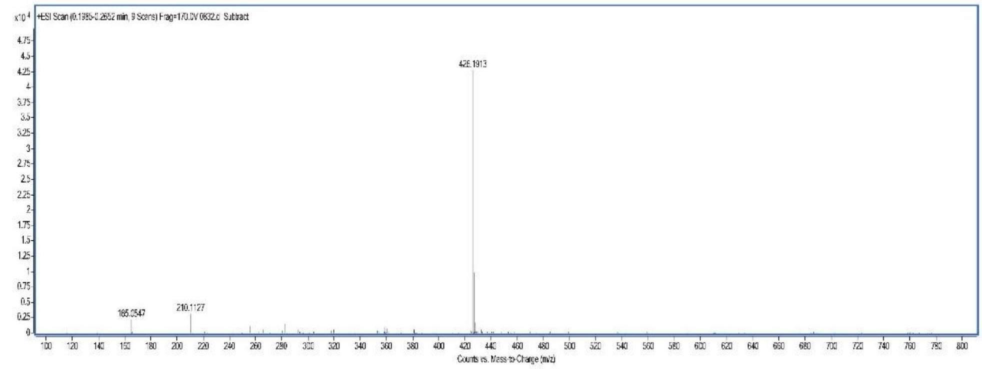

Supplementary Figure S71. HR-ESI-MS spectrum of **11a**.

Qualitative Analysis Report

**Data Filename**	0833.d	**Sample Name**	YO 1
**Instrument Name**	TOF G6230A	**Acquired Time**	2024-03-12
**Acq Method**	YCL.M	**Acquired SW**	6200 series TOF/6500 series
**IRM Calibration Status**	**Success**		
**User Chormatograms**			

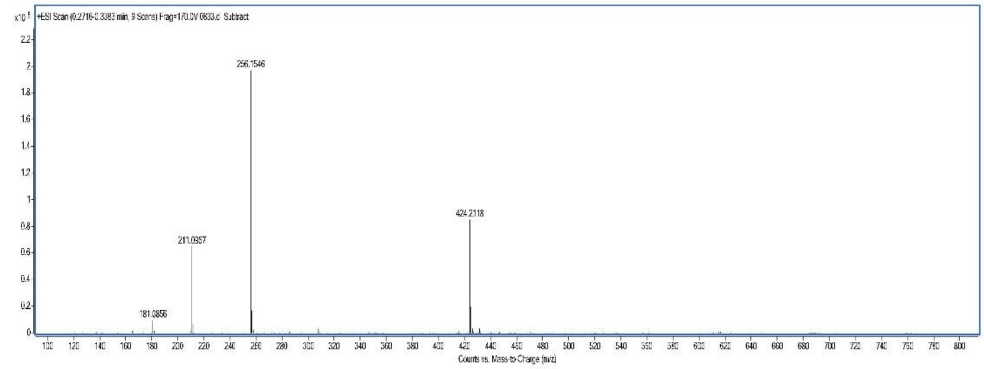

Supplementary Figure S72. HR-ESI-MS spectrum of **12a**.

Qualitative Analysis Report

**Data Filename**	0834.d	**Sample Name**	YP 1
**Instrument Name**	TOF G6230A	**Acquired Time**	2024-03-12
**Acq Method**	YCL.M	**Acquired SW**	6200 series TOF/6500 series
**IRM Calibration Status**	**Success**		
**User Chormatograms**			

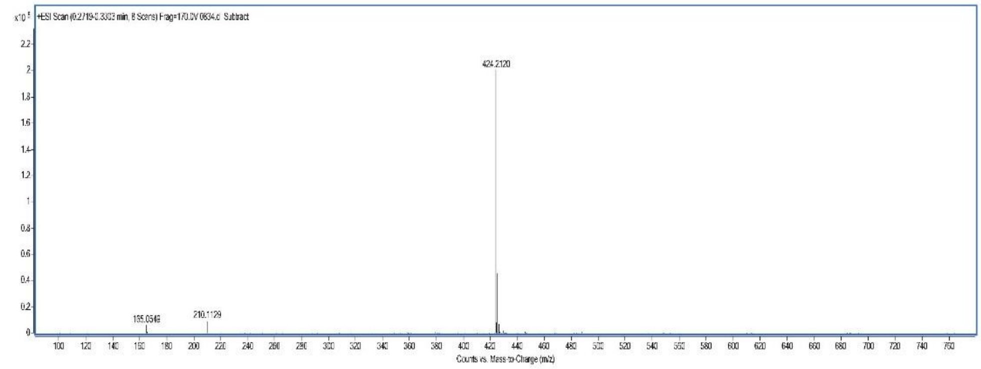

Supplementary Figure S73. HR-ESI-MS spectrum of **13a**.

Qualitative Analysis Report

**Data Filename**	0835.d	**Sample Name**	YU-1
**Instrument Name**	TOF G6230A	**Acquired Time**	2024-03-12
**Acq Method**	YCL.M	**Acquired SW**	6200 series TOF/6500 series
**IRM Calibration Status**	**Success**		
**User Chormatograms**			

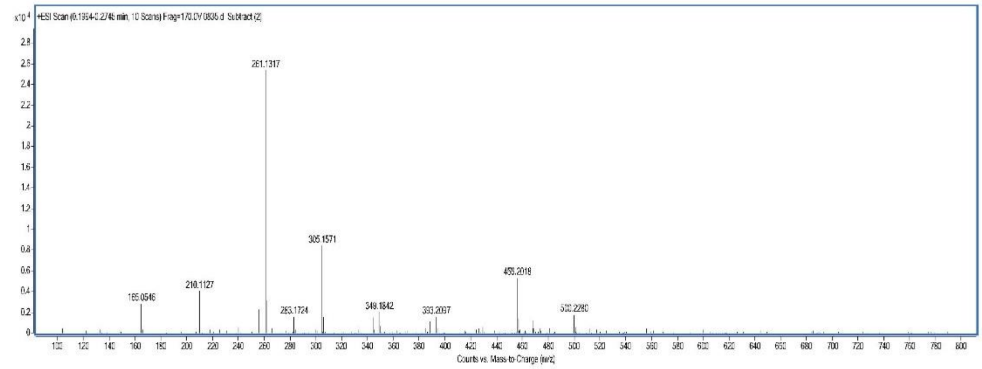

Supplementary Figure S74. HR-ESI-MS spectrum of **14a.**

Qualitative Analysis Report

**Data Filename**	0836.d	**Sample Name**	YZ-1
**Instrument Name**	TOF G6230A	**Acquired Time**	2024-03-12
**Acq Method**	YCL.M	**Acquired SW**	6200 series TOF/6500 series
**IRM Calibration Status**	**Success**		
**User Chormatograms**			

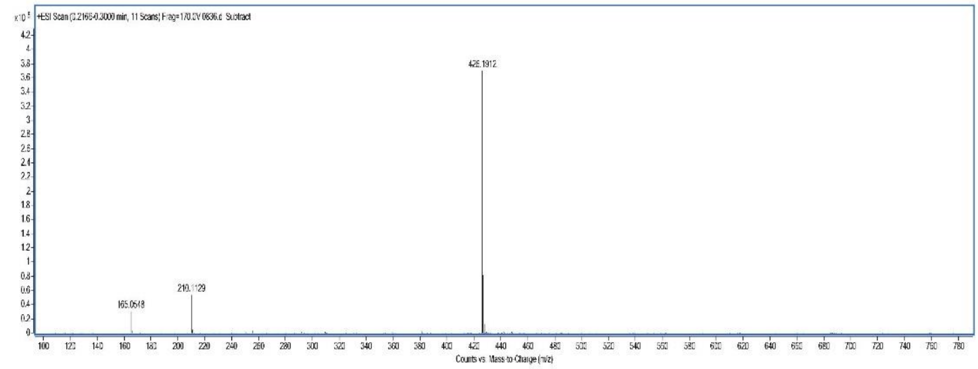

Supplementary Figure S75. HR-ESI-MS spectrum of **15a.**

Section 4: Concentration-absorbance curve of curcumin derivative acetates **1a-15a**.

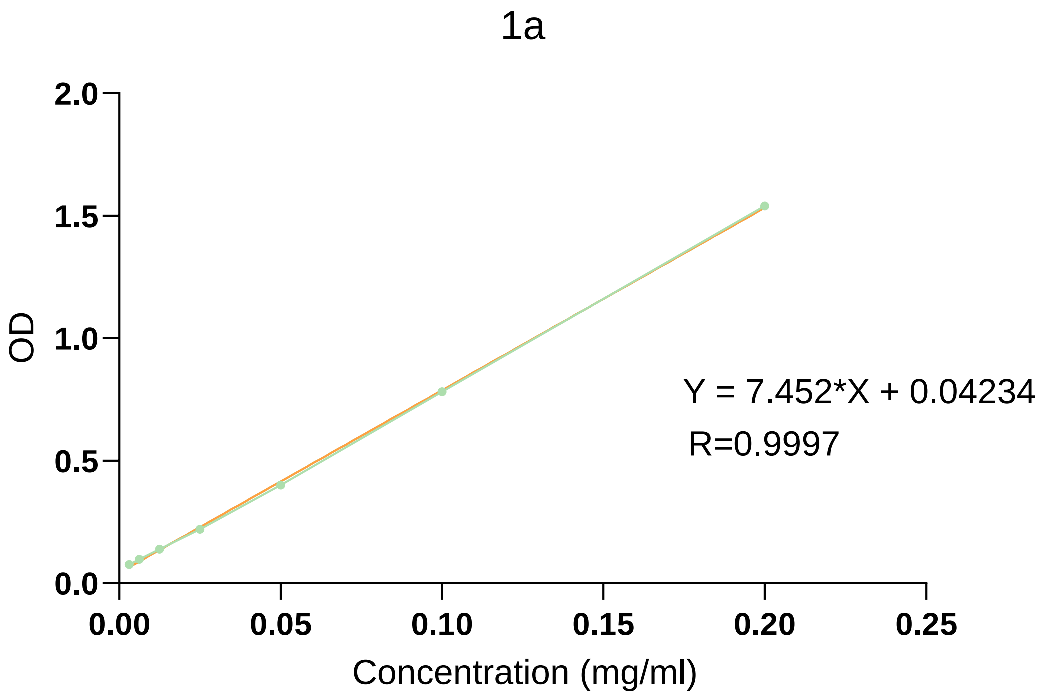

Supplementary Figure S76. Concentration-absorbance curve of **1a**.

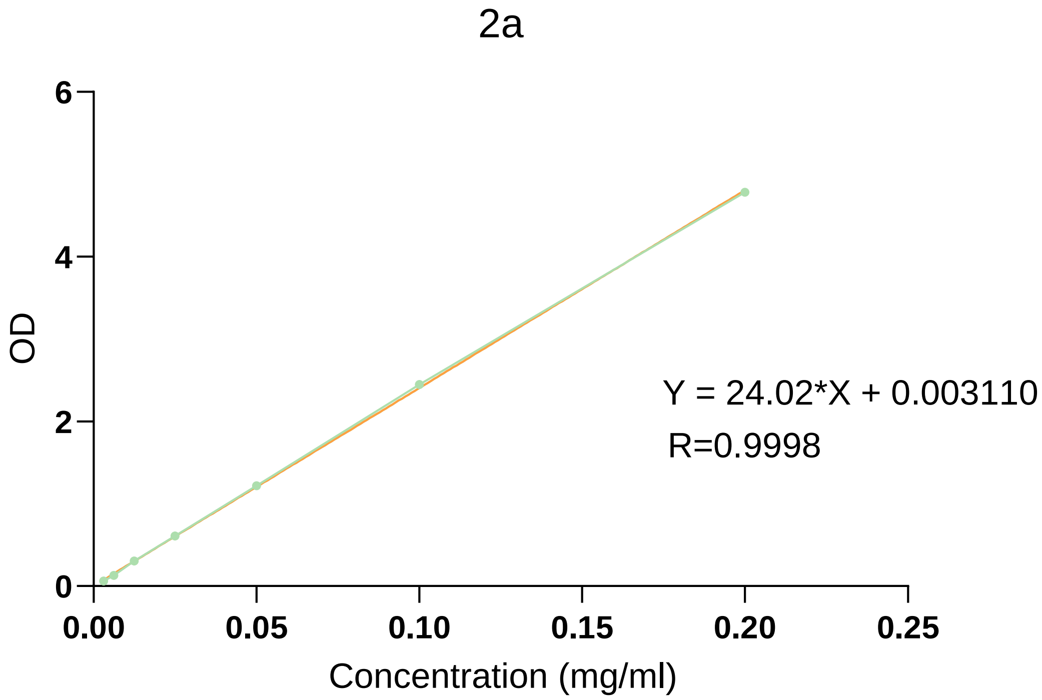

Supplementary Figure S77. Concentration-absorbance curve of **2a**.

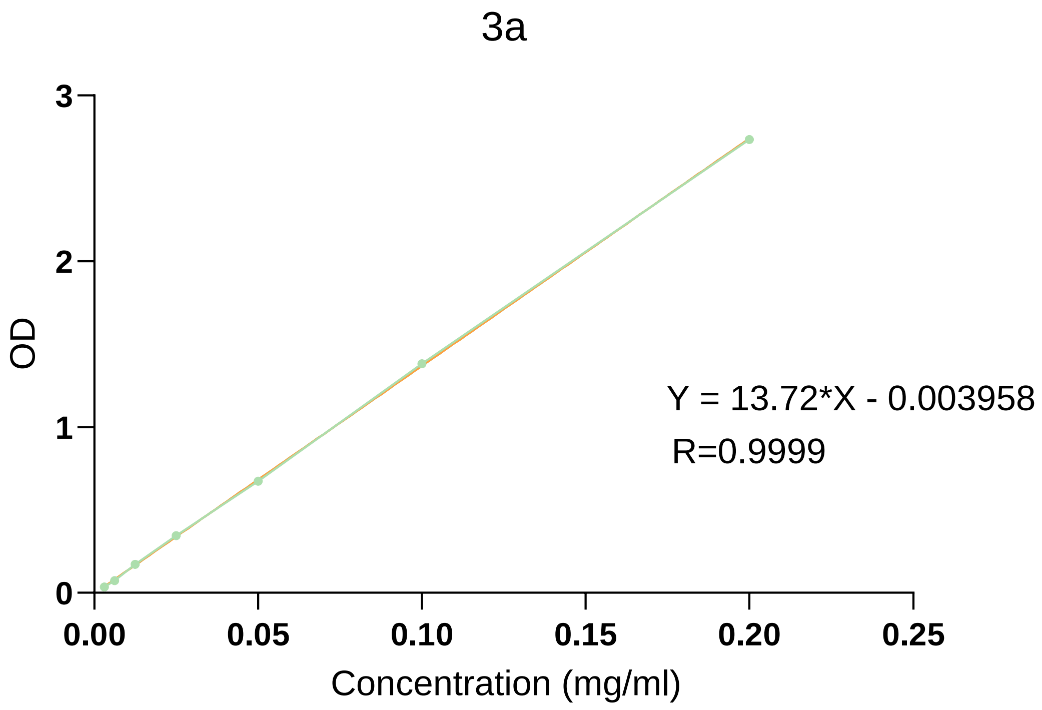

Supplementary Figure S78. Concentration-absorbance curve of **3a**.

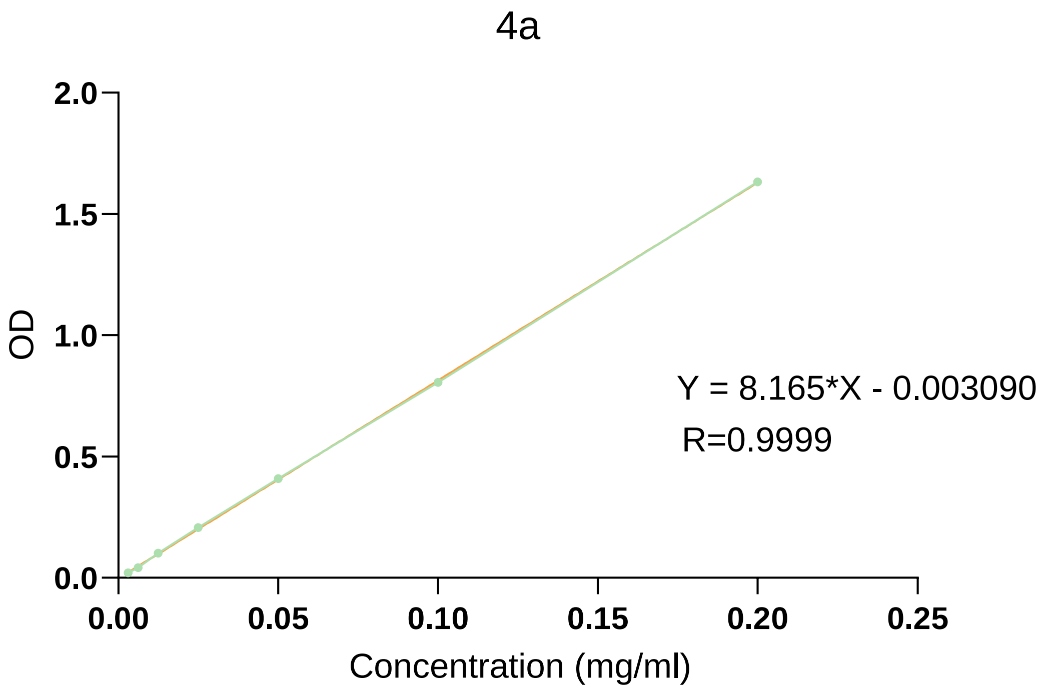

Supplementary Figure S79. Concentration-absorbance curve of **4a**.

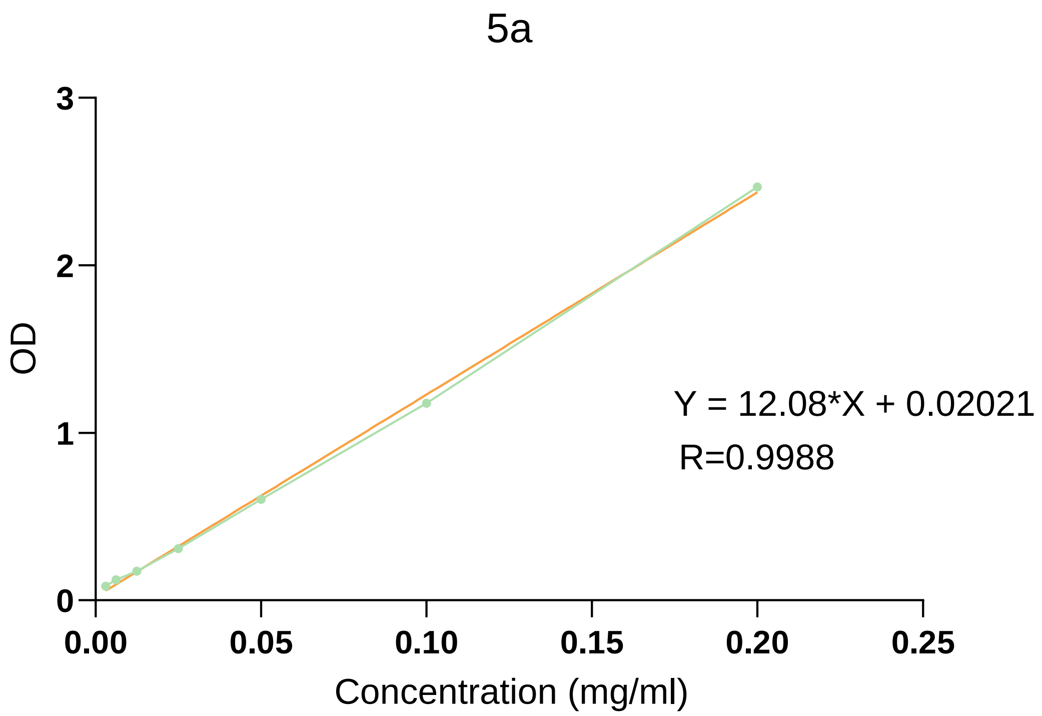

Supplementary Figure S80. Concentration-absorbance curve of **5a**.

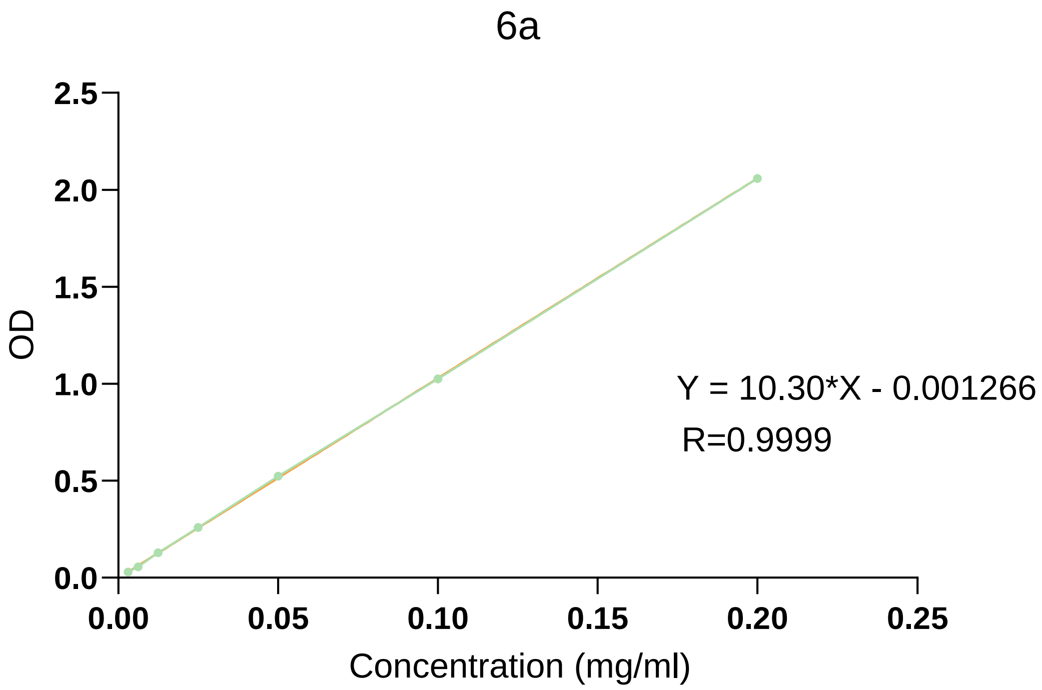

Supplementary Figure S81. Concentration-absorbance curve of **6a**.

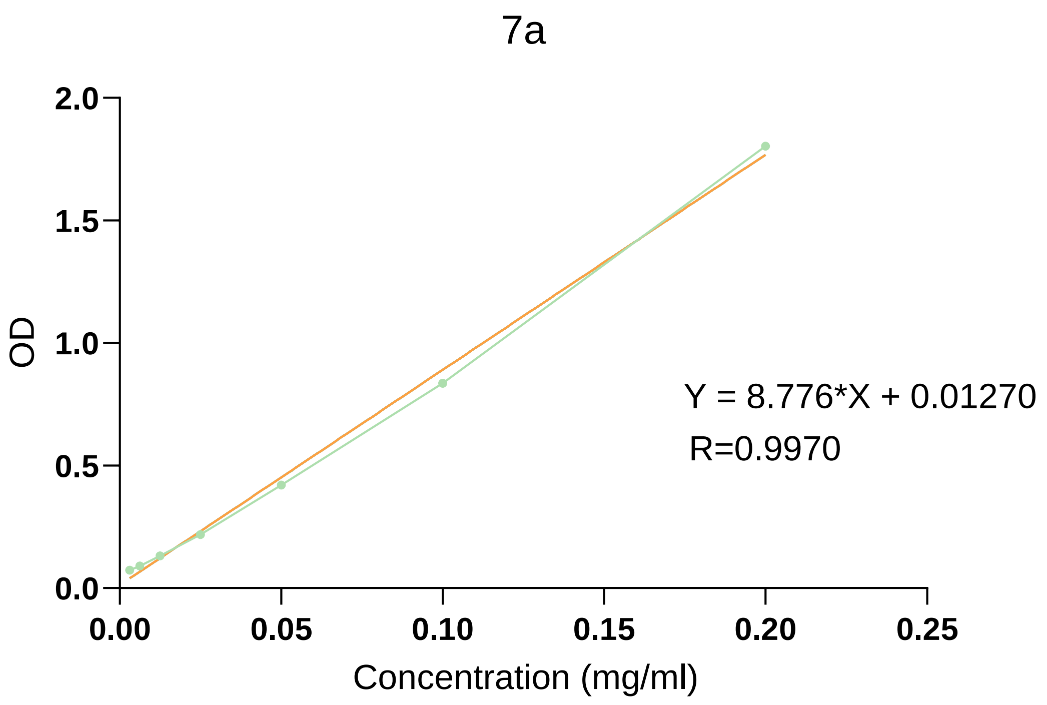

Supplementary Figure S82. Concentration-absorbance curve of **7a**.

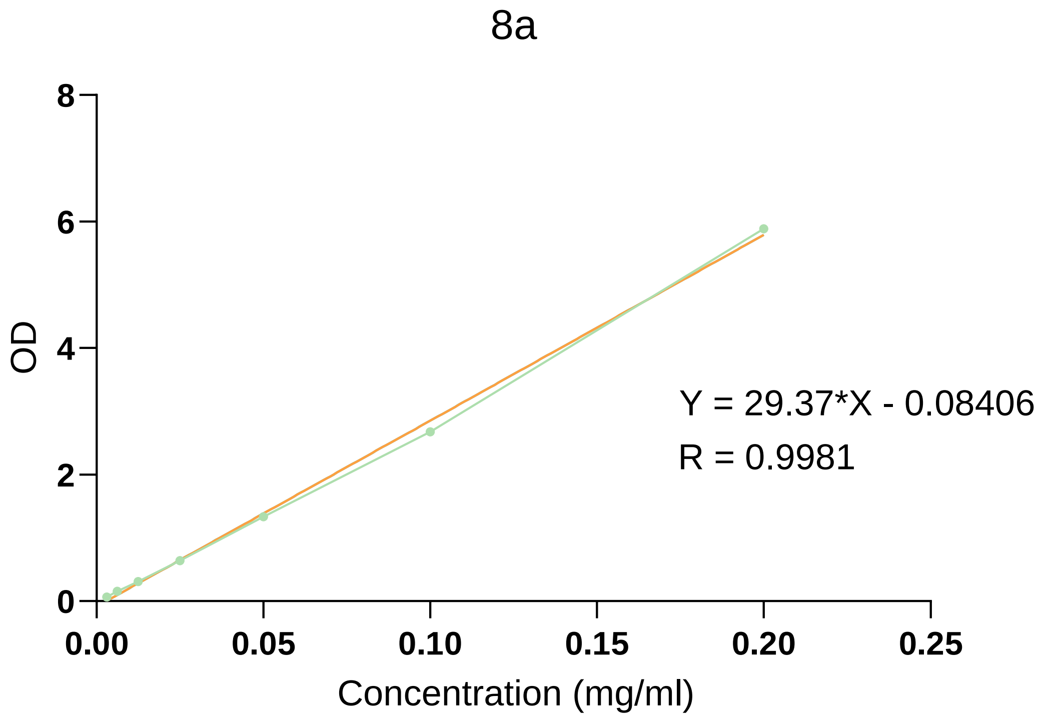

Supplementary Figure S83. Concentration-absorbance curve of **8a**.

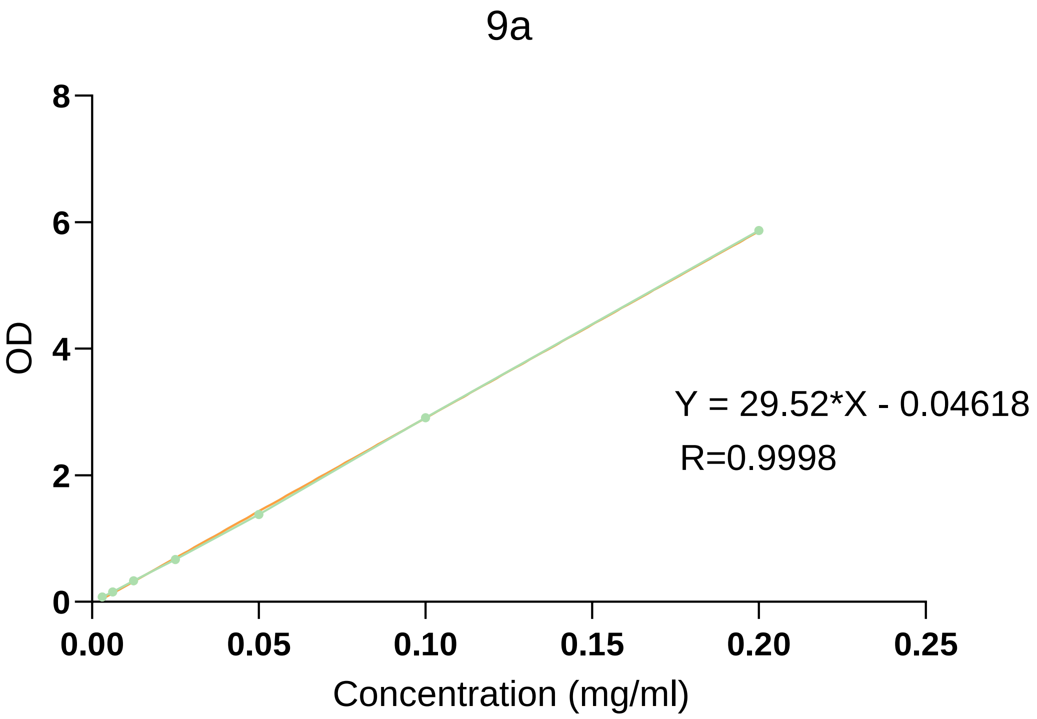

Supplementary Figure S84. Concentration-absorbance curve of **9a**.

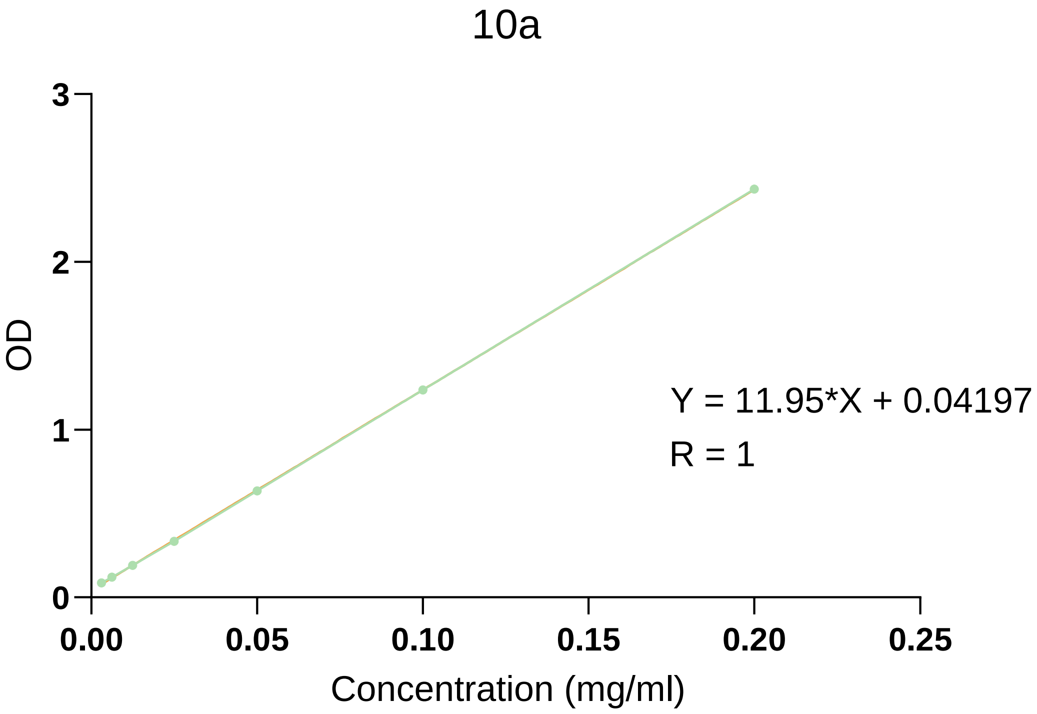

Supplementary Figure S85. Concentration-absorbance curve of **10a**.

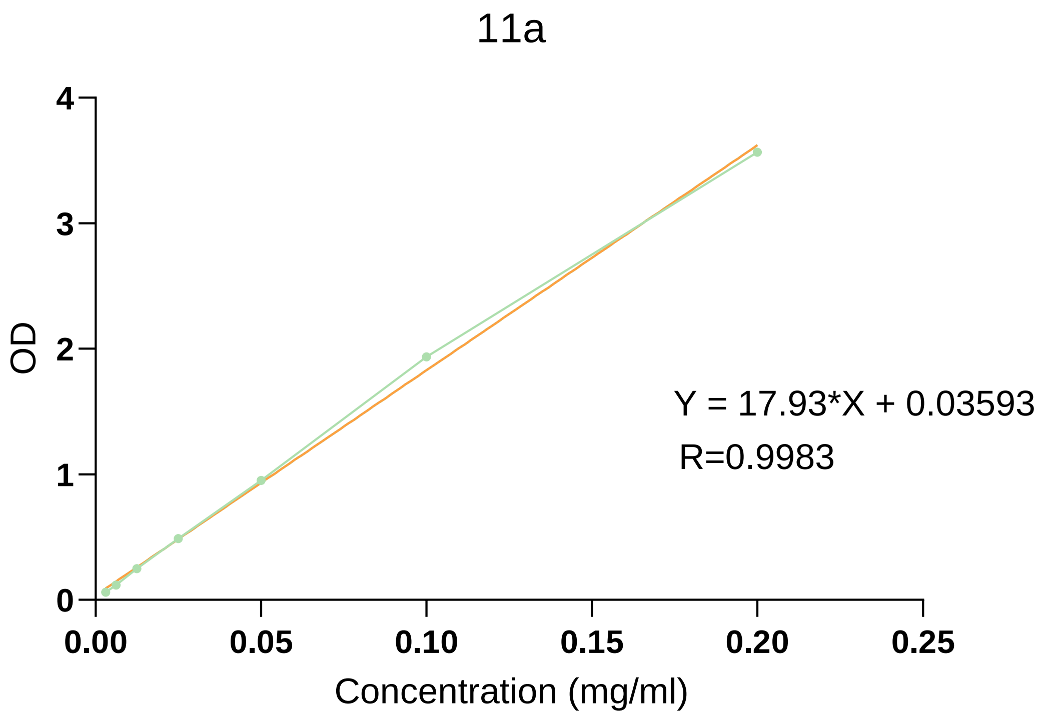

Supplementary Figure S86. Concentration-absorbance curve of **11a**.

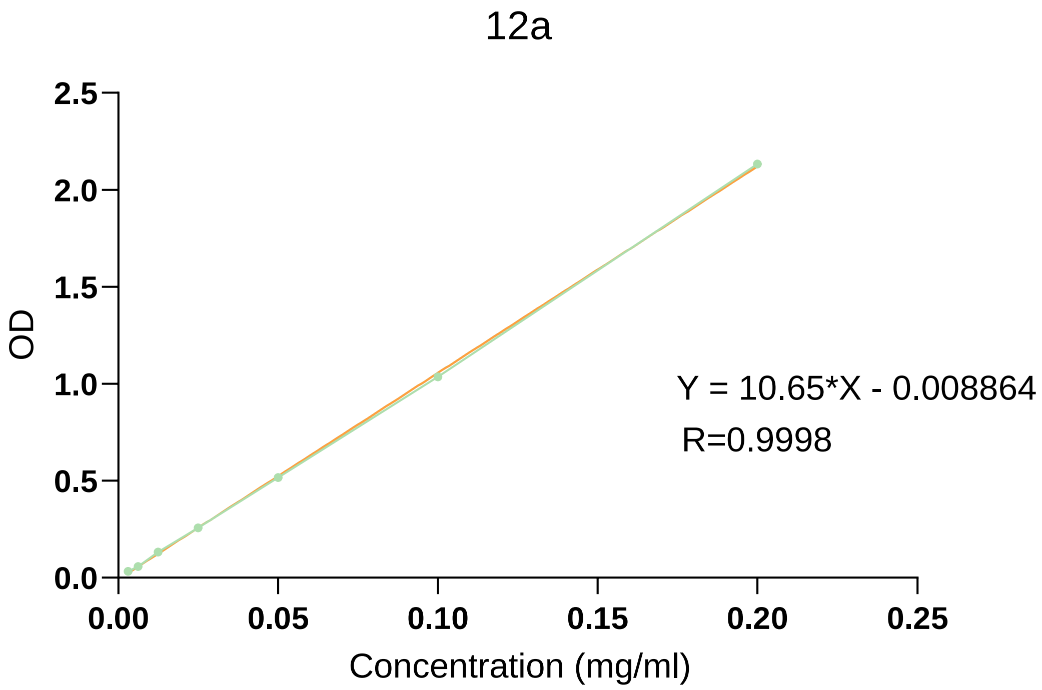

Supplementary Figure S87. Concentration-absorbance curve of **12a**.

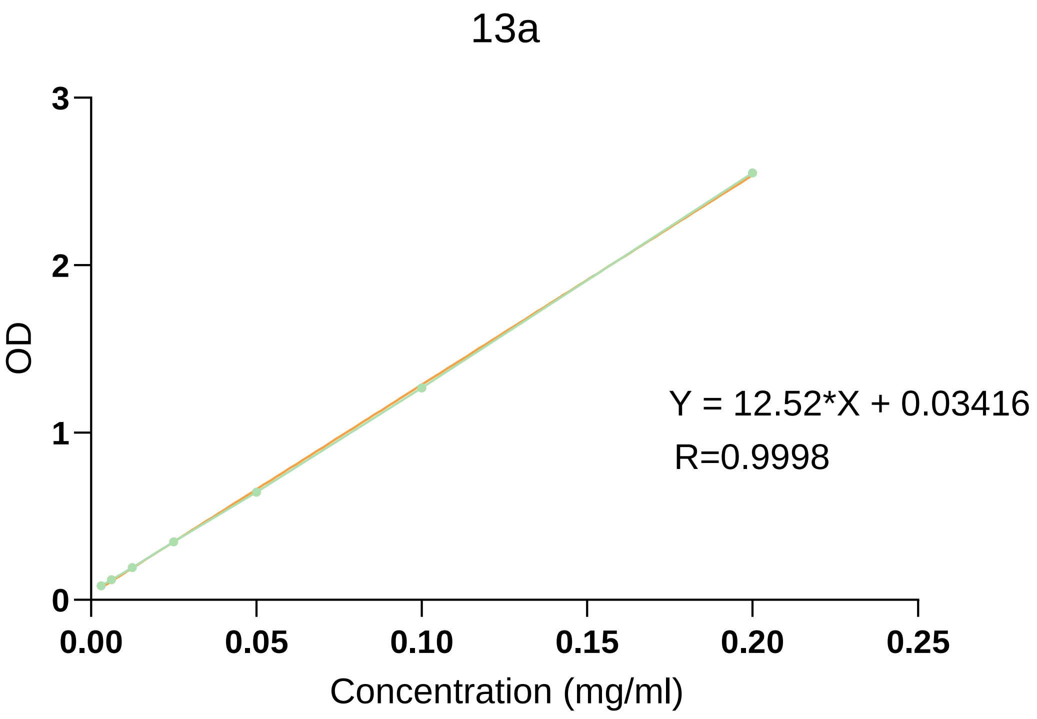

Supplementary Figure S88. Concentration-absorbance curve of **13a**.

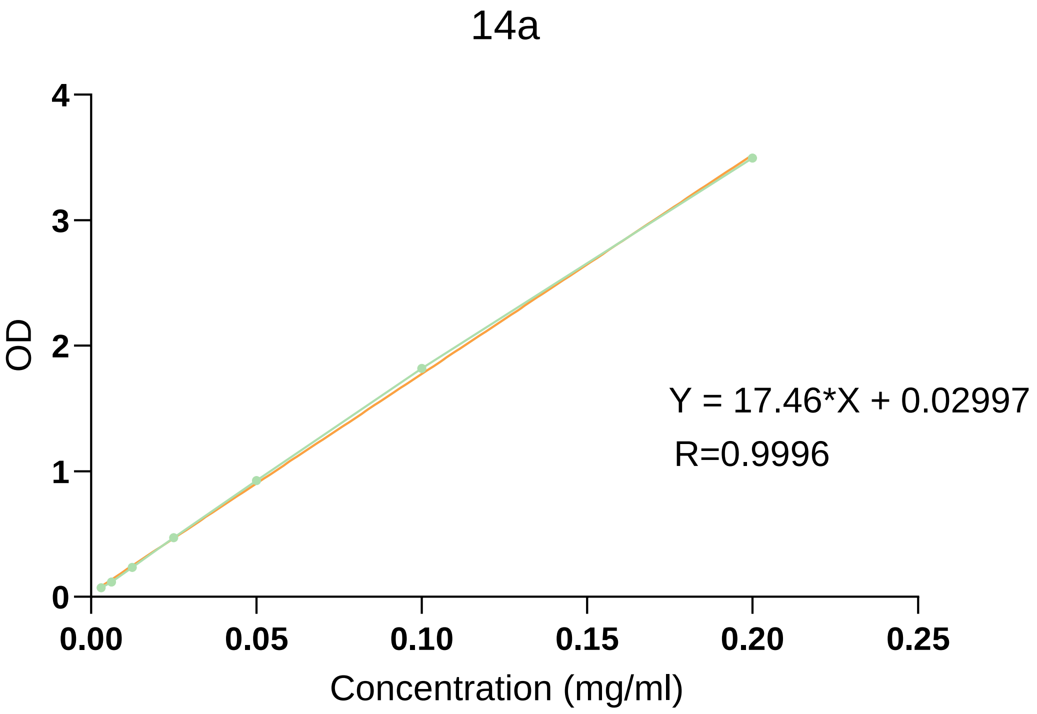

Supplementary Figure S89. Concentration-absorbance curve of **14a**.

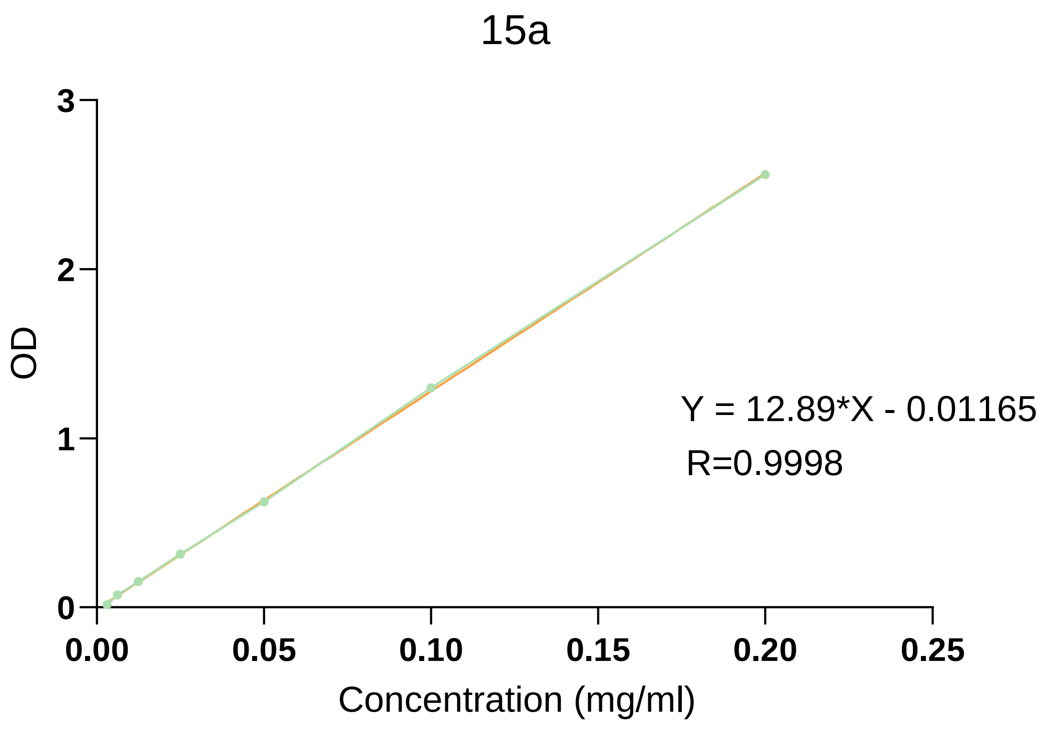

Supplementary Figure S90. Concentration-absorbance curve of **15a**.
